# A mouse embryonic stem cell bank for inducible overexpression of human chromosome 21 genes

**DOI:** 10.1186/gb-2010-11-6-r64

**Published:** 2010-06-22

**Authors:** Rossella De Cegli, Antonio Romito, Simona Iacobacci, Lei Mao, Mario Lauria, Anthony O Fedele, Joachim Klose, Christelle Borel, Patrick Descombes, Stylianos E Antonarakis, Diego di Bernardo, Sandro Banfi, Andrea Ballabio, Gilda Cobellis

**Affiliations:** 1 Telethon Institute of Genetics and Medicine, Via P. Castellino 111, Napoli, 80131, Italy; 2 Current address: Université Paris Diderot - Paris 7, Paris Cedex 13, Paris, 75205, France; 3 Institut für Humangenetik Charité, Campus Virchow-Klinikum, Universitätsmedizin Berlin, Augustenburger Platz 1, Berlin, D-13353, Germany; 4 Current address: Lysosomal Diseases Research Unit, SA Pathology, 72 King William Road, North Adelaide, South Australia, 5006, Australia; 5 Department of Genetic Medicine and Development, University of Geneva Medical School, 1 rue Michel-Servet, Geneva, CH-1211, Switzerland; 6 Genomics Platform, University of Geneva Medical School, 1 rue Michel-Servet, Geneva, CH-1211, Switzerland; 7 Current address: Dipartimento di Patologia Generale, Seconda Universita' di Napoli, Via De Crecchio 7, Napoli, 80100, Italy

## Abstract

**Background:**

Dosage imbalance is responsible for several genetic diseases, among which Down syndrome is caused by the trisomy of human chromosome 21.

**Results:**

To elucidate the extent to which the dosage imbalance of specific human chromosome 21 genes perturb distinct molecular pathways, we developed the first mouse embryonic stem (ES) cell bank of human chromosome 21 genes. The human chromosome 21-mouse ES cell bank includes, in triplicate clones, 32 human chromosome 21 genes, which can be overexpressed in an inducible manner. Each clone was transcriptionally profiled in inducing versus non-inducing conditions. Analysis of the transcriptional response yielded results that were consistent with the perturbed gene's known function. Comparison between mouse ES cells containing the whole human chromosome 21 (trisomic mouse ES cells) and mouse ES cells overexpressing single human chromosome 21 genes allowed us to evaluate the contribution of single genes to the trisomic mouse ES cell transcriptome. In addition, for the clones overexpressing the *Runx1 *gene, we compared the transcriptome changes with the corresponding protein changes by mass spectroscopy analysis.

**Conclusions:**

We determined that only a subset of genes produces a strong transcriptional response when overexpressed in mouse ES cells and that this effect can be predicted taking into account the basal gene expression level and the protein secondary structure. We showed that the human chromosome 21-mouse ES cell bank is an important resource, which may be instrumental towards a better understanding of Down syndrome and other human aneuploidy disorders.

## Background

Aneuploidy refers to an abnormal copy number of genomic elements, and is one of the most common causes of morbidity and mortality in humans [1,2]. The importance of aneuploidy is often neglected because most of its effects occur during embryonic and fetal development [3]. Initially, the term aneuploidy was restricted to the presence of supernumerary copies of whole chromosomes, or absence of chromosomes, but this definition has been extended to include deletions or duplications of sub-chromosomal regions [4,5]. Gene dosage imbalance represents the main factor in determining the molecular pathogenesis of aneuploidy disorders [6].

Our interest is focused on the elucidation of the molecular basis of gene dosage imbalance in one of the most clinically relevant and common forms of aneuploidy, Down syndrome (DS). DS, caused by the trisomy of human chromosome 21 (HSA21), is a complex condition characterized by several phenotypic features [6], some of which are present in all patients while others occur only in a fraction of affected individuals. In particular, cognitive impairment, craniofacial dysmorphology and hypotonia are the features present in all DS patients. On the other hand, congenital heart defects occur in only approximately 40% of patients. Moreover, duodenal stenosis/atresia, Hirschsprung disease and acute megakaryocytic leukemia occur 250-, 30- and 300-times more frequently, respectively, in patients with DS than in the general population. Individuals with DS are affected by these phenotypes to a variable extent, implying that many phenotypic features of DS result from quantitative differences in the expression of HSA21 genes. Understanding the mechanisms by which the extra copy of HSA21 leads to the complex and variable phenotypes observed in DS patients [7,8] is a key challenge.

The DS phenotype is clearly the outcome of the extra copy of HSA21. However, this view does not completely address the mechanisms by which the phenotype arises. Korbel *et al*. [9] provided the highest resolution DS phenotype map to date and identified distinct genomic regions that likely contribute to the manifestation of eight DS features. Recent studies suggest that the effect of the elevated expression of particular HSA21 genes is responsible for specific aspects of the DS phenotype. Arron *et al. *[10] showed that some characteristics of the DS phenotype can be related to an increase in dosage expression of two HSA21 genes, namely those encoding the transcriptional activator DSCR1-RCAN1 and the protein kinase DYRK1A. These two proteins act synergistically to prevent nuclear occupancy of nuclear factor of activated T cells, namely cytoplasmic, calcineurin-dependent 1 (*NFATc*) transcription factors, which are regulators of vertebrate development. Recently, Baek *et al*. showed that the increase in dosage of these two proteins is sufficient to confer significant suppression of tumour growth in Ts65Dn mice [11], and that such resistance is a consequence of a deficit in tumour angiogenesis arising from suppression of the calcineurin pathway [12]. Overexpression of a number of HSA21 genes, including *Dyrk1a*, *Synj1 *and *Sim2*, results in learning and memory defects in mouse models, suggesting that trisomy of these genes may contribute to learning disability in DS patients [13-15].

Many phenotypic features of DS are determined very early in development, when the tissue specification is not completely established [3]. Early postnatal development of both human patients and DS mouse models showed the reduced capability of neuronal precursor cells to correctly generate fully differentiated neurons [16], contributing to the specific cognitive and developmental deficits seen in individuals with DS [17]. Canzonetta *et al. *[18] showed that *DYRK1A-REST *perturbation has the potential to significantly contribute to the development of defects in neuron number and altered morphology in DS. The premature reduction in REST levels could skew cell-fate decisions to give rise to a relative depletion in the number of neuronal progenitors.

The exact nature of these events and the role played by increased dosage of individual HSA21 genes remain unknown. To contribute to answering these questions, we have established a cell bank consisting of mouse embryonic stem (mES) cell clones capable of the inducible overexpression of each one of 32 selected genes, 29 murine orthologs of HSA21 genes and 3 HSA21 coding sequences, under the control of the tetracycline-response element (tetO). These genes include thirteen transcription factors, one transcriptional activator, six protein kinases and twelve proteins with diverse molecular functions. By transcriptome and proteome analysis, we determined that these clones, which are able to differentiate in different cell lineages, can be used to unveil the pathways in which these genes are involved. We believe that this resource represents a valuable tool to analyse the genetic pathways perturbed by the dosage imbalance of HSA21 genes.

## Results

### Validation of an inducible/exchangeable system for generation of transgenic mES cells

In order to generate a library of mES transgenic lines of selected HSA21 genes, we used the ROSA-TET system. This integrates the inducible expression of the Tet-off system, the endogenous and ubiquitous expression from the *ROSA26 *locus, and the convenience of transgene exchange provided by the recombination-mediated cassette exchange (RMCE) system [19]. Briefly, coding sequences are cloned into an expression vector, driven by an inducible promoter (Tet-off), which can be easily integrated into the *ROSA26 *locus through a cassette exchange reaction.

Understanding the expression kinetics of the system was essential to standardizing the generation of the mES library encoding the HSA21 genes. Towards this goal, we first tested the system by introducing the *luciferase *(*Luc*) gene, cloned into an exchange vector. This enabled accurate quantification of cassette exchange and gene inducibility, at both the RNA and protein level. To this end, we prepared an exchange vector (pPTHC-Luc), which was introduced into the EBRTcH3 ES cell line (EB3), carrying a yellow fluorescent protein (*YFP*) gene integrated in the *ROSA26 *locus. After the RMCE procedure, positive exchanged clones were identified by PCR (Additional file 1a) and their inducibility verified using both reporter genes. Quantitative PCR (q-PCR) analysis of *Luc *expression showed that the system was activated upon the removal of Tetracycline (Tc) from the medium. In the presence of Tc (0 hours; see Materials and methods), *Luc *mRNA was undetectable, indicating that the background expression level was almost zero, whereas a strong signal was detected 15 hours after Tc withdrawal, and still sustained over a time window of 48 hours (Additional file 1b). We then compared the mRNA level with the enzymatic activity of the protein Luc. To this end, we prepared the protein extracts of the *Luc*-inducible mES clones at the same time points to quantify luminescence. In agreement with the mRNA data, the enzymatic activity was undetectable in the presence of Tc, whereas a strong signal was measurable 15 hours after Tc withdrawal, indicating a correct induction of *Luc *translation (Additional file 1b).

We next verified the expression of the *YFP *reporter gene, which is separated from the *Luc *gene in the recombinant locus by an *IRES *sequence, and we detected a comparable level of *YFP *expression and protein accumulation following induction. The maximal expression of the reporter gene was observed 24 hours after complete removal of Tc from the medium (Additional file 1c).

The level of gene expression can be regulated by adjusting the concentration of Tc in the culture media. Using a ten-fold dilution of Tc, negligible expression of the *YFP *gene was seen (Additional file 1d), while further dilution of Tc revealed increasing expression levels of *YFP*.

We then verified the growth properties of this mES line (EB3) compared to the parental line (E14) (data not shown) and the ability of these cells to differentiate along the three germ layers. The EB3 cells displayed the expected transcript down-regulation of the pluripotency gene *Oct3/4*, and a marked increase of the mesoderm-specific marker *Brachyury*, of the ectoderm-specific marker *Gfap *and the endoderm-specific marker *Afp *during mES differentiation (Additional file 1e).

Collectively these data suggest that, in mES cells, this system allows the efficient and long-term overexpression of the transgene in a dose- and time-dependent manner. It is therefore suitable for systematic expression of HSA21 cDNAs.

### Cell bank: the HSA21 gene collection in mES cells

HSA21 is syntenic to three different mouse chromosomal regions located on chromosomes 10, 16 and 17. These three regions contain 175 murine orthologs of protein coding HSA21 genes according to [20].

For the generation of mES clones with inducible overexpression, we selected a subset of 32 genes, 29 of which are murine orthologs of HSA21 genes, and 3 of which are human coding sequences (see also Materials and methods). The 32 genes encode 13 transcription factors (*Aire*, *Bach1*, *Erg*, *Ets2*, *Gabpa*, *Nrip1*, *Olig1*, *Olig2*, *Pknox1*, *Runx1*, *Sim2*, *ZFP295*, *1810007M14Rik*), a single transcriptional activator (*Dscr1-Rcan1*), 6 protein kinases (*DYRK1A*, *SNF1LK*, *Hunk*, *Pdxk*, *Pfkl*, *Ripk4*) and 12 proteins with diverse molecular functions (*Atp5j*, *Atp5o*, *Cct8*, *Cstb*, *Dnmt3l*, *Gart*, *Dscr2-Psmg1*, *Morc3*, *Mrpl39*, *Pttg1ip*, *Rrp1*, *Sod1*) (refer to Additional file 2 for more general information about these genes).

**Figure 1:**
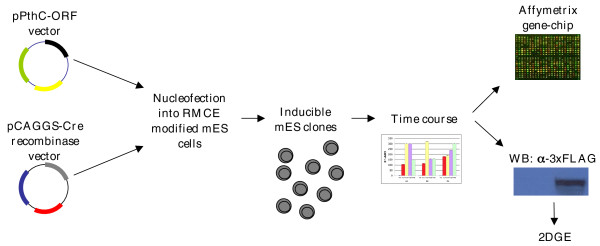
**Schematic representation of the experimental strategy used**. A set of 32 genes, 29 murine orthologs of HSA21 genes and 3 human coding sequences, were cloned into the pPthC vector [19] and nucleofected along with a pCAGGS-Cre recombinase vector [41] into EBRTcH3 (EB3) cells. Puromycin-resistant clones were isolated and grown in medium deprived of tetracycline for varying periods of time to perform a time course of induction. The inducibility of selected clones was evaluated by q-PCR. Global transcriptome and proteome analysis was performed by hybridization onto an Affymetrix gene chip and by large-gel two-dimensional gel electrophoresis (2DGE), respectively, to delineate the consequences of gene dosage imbalance on a single gene basis. WB, western blot.

For a subset of the selected genes, there is evidence for the presence of different alternatively spliced isoforms that may differ in their coding sequence. In such cases, we overexpressed the longest annotated coding sequence. For one transcription factor (*ZFP295*) and two protein kinases (*DYRK1A*, *SNF1LK*), we used the human coding sequences (see also Materials and methods). A schematic representation of our experimental strategy is shown in Figure 1.

In order to generate the mES library overexpressing a subset of HSA21 ORFs, we employed the ROSA-TET system, as previously described. The expression construct contained the 3xFLAG epitope at the carboxyl terminus, thus enabling monitoring of transgene protein product. We constructed exchange vectors carrying each of the 32 ORFs and then nucleofected the plasmids into the RMCE recipient mES lines to generate stable clones (see Materials and methods). For each gene, an average of 20 drug-resistant clones were picked, amplified and characterized by PCR analysis.

Three positive clones for each gene were grown in medium deprived of Tc for varying periods of time to verify the sensitivity of each mES line to Tc by performing a time course experiment to identify the capacity of each transgene to be overexpressed. In total we analyzed 96 clones (3 biological replicates for 32 transgenes). As shown in Additional file 3, we performed a time course experiment, at four different time points (17, 24, 39 and 48 hours), for 16 genes: 3 transcription factors (*Aire*, *Sim2 *and *ZFP295*), a protein kinase gene (*Hunk*) and for all the 12 genes encoding proteins with diverse molecular functions (*Atp5j*, *Atp5o*, *Cct8*, *Cstb*, *Dnmt3l*, *Gart*, *Dscr2-Psmg1*, *Morc3*, *Mrpl39*, *Pttg1ip*, *Rrp1*, *Sod1*). Since the majority of the genes analyzed showed the highest level of induction after 24 hours of Tc deprivation, we decided to test the inducibility of the remaining clones at one time point only. As shown in Additional file 3, we tested 12 clones at one time point: the transcription factors *Bach1*, *Erg*, *Ets2*, *Gabpa*, *Nrip1*, *Olig1*, *Pknox1*, *Runx1*, *1810007M14Rik*), the transcriptional activator *Dscr1-Rcan1 *and the protein kinases *Pdxk *and *Pfkl*. Finally, one transcription factor (*Olig2*) and three protein kinases (*DYRK1A*, *SNF1LK *and *Ripk4*) were tested at three different time points (17, 24, and 39 hours). As a control, total RNA extracted from uninduced clones (in the presence of Tc, 0 hours) was used.

**Figure 2:**
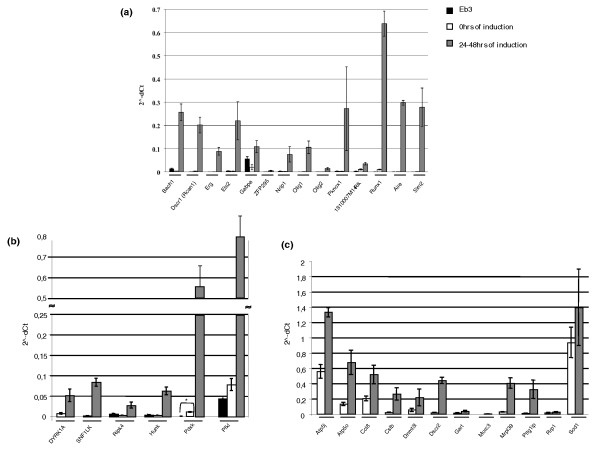
**Average induction of the 32 inducible clones by q-PCR**. Baseline expression (0 hours of induction - white bars), following induction of transgene (after 24 to 48 hours of growth in medium deprived of Tc - gray bars), and relative expression in the parental cell line (EB3 - black bars). **(a) **The 13 transcription factors and the single transcriptional activator (*Dscr1-Rcan1*); **(b) **the 6 kinases; **(c) **the other 12 genes with diverse molecular functions. Asterisks indicate statistically significant expression changes (*t*-test with false discovery rate <0.05). The errors bars are calculated on the biological triplicates.

Figure 2 shows the average induction, evaluated by q-PCR (Additional file 4) and expressed as relative expression (2^-dCt^), of the 13 transcription factors together with the single transcriptional activator (Figure 2a), the 6 kinases (Figure 2b), and the 12 genes with diverse molecular functions (Figure 2c). For the 13 transcription factors and the transcriptional activator (Figure 2a) and the 6 kinases (Figure 2b) we assessed the potential leakiness of the inducible system in our mES clones. To this aim, we compared the basal expression level of each gene in the parental cell line (EB3) with the expression level in the corresponding transgenic inducible clones (in the biological replicates) grown in the presence of Tc in the medium (0 hours of induction). Results are shown in Figure 2a,b and in Additional file 5. We verified that only in the case of *Pdxk *is there a statistically significant (corrected *P*-value false discovery rate (FDR) = 0.04), albeit mild, leakiness.

We then checked for the proper ploidy of the clones following extensive passages in culture. To this end, we performed a karyotype assay (Materials and methods) on parental ES cells (EB3) and on 20 different inducible clones of our mES cell bank (representing the 7 effective and the 13 silent genes). All these clones turned out to display a normal karyotype (40 chromosomes).

### Transcriptome analysis of mES cell lines

In order to identify the effects of the overexpression of a single gene on the mES transcriptome, we performed Affymetrix Gene-Chip (Mouse 430_2) hybridization experiments for a set of clones overexpressing 20 of the 32 genes (that is, the transcription factors and protein kinases). As we used biological triplicate clones for each gene, this analysis was performed on a total of 60 clones. Total RNA was extracted from each clone at the time-point of maximal expression (Additional file 3), following Tc removal from the medium (Materials and methods). As a control, total RNA extracted from un-induced clones was also used. This procedure resulted in a total of 120 hybridization experiments (the whole set of results is available in the Gene Expression Omnibus database [GEO:GSE19836]).

In order to identify downstream transcriptional effects of the 20 overexpressed genes, microarray data were analyzed to detect differentially expressed genes (that is, in induced versus non-induced cells). We first normalized together both induced and non-induced hybridizations, and then detected differentially expressed genes using a Bayesian *t*-test method (Cyber-t) followed by FDR correction (threshold FDR < 5%). The overexpression of 7 out of 20 genes perturbed the mES transcriptome in a statistically significant manner: we will refer to these seven genes as the 'effective' genes, as opposed to the other 13, 'silent' genes. In Additional files 6, 7, 8, 9, 10, 11 and 12, we report complete lists of differentially expressed genes following the overexpression of each of the effective genes.

The effective genes consisted of six transcription factors (*Runx1*, *Erg*, *Nrip1*, *Sim2*, *Olig2 *and *Aire*) and one kinase (*Pdxk*). Differential expression was also validated by q-PCR, selecting a subset of the most up-regulated and down-regulated genes (Additional file 13). In order to identify possible biological processes in which the effective genes are involved, we performed a Gene Ontology (GO) enrichment analysis on the lists of differentially expressed genes. We used the DAVID online tool [21-23], restricting the output to biological process terms of levels 4 and 5, with a significance threshold of FDR < 5% and fold enrichment ≥ 1.5%. In Table 1 we report the subsets of significant GO terms for six (*Runx1*, *Erg*, *Nrip1*, *Olig2*, *Pdxk *and *Aire***) **out of the seven effective genes that were in agreement with their known function, as suggested by evidence in the literature. A complete list of all significantly enriched GO terms for the seven effective genes is reported in Additional file 14.

**Table 1:** Gene Ontology enrichment analysis for six out of seven effective genes whose overexpression perturbed the mES transcriptome in a statistically significant manner

Gene	Gene Ontology term	FDR	Fold enrichment	Reference
*Runx1*	Negative regulation of progression through cell cycle	2.8	1.5	[60,61]
	Positive regulation of cell proliferation	0.5	1.5	[60,61]
	Vasculature development	0.1	1.5	[62,63]
	Blood vessel development	0.2	1.5	[62,63]
	Blood vessel morphogenesis	0.1	1.6	[62,63]
	Angiogenesis	0.1	1.6	[62,63]
	Regulation of myeloid cell differentiation	0.8	2.1	[63]
	Skeletal development	0.2	1.5	[64]
	Skeletal morphogenesis	1.7	2.5	[64]
	Regulation of cell differentiation	0.0	1.7	[65]
				
*Erg*	Anatomical structure formation	0.2	1.5	[66]
	Angiogenesis	0.0	1.6	[67,68]
	Regulation of cell differentiation	0.8	1.5	[67,68]
	Cell growth	2.3	1.5	[67,68]
	Regulation of cell migration	1.3	1.8	[67,68]
	Negative regulation of transcription, DNA-dependent	0.5	1.5	[68]
				
*Nrip1*	Muscle cell differentiation	2.0	4.3	[69]
	Nervous system development	0,1	2.0	[70]
	Negative regulation of transcription, DNA-dependent	3.6	2.6	[71]
				
*Olig2*	Organ morphogenesis	0.5	1.6	[72,73]
	Placenta development	1.3	4.3	[74]
	Negative regulation of progression through cell cycle	3.6	2.1	[75]
				
*Pdxk*	Cellular macromolecule catabolic process	2.0	2.7	[76,77]
	Cellular carbohydrate metabolic process	0.0	4.1	[76,77]
	Amino acid biosynthetic process	0.6	7.3	[78]
				
*Aire*	Cell morphogenesis	0.0	1.6	[79]
	Regulation of progression through cell cycle	0.0	1.7	[79]
	Regulation of cell differentiation	0.4	1.9	[79]
	Cell migration	4.0	1.5	[80]

Perturbation of the mES transcriptome was as assessed by microarray analysis. GO analysis was performed on the list of differentially expressed genes using the DAVID tool, restricting the output to biological process terms of levels 4 and 5, with a significance threshold FDR < 5% and fold enrichment ≥ 1.5%. Supporting references confirming GO analysis are reported in the 'References' column.

### High basal expression level of HSA21 genes in mES cells correlates with a lack of transcriptional response following their overexpression

A possible explanation for the lack of a strong transcriptional response following the overexpression of the silent genes could be that they failed in their disturbance of mES cell homeostasis because of a rapid degradation of the synthesized protein. To test this hypothesis, we grew three clones for each effective and for each silent gene in medium deprived of Tc for 24 hours or 48 hours to induce the expression of their protein products. Our expression construct contains the epitope 3xFLAG at the carboxyl terminus of each gene, which allows the detection of the expression of each corresponding protein product by western blotting. A significant protein band was visible on the western blot for all the genes tested, thus leading us to reject this hypothesis.

**Figure 3:**
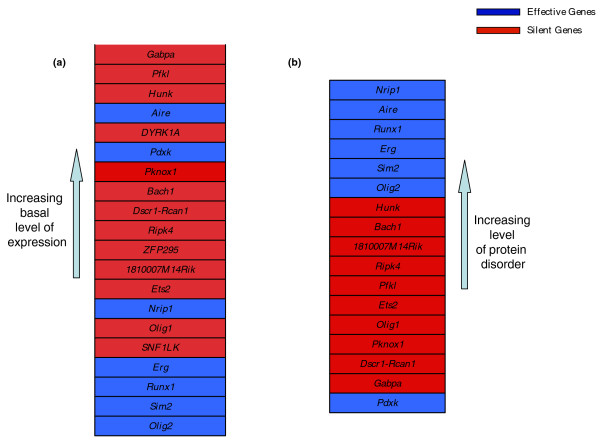
**The basal expression level and dosage sensitivity of HSA21 genes in mES cells**. The effective genes are highlighted in blue, and the silent genes in red. **(a) **Selected HSA21 genes sorted according to their average expression level in mES cells, from the most (gene rank = 1) to the least expressed. **(b) **Selected HSA21 genes sorted according to the total length of the 'disordered' region of the encoded protein (measured with the GlobPlot tool).

An alternative hypothesis is that these genes have a high basal expression level in mES cells, and therefore their overexpression will result in only a weak effect on the mES transcriptome. In order to verify this hypothesis, we estimated, using all the 120 microarray experiments, the average expression level of each gene, and its corresponding standard deviation. We reasoned that, due to the large number of arrays, the average expression level for each gene can be considered as a reliable estimate of its basal level of expression in mES cell. In Additional file 15 and in Figure 3a we rank HSA21 genes according to their average expression level, from the most to the least expressed. We highlight in red the 13 silent genes and in blue the 7 effective genes. It is evident that the effective genes show a different distribution from the silent genes: the silent genes tend to be highly endogenously expressed in mES cells, whereas the effective genes tend to be expressed at lower levels. A gene set enrichment analysis (GSEA) [24] was performed to compute the significance of this different distribution (see Materials and methods); this produced a significant enrichment score of 0.402 (FDR q-value = 0). This observation supports the hypothesis that the lack of a strong transcriptional response following the overexpression of some of the HSA21 genes is due to a high basal expression level of these genes.

### Dosage sensitivity of HSA21 genes in mES cells

We further investigated the cause of the lack of a strong transcriptional response in the silent gene set in order to predict which genes are most sensitive to dosage. A recent study has shown a strong correlation between the sensitivity to increased dosage of a gene and the degree of a certain property of the encoded protein, called intrinsic disorder [25]. The protein disorder is defined as the total number of amino acids included in unstructured regions of the protein. These regions usually contain short sequence motifs (such as localization signals, or nuclear import/export signal), leading to a higher sensitivity to protein dosage [25]. We thus measured protein disorder for both silent and effective genes, excluding the clones in which the human coding sequences were introduced (*ZFP295*, *DYRK1A*, *SNF1LK*) from this analysis because of the possible confounding effect represented by their non-murine origin. In Figure 3b, the silent and effective genes are clearly segregated according to their average level of protein disorder (separation of means verified with *t*-test, *P*-value = 0.043). The segregation is almost perfect (with a threshold value for the protein disorder equal to 180) with the only exception being *Pdxk*, which is an effective gene despite its low disorder value of 26. We attribute this anomaly to the fact that *Pdxk *is a kinase (the only one in the effective gene list), and its function might place it at the crossroads of a number of crucial pathways.

### Comparison with the transcriptional response of the transchromosomic Tc1 mouse line

To demonstrate the potential value of our cell bank in elucidating the transcriptional changes underlying trisomy 21, we compared the output of our overexpression experiments with the transcriptional profile obtained on the 'transchromosomic' Tc1 mouse line [26]. The Tc1 ES cells carry an extra copy of HSA21 and they represent a reference model of trisomy 21 for which publicly accessible transcriptional data in ES cells are available, enabling a direct comparison with our cell bank overexpression experiments. As reported in [26] the Tc1 line is missing some portions of HSA21; however, we verified that all of our 'effective' genes were included, based on the published chromosome map. We have verified that the seven 'effective' genes are all included in the extra chromosome present in the Tc1 line.

**Figure 4:**
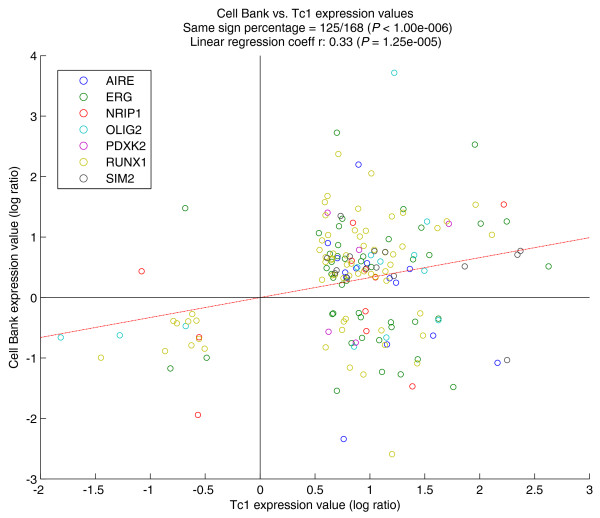
**Comparison of differentially expressed genes following single gene over-expression in our cell bank mouse ES cell lines versus transchromosomic Tc1 mouse ES cell lines**. The colors indicate the overexpression experiment in which the expression value was found to be significant; for genes whose expression was significant in more than one overexpression experiment, only the one with the largest absolute value was considered. A total of 168 points are in the graph, of which 125 fall in same-sign quadrants. The regression line was forced to pass through the origin in order to highlight the general trend with respect to zero.

Figure 4 shows a scatter plot of the differential expression values following the overexpression of the cell bank genes compared to the differential expression values of genes in the Tc1 ES cell line. We included in this analysis all of the genes that were significantly differentially expressed in both Tc1 and at least one of the seven 'effective' cell bank overexpression experiments. Of all the points in the graph, the ones with the same sign coordinates (both positive or both negative *x*, *y *values) represent genes whose transcriptional up- or down-regulation, observed in at least one of the overexpression experiments, is concordant with the transcriptional changes in the Tc1 cells versus control. A statistically significant 125 out of a total of 168 points fall in same-sign quadrants (*P *< 1e-6). We also separately compared each of the seven overexpression experiments with Tc1 ES cells (Additional file 16); five out of seven effective genes had a statistically significant number of genes with same sign fold-change as in Tc1 cells (*Runx1*, *Erg*, *Nrip1*, *Sim2*, *Aire*; Additional file 17). These observations suggest that the transcriptional features of trisomic Tc1 cells can be partially explained as an additive effect of single gene overexpression, thus highlighting the usefulness of our cell bank in elucidating DS.

### Refined analysis of the transcriptional response to the overexpression of silent genes

We verified the possibility to also detect differentially expressed genes in those experiments involving the overexpression of silent genes by using a more sensitive statistical method than the standard *t*-test approach. The method we selected was Bayesian analysis of variance for microarrays [27-29], a Bayesian spike and slab hierarchical model, as implemented in the BAMarray tool (BAMarray 3.0) [27]. Using this procedure, transcriptional changes were detected in all silent gene overexpression experiments, despite the low fold change of differentially expressed genes, which therefore could include more false positives than the standard *t*-test.

In order to identify possible biological processes in which the silent genes are involved, we performed the GO enrichment analysis on the list of newly identified differentially expressed genes. In Additional file 18 we report all the significantly enriched GO terms for 11 out of 13 silent genes (for the remaining two silent genes, *Ets2 *and *1810007M14Rik*, no significant GO terms were found). In Additional file 19 we report the subset of significant GO terms for 5 (*Bach1*, *Dscr1-Rcan1*, *DYRK1A*, *Gabpa *and *SNF1LK*) out of 13 silent genes, which are in agreement with the known functions of these genes, as determined by evaluation of the literature.

### Proteome analysis in mES cells overexpressing the *Runx1 *gene

In order to assess whether the overexpression of single genes in mES causes changes in the proteome comparable to those detected by microarray hybridization experiments, we performed a full proteomic analysis following overexpression of the transcription factor *Runx1*. This involved high resolution large-gel two-dimensional electrophoresis (2DGE) followed by protein identification performed with database-assisted mass spectrometry. The peak of response at the proteomic level, as assessed by a pilot 2DGE assay on a single Runx1-overexpressing clone (E6), was observed at 48 hours after depletion of Tc, rather than at 24 hours as observed at the transcriptome level for this gene, suggesting a delayed effect due to the fact that protein synthesis occurs subsequent to that of mRNA. We therefore decided to perform the analysis on two Runx1-overexpressing clones (E6 and E7; Additional file 3) by comparing the 2DGE results obtained from the non-induced state (that is, cells grown in the presence of Tc) with those derived from cells grown in a medium deprived of Tc for 48 hours (in other words, cells overexpressing the protein Runx1). For each of the two Runx1-overexpressing clones, three technical replicates were then generated (see Materials and methods). Our 2DGE image data have now been submitted to the World-2DPAGE Repository of the ExPASy Proteomics Server [2DPAGE:0021] [30] for public access [31].

The induction of Runx1 changes the expression of at least 54 proteins (Additional file 20). Of these, 24 were consistently down-regulated while 30 were up-regulated after 48 hours of induction of the protein Runx1. The effect of Runx1 overexpression on the proteome was compared with the effect on the transcriptome, as detected by microarray.

In Table 2, we compare changes in protein levels 48 hours after induction of Runx1 to changes in mRNA levels 24 hours after induction of *Runx1*. There is a substantial overlap (15 out of 17 affected gene/protein pairs showing similar trends of expression variations) between microarray data and data obtained from the 2DGE assay: 6 out of 24 down-regulated proteins and 9 out of 31 up-regulated proteins displayed similar trends in the corresponding transcripts by microarray analysis. Only two gene/protein pairs, *apoE *and *Sept1*, showed opposite behavior in the protein versus microarray assays. Both proteins showed up-regulation, while their mRNA levels showed down-regulation, which suggests that the mRNAs of these two genes might be unstable, leading to longer half-lives of the proteins.

**Table 2:** Correlation between differential protein expression by 2DGE (protein ratio) and differential gene expression by microarray (mRNA ratio)

Spot ID*	Gene	Protein name	Protein ratio 48 h/0 h*	mRNA ratio 24 h/0 h
3214	*Uchl1*	Ubiquitin carboxy-terminal hydrolase L1	0.58	0.63
662	*Dppa4*	Developmental pluripotency associated 4 isoform 1	0.65	0.75
3549	*Igf2bp2*	Insulin-like growth factor 2 mRNA-binding protein 2 (IGF-II mRNA-binding protein 2) (IMP-2)	0.65	1.39
2836	*Sipa1l1*	Signal-induced proliferation-associated 1-like protein 1	0.74	1.54
425	*Lap3*	Cytosol aminopeptidase (Leucine aminopeptidase) (LAP) (Leucyl aminopeptidase) (Leucine aminopeptidase 3) (Proline aminopeptidase)(Prolyl aminopeptidase)	0.76	0.72
2512	*Hspd1*	60 kDa heat shock protein, mitochondrial precursor (Hsp60) (60 kDa chaperonin) (CPN60) (Heat shock protein 60) (HSP-60) (Mitochondrial matrix protein P1) (HSP-65)	0.78	0.75
3410	*Eif1a*	Eukaryotic translation initiation factor 1A, Y-linked	0.8	0.76
403	*Pkm2*	Pyruvate kinase isozyme M2	1.26	3.05
935	*Bdh1*	D-beta-hydroxybutyrate dehydrogenase, mitochondrial precursor	1.31	1.53
645	*Serpinh1*	Serine (or cysteine) proteinase inhibitor, clade H, member 1	1.44	1.36
2920	*Ldhb*	Lactate dehydrogenase 2, B chain	1.46	0.64
3562	*Cotl1*	Coactosin-like 1	1.62	1.39
3653	*S100a11*	S100 calcium binding protein A11 (calizzarin)	1.89	1.18
3144	*Sept1*	Septin 1	2.28	0.71
1134	*Gsto1*	Glutathione S-transferase omega 1	2.77	3.73
3588	*Fabp3*	Fatty acid binding protein 3, muscle and heart	3.05	2.2
3078	*Apoe*	Apolipoprotein E	3.28	0.8

Only two gene/protein pairs, apoE and Sept1, showed bifurcated regulation in protein and microarray assays.

## Discussion

The mechanisms by which the presence of three copies of HSA21 result in the complex and variable phenotype observed in DS patients are a major focus of research. Recently, it has been shown that only some genes are likely to be dosage-sensitive [7,8]. There is a need for further experimental studies assessing the variability among samples, tissues and developmental stages [32]. To overcome the problem of transcriptome and proteome variability due to differences in the human population, mouse inter-strain variability, and tissue sampling and processing, we generated a cell bank of cultured mES cells. For years, the importance of mES cells to biology and medicine has been attributed both to their ability to proliferate for an indefinite period of time while still retaining their normal karyotype following extensive passaging in culture [33], and to their suitability as a model system for studying, *in vitro*, the molecular mechanisms that regulate lineage specification and differentiation [34].

Our work has produced the first resource for systematic overexpression of single HSA21 genes in mES cells using an inducible system. Our cell bank can be used to understand how much, and in what way, the dosage imbalance of specific HSA21 genes perturb the molecular pathways in ES cells, and eventually in DS. This strategy has the advantage of dramatically simplifying the investigation of single gene dosage effects, with the intrinsic limitation given by the impossibility to study two or more gene interactions. In addition to providing a mES cell bank for the overexpression of 32 distinct genes, we also developed a standardized approach for the generation of mES clones to be added to this cell bank. This opens the possibility of using this system to study other aneuploidy disorders in which the gene dosage imbalance seems to be the main cause of the disease, including the micro-aneuploidies recently described by assays based on comparative genomic hybridization arrays [35]. We are aware that the massive overexpression of the transgene may not fully reproduce the downstream effects on the cell transcriptome caused by the 3:2 dosage imbalance of trisomy 21 [36]. However, we reasoned that most of the downstream transcriptome effects may be shared by both experimental conditions, and at least some of the subtle transcriptome alterations present in trisomy 21 may become much more evident by massive overexpression of trisomy 21 genes, thus facilitating their identification. Therefore, we decided not to induce a 3:2 overexpression for any of the analyzed genes. Moreover, Nishiyama *et al*. [37] have recently shown using a similar tet-inducible system for massive overexpression of transcription factor genes in mouse ES cells that it is indeed possible to identify their physiological function from transcriptome analysis. We have also shown that some effects may be shared by both experimental conditions (massive versus 3:2 overexpression), since we observed concordant results by comparing single gene overexpression and trisomic Tc1 mES cell lines (Figure 4; Additional file 17). We suggest that some of the transcriptional features of trisomic Tc1 cells are partly due to an additive effect of single gene overexpression. Although our data are not sufficient to prove that these responses are additive, in a genetic sense of the word their extent and the significance of their sign concordance is certainly worth future investigation.

Full gene expression profiling for all the mES clones that overexpress 29 murine coding sequences and 3 HSA21 genes (refer to Additional file 2 for details) are provided, thus facilitating the search for new HSA21 gene targets and the elucidation of the transcriptional network underlying gene function.

Only a subset of 7 out of 20 genes in our overexpression study yielded a strong perturbation of the mES transcriptome, at least via microarray analysis. More subtle transcriptional changes might be detected when using more sensitive techniques such as RNA-seq technology [38]. We excluded the possible rapid degradation of the synthesized silent protein as an explanation of the inability of these overexpressed genes to produce significant changes in the mES transcriptome. We hypothesized an inverse correlation between transcriptional response and the basal expression level and the protein disorder of the overexpressed genes (Figure 3). Our observation can be useful to predict those genes with a higher probability of displaying dosage-sensitivity. However, we cannot exclude the possibility that the absence of a transcriptional response to the overexpression of some transcription factors and protein kinase genes reflects, for example, the absence of the proper protein partners in undifferentiated cells. In support of this hypothesis, none of the transgenic mouse lines generated as an *in vivo *model to study the effect of the overexpression of some HSA21 genes have so far been found to determine embryonic lethality, whereas they showed a clear phenotype in differentiated tissues (that is, TG-DYRK1a in brain, TG-DSCR1/Rcan1 in heart/vasculogenesis [39,40]). Therefore, future studies will be necessary to prove whether defects, which can take place early in development (such as the elevated risk of miscarriage of trisomic fetus), are due to the overexpression of effective genes.

We also quantified the effect of single gene overexpression on the proteome. Specifically, we performed a proteomic analysis on one of the overexpressing clones (*Runx1*) by the high-resolution 2DGE method. The comparison of the effect on the proteome with the effect on the transcriptome showed a strong correlation, with 15 out of 17 affected gene/protein pairs showing similar trends of expression variations (Table 2). However, two proteins (apolipoprotein E and septin 1) showed bifurcated regulation in protein and microarray assays. Both proteins show up-regulation, while their mRNA levels show down-regulation. This could suggest that the mRNAs of these two genes are unstable, leading to longer half-lives of the proteins.

## Conclusions

We have developed a mES cell bank for inducible expression of a set of murine orthologs of HSA21 genes. This resource represents an invaluable tool for future studies involving their differentiation into cardiomyocytes, and myeloid and neuronal lineages, which represent cell types/tissues affected by DS. The detection of early changes, at the level of undifferentiated mES cells, may be instrumental to a better understanding of some phenotypic features of DS, and possibly of other human aneuploidies.

## Materials and methods

### Cell culture

The cell line EBRTcH3 (EB3) was obtained from the laboratory of Dr Hitoshi Niwa and have been previously described in [19].

mES cells were grown in mES media + leukemia inhibitory factor (LIF) (DMEM high glucose (Invitrogen Ltd, Paisley, UK, catalog no. 11995-065) supplemented with 15% fetal bovine serum defined (HyClone, Thermo Scientific, Logan, UT, USA, catalog no. SH30070.03), 0.1 mM nonessential amino acids (Gibco-Brl, Invitrogen Ltd, Paisley, UK, catalog no. 11140-050), 0.1 mM 2-mercaptoethanol (Sigma-Aldrich, St. Louis, MO, USA, catalog no. M6250), and 1,000 U/ml ESGRO-LIF (Millipore, Billerica, MA, USA, catalog no. ESG1107)) at 37°C in an atmosphere of 5% CO_2_. All stable cell lines derived from EB3 were grown in mES media + LIF supplemented with 1 μg/ml Tc (Sigma, catalog no. T7660). For antibiotic selection of RMCE lines, mES + LIF + Tc supplemented with 1.5 μg/ml of puromycin (Sigma, catalog no. P9620) was used. In the case of two of the mES inducible clones (*ZFP295*, *Hunk*), these were grown in mES + LIF + Tc supplemented with 7.5 μg/ml puromycin to decrease the variation among the biological replicates of clones.

mES cells were trypsinized (in Trypsin-EDTA solution 10×, Sigma, catalog no. T4174) and plated 1 day before the nucleofection on 0.1% gelatin (Gelatin Type I from porcine skin, Sigma) coated 100-mm dishes (Nunc Gmbh & Co., Langenselbold, Germany, catalog no. 150350) in mES media + LIF supplemented with Tc. For nucleofection 2 × 10^6 ^cells were counted for each sample. Plasmids were prepared using Qiagen plasmid Midi kit (Qiagen spa, Milano, Italy, catalog no. 12145): 5 to 6 μg of pPthC vector containing each ORF [19] were incubated with 3 μg of pCAGGS-Cre vector [41] and 100 μl of Mouse ES Cell Nucleofector Kit (Amaxa, Lonza Cologne, Germany, catalog no. VPH-1001) was added to the plasmid mix. The nucleofection program used was the A30 program. Cells were then incubated for 10 to 15 minutes at room temperature in the presence of complete medium and plated. The day after the nucleofection, cells were washed twice with PBS (Dulbecco Phosphate buffered Saline 1×, Gibco, catalog no. 14190), and switched to selection media (mES + LIF + Tc + 1.5 μg/ml puromycin). The colonies were grown for approximately 7 to 8 days before they were individually trypsinized and transferred to 96-well U-bottom plates (Nunc, catalog no. 163320). Trypsinized cells were neutralized with mES media + LIF, vigorously pipetted, and then each clone was equally distributed among two gelatin-coated 48-well plates (Nunc, catalog no. 150687), the former with selection media and the latter with mES + LIF + 150 μg/ml hygromicin (Hygromycin B in PBS, Invitrogen, catalog no. 10687-010). When confluent, the clones resistant to selection media and completely dead in parallel in mES media + LIF + hygromicin were isolated, replicated in 12-well plates (Nunc, catalog no. 150628) and when confluent replicated in 6-well plates (Nunc, catalog no. 140675) to extract the genomic DNA using standard conditions.

The positive clones were identified by PCR using standard conditions using the following primer pair: 5'-GCATCAAGTCGCTAAAGAAGAAAG-3' and 5'-GAGTGCTGGGGCGTCGGTTTCC-3'. All positive clones analyzed were frozen at -135°C using standard conditions.

In compliance with our policy of distribution of published reagents, all the mES clones generated within this project are available for distribution to academic research centers upon request.

### Cloning strategy

The exchange vector pPthC-*Oct-3/4 *was obtained from the laboratory of Dr Hitoshi Niwa and has been previously described in [19].

For the cloning of each gene we decided to use only the coding sequence, from the ATG to the stop codon, without the 5' and 3' untranslated regions. For 29 ORFs, we cloned the murine coding sequence, while for 1 transcription factor (*ZFP295*) and 2 protein kinases (*DYRK1A*; *SNF1LK*) we used the human coding sequence (see Additional file 2 for more general information about these genes). For a subset of the selected genes there is evidence for the presence of different alternatively spliced isoforms that may differ in their coding sequence. In this case we decided to clone the longest annotated coding sequence.

The exchange vector was modified, in the region between XhoI and NotI restriction sites, by adding a multiple-cloning site that contains sequences recognized by three restriction enzymes (I-SceI, AscI and PacI) and by adding the epitope 3 × FLAG. Two double-stranded oligonucleotides, containing 3 × Flag sequence, with the sequences recognized by PacI and NotI at the 5' and 3' ends, respectively, were designed. These oligonucleotides were then inserted into the exchange vector, and digested by PacI-NotI. The epitope 3 × FLAG was designed to be in frame with the stop codon of each ORF.

The plasmids containing the cDNAs of *Gabpa*, *Olig1 *and *Dscr1 *were obtained from Biotech Custom Services Primm srl (Milano, Italy); the plasmid containing the cDNA of *Olig2 *was obtained from the laboratory of Dr Yaspo; the plasmid containing the cDNA of *Runx1 *was obtained from the laboratory of Dr Groner; the plasmid containing the cDNA of *Sim2 *was obtained from the laboratory of Dr Whitelaw. The cDNAs of *Aire*, *1810007M14Rik*, *Erg *and *Hunk *were obtained by retro-transcription with SuperScript III Reverse transcriptase (Invitrogen, catalog no. 18080-044) from total RNA extract of embryonic stem cells. All other plasmids were purchased from ImaGENES (formerly RZPD, Berlin, Germany).

The cDNAs were amplified using the plasmids as templates by PCR in standard conditions. The forward and reverse primers used to amplify the cDNAs were designed to include in the sequence the restriction sites recognized by the enzymes AscI and PacI at the 5' and 3' ends, respectively.

Primer pair sequences used for the cloning are available in Additional file 21. In the case of *Cstb*, the primers introduce the sequence recognized by PacI at both ends of the amplified product while, in the case of *Runx1*, the primers introduce the restriction sites of XhoI and NotI at the 5' and 3' ends, respectively. After digestion with the specific restriction enzymes, the cDNA fragments were cloned into pTOPO-bluntII (Invitrogen, catalog no. K2875J10). The pTOPO-bluntII containing the cDNAs was then cleaved by AscI-PacI or only by PacI (for *Cstb*) or by XhoI-NotI (for *Runx1*). The fragments obtained by digestion were separated from pTOPO-bluntII in a 1% agarose gel in TAE buffer and finally purified with QIAquick Gel Extraction kit (Qiagen, catalog no. 28706) using standard conditions. The purified cDNA fragments were then inserted into the appropriately digested and purified pPthC vector [19]. We screened the *Escherichia coli *positive clones in which the vector contained the cDNA fragments by enzymatic digestions and then sequencing the positive clones using the universal M13Fw primer and, for longer sequences, internal forward primers specific to the gene of interest.

### Induction of transgene expression

Three positive clones coming from the six-well copy were thawed, amplified and tested for the inducibility of the introduced gene to Tc. The complete removal of Tc results in sufficient induction of the Tet-off system [42]. Cells to be induced were washed twice with PBS, cultured for more than 3 hours in DMEM without Tc, trypsinized and re-plated onto new dishes. Clones were grown in medium deprived of Tc to perform a time course of induction (17, 24, 39 and 48 hours). In the presence of Tc (0 hours), the expression of each mRNA was indicative of the basal expression level in mES cells. Total RNA samples at various times of induction were purified by QIAshredder (catalog no. 79656) and extracted with RNeasy Protect Mini Kit (catalog no. 74126) using standard conditions. Total RNA (1 μg) was reverse-transcribed by QuantiTect Reverse Transcription Kit (Qiagen, catalog no. 205313) according to the manufacturer's instructions. q-PCR experiments were performed using Light Cycler 480 Syber Green I Mastermix (Roche spa, Monza, Italy, catalog no. 04887352001) for cDNA amplification and in LightCycler 480 II (Roche) for signal detection. q-PCR results were analyzed using the comparative Ct method normalized against the housekeeping gene *Actin B*.

All primer pair sequences used for q-PCR are available in Additional file 4. Luciferase assays on mES cells overexpressing the firefly *luciferase *(*Luc*) gene was performed using Dual Luciferase Reporter Assay System (Promega Italia, Milano, Italy). YFP fluorescence assay to detect the expression of the *YFP *reporter was performed using the DM6000 Leica Microscope.

### Karyotyping

The analysis was performed on 20 different inducible clones of our mES cell bank (7 effective and 13 silent genes) and on parental ES cells (EB3) at the beginning of this study on the cell line received from Dr Hitoshi Niwa and again 2 years later. A single inducible clone was chosen randomly within the biological triplicate for this analysis. Cells at 70% confluence were treated with colcemid (Invitrogen) for 2 hours and harvested. Cell pellets were resuspended in pre-warmed hypotonic solution (0.56% KCl) and incubated at 37°C. Cells were then fixed with freshly prepared, ice-cold methanol-acetic acid solution (3:1 in volume) and mounted by dropping onto slides from a height of 1 meter. Metaphase spreads were stained with 5% Giemsa solution (Invitrogen). Approximately 20 images were taken, and 25 spreads were analyzed to assess the percentage of euploid cells.

### Embryonic stem cell differentiation

The EB3 cells and the parental line E14 cells [43] were allowed to differentiate using the 'hanging drop' method [44,45]. The differentiation medium consists of the mES cell medium depleted of LIF. The primer pair of *Oct3/4 *used in q-PCR is reported in Additional file 4.

### Western blotting

Whole cell lysates were extracted after 24 or 48 hours of induction by lysis buffer (50 mM Tris-HCl (pH 8.0), 200 mM NaCl, 1% Triton, 1 mM EDTA, 50 mM Hepes) containing 1% (v/v) of proteinase inhibitor cocktail (Sigma, catalog no. P8340). Thirty micrograms of protein extract from 4 out of 7 clones overexpressing effective genes (*Erg*, *Nrip1*, *Runx1*, *Pdxk*) and 11 out of 13 overexpressing silent genes (*Bach1*, *Ets2*, *Gabpa*, *Olig1*, *Pknox1*, *1810007M14Rik*, *Dscr1-Rcan1*, *DYRK1A*, *Hunk*, *Pfkl*, *Ripk4*) were fractionated on 10% SDS-PAGE gels and electroblotted onto Trans-Blot transfer membrane (Biorad Italy, Segrate, Milano, Italy, catalog no. 162-0112). After incubation in blocking buffer in standard conditions, the membranes were incubated with anti-Flag antibody produced in rabbit (Sigma, catalog no. F7425) and then with anti-rabbit IgG horseradish peroxidase linked whole antibody (Amersham Biosciences, GE Healthcare Europe GmbH, Milano, Italy, catalog no. NA934V). Luminescence was performed using Super Signal West Pico Chemiluminescent substrate (Pierce, Euroclone, Pero, Milano, Italy, catalog no. 34080).

### Microarray hybridization

Total RNA (3 μg) was reverse transcribed to single-stranded cDNA with a special oligo (dT)24 primer containing a T7 RNA promoter site, added 3' to the poly-T tract, prior to second strand synthesis (One Cycle cDNA Synthesis Kit by Affymetrix, Fremont, CA, USA). Biotinylated cRNAs were then generated, using the GeneChip IVT Labeling Kit (Affymetrix). Twenty micrograms of biotinylated cRNA was fragmented and 10 μg hybridized to the Affymetrix GeneChip Mouse Genome 430_2 array for 16 hours at 45°C using an Affymetrix GeneChip Fluidics Station 450 according to the manufacturer's standard protocols.

### Microarray data processing

Low-level analysis to convert probe level data to gene level expression data was done using robust multiarray average (RMA) implemented using the RMA function of the Affymetrix package of the Bioconductor project [46,47] in the R programming language [48]. The low-level analysis for the BAMarray tool was performed using the MAS5 method, implemented using the corresponding function of the same Bioconductor package.

### Statistical analysis of differential gene expression

For each gene, a *t*-test was used on RMA normalized data to determine if there was a significant difference in expression between the two groups of microarrays (induced versus uninduced). *P*-value adjustment for multiple comparisons was done with the FDR of Benjamini-Hochberg [49]. A FDR control was applied to correct for multiple comparisons; the thresholds used in the different cases are reported in the main text. The BAM analysis was performed with BAMarray v3.0. The analysis was performed on MAS5 normalized array data using the default settings except for the following parameters: accuracy was set to high, clustering was set to manual with a value of 25, and variance was set to unequal.

*t*-Tests were also carried out to assess the significance of the variation in the relative expression values of each of the 20 genes analyzed in the parental cell line (EB3) versus the corresponding transgenic inducible clones (in the biological replicates) grown in the presence of Tc (0 hours of induction). In this statistical analysis the threshold for statistical significance chosen was a FDR < 0.05. The apparent increase of expression levels between EB3 cells and the non-induced state (in the cases of *Bach1 *and *Gabpa*, for example) was not statistically significant and therefore can be explained by the biological variability of expression levels of these genes in mES cells. In Additional file 5, we report the comparison of relative expression of 20 genes in the EB3 cell line with the corresponding transgenic inducible clones (in the biological replicates) grown in the presence of Tc (0 hours of induction).

### Microarray data analysis

In the cases of *Runx1 *and *Erg *overexpression, a large number of genes were differentially expressed with FDR <5% (4,585 genes for *Runx1 *and 5,820 for *Erg*). This means that the number of false positives obtained from *Runx1 *and *Erg *experiments are 229 and 291, respectively. In order to reduce the number of false positives, we decided to perform the GO analysis on the gene set obtained while filtering the array using a more stringent criteria (FDR <1%). The differential expression of genes as obtained with the microarray was validated by q-PCR of the most up- and down-regulated genes as ranked by the differential expression ratio. In Additional file 4 we report the primer pair used in q-PCR.

### Gene set enrichment analysis

GSEA [24,50] was performed to determine if the set of silent genes was characterized by above average wild-type expression levels. The analysis was performed on the whole list of 45,102 probesets using the online GSEA server [51] with the default values for all the tool parameters and produced an enrichment score of 0.402 (FDR q-value = 0).

### Protein disorder measurement

The protein disorder was measured using the GlobPlot online tool v2.3 [52,53]. The disorder value for a protein was determined by a summation of the lengths of the disordered regions determined by the tool.

### Comparison with Tc1 cell line

The results of our overexpression experiments were collectively and individually compared with the Tc1 expression data. The MAS4 pre-processed Tc1 data were retrieved from Array Express [ArrayExpress:E-MEXP-654] and subsequently processed according to the same canonical statistical analysis (Cyber-t plus FDR correction; FDR < 5%) as our expression data, yielding a total of 284 significant genes (FDR < 0.05). Since the Tc1 dataset was obtained with a different chipset from ours (MG_U74Av2), we first converted the probesets into their 430_2 equivalents using the Affymetrix 'best match' conversion table; the result of the conversion yielded 241 genes. The probesets selected for each comparison were those that were found to be significant in both the Tc1 and the specific overexpression experiment; the composition of the individual lists is reported in Additional file 16. The total list used for Figure 4 was obtained by merging the individual lists and removing duplicate genes by keeping the maximum in absolute value and discarding the others, yielding 168 genes. The scatter plots were obtained by plotting the logarithm of the Tc1 fold change (ratio of treated versus untreated cell line) on the *x *axis, and the logarithm of the overexpressed gene on the *y *axis. The regression line coefficients were obtained using an algorithm computing a non-centered version of the correlation coefficient (the *xcorr *Matlab function) for the individual plots, and a standard A = YX^-1 ^algorithm for the collective plot (the two algorithms are interchangeable). The *P*-value for the regression coefficients was computed using a Student's *t *distribution for a transformation of the correlation. A *P*-value indicating the probability of obtaining the shown ratio of same-sign over total dots purely by chance was computed as follows. A set of *n *(*x*, *y*) pairs was created by randomly extracting *x *from the list of Tc1 log ratio values and *y *from the list of current gene values, where *n *is the number of dots in the graph; 100,000 such sets were created (1 million in the case of Aire), and the percentage of sets for which *x *× *y *> 0 was true for at least *k *out of the *n *pairs was noted and taken as *P*-value, where *k *is the number of dots in the graph having same-sign coordinates.

### Large-gel two-dimensional protein electrophoresis

The total protein extraction from mES cells was carried out using our standard protocol [54]. Protein (70 μg) was separated in each 2DGE run. Transgenic and parental cell lines were always run in parallel. The proteomic analysis was carried out on two Runx1 overexpressing clones (E6 and E7) out of the three clones (E6, E7 and F3) used for the transcriptome analysis (Additional file 3). Three technical repeats were performed for each clone. Overall, 12 two-dimensional gels were run for each Runx1 overexpressing clone: 6 replicates for the non-induced state and 6 replicates for the induced state (48 hours). All of the above samples were always run simultaneously in the same electrophoresis chamber to ensure gel pattern comparability. The protein expression alterations upon Runx1 overexpression were calculated by the ratio of the t48 hours mean to the t0 hours mean, using the averaged values across six gels (three technical replicates of each biological replicate). The statistic significance was accessed by student's *t*-test, with *P *< 0.05, and in addition, only if there is an expression alteration greater than 20% as described in [55]. Silver staining protocol was employed to visualize protein spots [56]. Computer-assisted gel evaluation was performed (Delta2D v3.4, Decodon, Greifswald Germany). Briefly, 2DGE gels were scanned at high resolution (600 dpi; TMA 1600, Microtek, Willich, Germany). Corresponding gel images were first warped using 'exact mode' (manual vector setting combined with automatic warping). A fusion gel image was subsequently generated using 'union mode', which is a weighted arithmetic mean across the entire gel series. Spot detection was carried out on this fusion image automatically, followed by manual spot editing. Subsequently, spots were transferred from fusion image to all gels. The signal intensities (volume of each spot) were computed as a weighted sum of all pixel intensities of each protein spot. Percent volume of spot intensities calculated as a fraction of the total spot volume of the parent gel was used for quantitative analysis of protein expression level. Normalized values after local background extraction were subsequently exported from Delta2D in spreadsheet format for statistical analysis. Student's *t*-test was carried out for control versus induced cell lines to access statistical significance of the expression differences (pair-wise, two-sided). *P *< 0.05 was used as statistical significance threshold. To reduce the influence of data noise, only protein expression changes over 20% compared to control were retained for further analysis. Additional file 22 shows the raw data of the proteomic analysis by 2DGE following the overexpression of Runx1. The detailed spot quantification data, in the form of relative volume data of each spot on each individual 2DGE gel, are also provided in this table. 2DGE gel image data have now been submitted to the World-2DPAGE Repository of the ExPASy Proteomics Server [2DPAGE:0021] for public access [31].

### Mass spectrometric protein identification

For protein identification by mass spectrometry, high resolution 2DGE gels were stained using a mass spectrometry compatible silver staining protocol [57]. Protein spots of interest were excised and subjected to in-gel trypsin digestion without reduction and alkylation. Tryptic fragments were analyzed using a LCQ Deca XP nano HPLC/ESI ion trap mass spectrometer (Thermo Fisher Scientific, Waltham, MA, USA) as described previously [58]. For database-assisted protein identification, monoisotopic mass values of peptides were searched against NCBInr (version 20061206, taxonomy *Mus musculus*), allowing one missed cleavage. Peptide mass tolerance and fragment mass tolerance were set at 0.8 Dalton. Oxidation of methionine and arylamide adducts on cysteine (propionaide) were considered as variable peptide modifications. Criteria for positive identification of proteins were set according to the scoring algorithm delineated in Mascot (Matrix Science, London, UK) [59], with an individual ion score cut-off threshold corresponding to *P *< 0.05.

## Abbreviations

2DGE: two-dimensional gel electrophoresis; DMEM: Dulbecco's modified Eagle's medium; DS: Down syndrome; FDR: false discovery rate; GO: Gene Ontology; GSEA: gene set enrichment analysis; HSA21: human chromosome 21; LIF: leukemia inhibitory factor; mES: mouse embryonic stem; ORF: open reading frame; PBS: phosphate-buffered saline; q-PCR: quantitative PCR; RMA: robust multiarray average; RMCE: recombination-mediated cassette exchange; Tc: tetracycline; YFP: yellow fluorescent protein.

## Authors' contributions

RDC and AR contributed equally to this work. RDC, AR and SI provided material, experimentation, data collection and analysis. RDC participated in writing the manuscript. LM and ML provided intellectual input for experimentation and data analysis. AOF provided technical input with respect to cloning. GC, DdB, SB and AB participated in writing the manuscript and intellectual input.

## Supplementary Material

Additional file 1 **Identification and validation of inducible/exchangeable recombinant mES clones**. **(a) **Recombinant mES clones were identified by PCR analysis. **(b) **q-PCR analysis and Luciferase assays using Dual Luciferase Reporter Assay System was performed on mES clones overexpressing the firefly *luciferase *(*Luc*) gene. The system was activated upon the removal of Tc (after 17, 24, 39 and 48 hours) from the medium. Protein extracts of mES cells were prepared at the same time points and luminescence quantified. **(c) **q-PCR analysis and YFP fluorescence assay to detect the expression of the *YFP *reporter. **(d) **Expression of mES cells overexpressing *Luc *after 24 hours from the complete removal of Tc from the medium; the degree of induction was easily manipulated by titrating the Tc. **(e) **Expression profile (q-PCR) of the pluripotency gene *Oct3/4*, and of markers of the mesoderm (*Brachyury*), ectoderm (*Gfap*) and endoderm (*Afp*) during differentiation of EB3 and of the parent cell line (E14). Click here for file

Additional file 2 **List of 32 genes overexpressed in mouse ES cells**. In this table we list the 32 genes selected to be integrated in the *Rosa26 *locus and overexpressed using the Tet-off system in mES cells. Click here for file

Additional file 3 **Time course of induction of three clones (biological replicates) selected for each gene**. In this table we report the time course of the induction of mES clones that overexpress the 32 ORFs. For each gene, three drug-resistant mES biological replicates, whose names are indicated in the specific column, were selected to be tested for their sensitivity to Tc removal from the medium. Click here for file

Additional file 4 **Primer pairs used in q-PCR**. Click here for file

Additional file 5 **Comparison of relative expression levels for 20 genes in the EB3 parental cell line and in the inducible clones at 0 hours of induction by multiple statistical *t*-tests**. In this table we show the comparison of the relative expression of 20 genes (the 13 transcription factors, the single transcriptional activator and the 6 kinases) in the EB3 cell line versus the corresponding transgenic inducible clones (in the biological replicates) grown in the presence of Tc (0 hours of induction). Click here for file

Additional file 6 **Complete list of differentially expressed genes following the overexpression of *Aire*, one of the effective genes**. Click here for file

Additional file 7 **Complete list of differentially expressed genes following the overexpression of *Erg*, one of the effective genes**. Click here for file

Additional file 8 **Complete list of differentially expressed genes following the overexpression of *Nrip1*, one of the effective genes**. Click here for file

Additional file 9 **Complete list of differentially expressed genes following the overexpression of *Olig2*, one of the effective genes**. Click here for file

Additional file 10 **Complete list of differentially expressed genes following the overexpression of *Pdxk*, one of the effective genes**. Click here for file

Additional file 11 **Complete list of differentially expressed genes following the overexpression of *Runx1*, one of the effective genes**. Click here for file

Additional file 12 **Complete list of differentially expressed genes following the overexpression of *Sim2*, one of the effective genes**. Click here for file

Additional file 13 **Summary of q-PCR validation of microarray data**. In this table we show the validation by q-PCR of the differential expression of a subset of the most up-regulated and down-regulated genes detected by microarray analysis of the seven effective genes, as ranked by differential expression ratio. Click here for file

Additional file 14 **A complete list of significantly enriched GO terms for the seven effective genes**. Click here for file

Additional file 15 **List of all the mouse orthologs of HSA21 genes sorted according to their basal expression level in mES cells (from the most to the least expressed)**. Click here for file

Additional file 16 **Summary of results derived from the comparison between the analysis of mES overexpressing effective clones and the transchromosomic Tc1 mouse line**. Click here for file

Additional file 17 **Comparison of overexpression experiments with the transcriptional response of the transchromosomic tc1 mouse line**. X-Y graphs comparing the transcriptional response of Tc1 with the response obtained in the individual overexpression experiments. Each dot represents a gene whose expression was statistically significant in both the Tc1 and the indicated overexpression experiment. The *x *axis corresponds to the log of the Tc1 ratio (trisomic versus wild type), and the *y *axis corresponds to the log of the ratio in the overexpression experiment (induced versus non-induced clone). The ratio of same-sign over total dots is reported for each graph. Click here for file

Additional file 18 **A complete list of GO terms significantly enriched in the subsets of genes differentially expressed after overexpression of 11 out of 13 silent genes**. Click here for file

Additional file 19 **GO enrichment analysis for five (*Bach1*, *Dscr1-Rcan1*, *DYRK1A*, *Gabpa *and *SNF1LK*) out of thirteen silent genes, as assessed by microarray analysis**. In this table we report the GO enrichment analysis for five out of thirteen silent genes (*Bach1*, *Dscr1-Rcan1*, *DYRK1A*, *Gabpa*, *SNF1LK*); supporting references for a subset of significant biological processes identified by the GO analysis are given. Click here for file

Additional file 20 **Differential protein expression variation in mES cells overexpressing *Runx1***. In this table we report the complete list of the proteins whose expression changed following the induction of *Runx1* Click here for file

Additional file 21 **Primer pairs used in PCR**. Click here for file

Additional file 22 **Raw data of the proteomic analysis, using 2DGE, following the induction of two Runx1 overexpressing clones (E6 and E7)**. Three technical repeats were performed for each biological replicate: this comprises 6 gels for E6 and 6 gels for E7. For each clone we ran three replicates for t0 (control) and three replicates for t48 hours. Corresponding t0 and t48 hour samples were always run simultaneously in the same chamber to ensure gel pattern comparability. The detailed spot quantification data are provided in this table. We list the relative volume of each spot on each individual 2DGE gel, together with other spot parameters, such as pixel spot volume, *x *and *y *coordinates of spots on the fusion gel image, as well as spot quality index. Click here for file

## References

[B1] Jacobs PA Chromosome mutations: frequency at birth in humans. Humangenetik 1972 16 137 140 10.1007/BF00393999 4647438

[B2] Goad WB Robinson A Puck TT Incidence of aneuploidy in a human population. Am J Hum Genet 1976 28 62 68 1247021 PMC1684903

[B3] Hassold T Hunt P To err (meiotically) is human: the genesis of human aneuploidy. Nat Rev Genet 2001 2 280 291 10.1038/35066065 11283700

[B4] Adler ID Aneuploidy studies in mammals. Prog Clin Biol Res 1990 340B 285 293 2203010

[B5] Griffin DK The incidence, origin, and etiology of aneuploidy. Int Rev Cytol 1996 167 263 296 full_text 8768496 10.1016/s0074-7696(08)61349-2

[B6] Torres EM Williams BR Amon A Aneuploidy: cells losing their balance. Genetics 2008 179 737 746 10.1534/genetics.108.090878 18558649 PMC2429870

[B7] Wiseman FK Alford KA Tybulewicz VL Fisher EM Down syndrome - recent progress and future prospects. Hum Mol Genet 2009 18 R75 83 10.1093/hmg/ddp010 19297404 PMC2657943

[B8] Patterson D Molecular genetic analysis of Down syndrome. Hum Genet 2009 126 195 214 10.1007/s00439-009-0696-8 19526251

[B9] Korbel JO Tirosh-Wagner T Urban AE Chen XN Kasowski M Dai L Grubert F Erdman C Gao MC Lange K Sobel EM Barlow GM Aylsworth AS Carpenter NJ Clark RD Cohen MY Doran E Falik-Zaccai T Lewin SO Lott IT McGillivray BC Moeschler JB Pettenati MJ Pueschel SM Rao KW Shaffer LG Shohat M Van Riper AJ Warburton D Weissman S The genetic architecture of Down syndrome phenotypes revealed by high-resolution analysis of human segmental trisomies. Proc Natl Acad Sci USA 2009 106 12031 12036 10.1073/pnas.0813248106 19597142 PMC2709665

[B10] Arron JR Winslow MM Polleri A Chang CP Wu H Gao X Neilson JR Chen L Heit JJ Kim SK Yamasaki N Miyakawa T Francke U Graef IA Crabtree GR NFAT dysregulation by increased dosage of DSCR1 and DYRK1A on chromosome 21. Nature 2006 441 595 600 10.1038/nature04678 16554754

[B11] Reeves RH Irving NG Moran TH Wohn A Kitt C Sisodia SS Schmidt C Bronson RT Davisson MT A mouse model for Down syndrome exhibits learning and behaviour deficits. Nat Genet 1995 11 177 184 10.1038/ng1095-177 7550346

[B12] Baek KH Zaslavsky A Lynch RC Britt C Okada Y Siarey RJ Lensch MW Park IH Yoon SS Minami T Korenberg JR Folkman J Daley GQ Aird WC Galdzicki Z Ryeom S Down's syndrome suppression of tumour growth and the role of the calcineurin inhibitor DSCR1. Nature 2009 459 1126 1130 10.1038/nature08062 19458618 PMC2724004

[B13] Altafaj X Dierssen M Baamonde C Marti E Visa J Guimera J Oset M Gonzalez JR Florez J Fillat C Estivill X Neurodevelopmental delay, motor abnormalities and cognitive deficits in transgenic mice overexpressing Dyrk1A (minibrain), a murine model of Down's syndrome. Hum Mol Genet 2001 10 1915 1923 10.1093/hmg/10.18.1915 11555628

[B14] Voronov SV Frere SG Giovedi S Pollina EA Borel C Zhang H Schmidt C Akeson EC Wenk MR Cimasoni L Arancio O Davisson MT Antonarakis SE Gardiner K De Camilli P Di Paolo G Synaptojanin 1-linked phosphoinositide dyshomeostasis and cognitive deficits in mouse models of Down's syndrome. Proc Natl Acad Sci USA 2008 105 9415 9420 10.1073/pnas.0803756105 18591654 PMC2453748

[B15] Rachidi M Lopes C Charron G Delezoide AL Paly E Bloch B Delabar JM Spatial and temporal localization during embryonic and fetal human development of the transcription factor SIM2 in brain regions altered in Down syndrome. Int J Dev Neurosci 2005 23 475 484 10.1016/j.ijdevneu.2005.05.004 15946822

[B16] Mensah A Mulligan C Linehan J Ruf S O'Doherty A Grygalewicz B Shipley J Groet J Tybulewicz V Fisher E Brandner S Nizetic D An additional human chromosome 21 causes suppression of neural fate of pluripotent mouse embryonic stem cells in a teratoma model. BMC Dev Biol 2007 7 131 10.1186/1471-213X-7-131 18047653 PMC2211317

[B17] Pinter JD Brown WE Eliez S Schmitt JE Capone GT Reiss AL Amygdala and hippocampal volumes in children with Down syndrome: a high-resolution MRI study. Neurology 2001 56 972 974 11294940 10.1212/wnl.56.7.972

[B18] Canzonetta C Mulligan C Deutsch S Ruf S O'Doherty A Lyle R Borel C Lin-Marq N Delom F Groet J Schnappauf F De Vita S Averill S Priestley JV Martin JE Shipley J Denyer G Epstein CJ Fillat C Estivill X Tybulewicz VL Fisher EM Antonarakis SE Nizetic D DYRK1A-dosage imbalance perturbs NRSF/REST levels, deregulating pluripotency and embryonic stem cell fate in Down syndrome. Am J Hum Genet 2008 83 388 400 10.1016/j.ajhg.2008.08.012 18771760 PMC2556438

[B19] Masui S Shimosato D Toyooka Y Yagi R Takahashi K Niwa H An efficient system to establish multiple embryonic stem cell lines carrying an inducible expression unit. Nucleic Acids Res 2005 33 e43 10.1093/nar/gni043 15741176 PMC552969

[B20] Yu T Li Z Jia Z Clapcote SJ Liu C Li S Asrar S Pao A Chen R Fan N Carattini-Rivera S Bechard AR Spring S Henkelman RM Stoica G Matsui S Nowak NJ Roder JC Chen C Bradley A Yu YE A mouse model of Down syndrome trisomic for all human chromosome 21 syntenic regions. Hum Mol Genet 2010 19 2780 2791 20442137 10.1093/hmg/ddq179 PMC2893810

[B21] Dennis G Sherman BT Jr Hosack DA Yang J Gao W Lane HC Lempicki RA DAVID: Database for Annotation, Visualization, and Integrated Discovery. Genome Biol 2003 4 P3 10.1186/gb-2003-4-5-p3 12734009 PMC193660

[B22] Huang da W Sherman BT Lempicki RA Systematic and integrative analysis of large gene lists using DAVID bioinformatics resources. Nat Protoc 2009 4 44 57 10.1038/nprot.2008.211 19131956

[B23] DAVID http://david.abcc.ncifcrf.gov

[B24] Subramanian A Tamayo P Mootha VK Mukherjee S Ebert BL Gillette MA Paulovich A Pomeroy SL Golub TR Lander ES Mesirov JP Gene set enrichment analysis: a knowledge-based approach for interpreting genome-wide expression profiles. Proc Natl Acad Sci USA 2005 102 15545 15550 10.1073/pnas.0506580102 16199517 PMC1239896

[B25] Vavouri T Semple JI Garcia-Verdugo R Lehner B Intrinsic protein disorder and interaction promiscuity are widely associated with dosage sensitivity. Cell 2009 138 198 208 10.1016/j.cell.2009.04.029 19596244

[B26] O'Doherty A Ruf S Mulligan C Hildreth V Errington ML Cooke S Sesay A Modino S Vanes L Hernandez D Linehan JM Sharpe PT Brandner S Bliss TV Henderson DJ Nizetic D Tybulewicz VL Fisher EM An aneuploid mouse strain carrying human chromosome 21 with Down syndrome phenotypes. Science 2005 309 2033 2037 10.1126/science.1114535 16179473 PMC1378183

[B27] BAMarray 3.0 http://www.bamarray.com/

[B28] Ishwaran H Rao JS Spike and slab gene selection for multigroup microarray data. J Am Stat Assoc 2005 100 764 780 10.1198/016214505000000051

[B29] Ishwaran H Rao JS Kogalur UB BAMarraytrade mark: Java software for Bayesian analysis of variance for microarray data. BMC Bioinformatics 2006 7 59 10.1186/1471-2105-7-59 16466568 PMC1382258

[B30] ExPASy Proteomic Server http://world-2dpage.expasy.org/repository/

[B31] Hoogland C Mostaguir K Appel RD Lisacek F The World-2DPAGE Constellation to promote and publish gel-based proteomics data through the ExPASy server. J Proteomics 2008 71 245 248 10.1016/j.jprot.2008.02.005 18617148

[B32] Kahlem P Sultan M Herwig R Steinfath M Balzereit D Eppens B Saran NG Pletcher MT South ST Stetten G Lehrach H Reeves RH Yaspo ML Transcript level alterations reflect gene dosage effects across multiple tissues in a mouse model of down syndrome. Genome Res 2004 14 1258 1267 10.1101/gr.1951304 15231742 PMC442140

[B33] Suda Y Suzuki M Ikawa Y Aizawa S Mouse embryonic stem cells exhibit indefinite proliferative potential. J Cell Physiol 1987 133 197 201 10.1002/jcp.1041330127 3667706

[B34] Palmqvist L Glover CH Hsu L Lu M Bossen B Piret JM Humphries RK Helgason CD Correlation of murine embryonic stem cell gene expression profiles with functional measures of pluripotency. Stem Cells 2005 23 663 680 10.1634/stemcells.2004-0157 15849174

[B35] Munne S Howles CM Wells D The role of preimplantation genetic diagnosis in diagnosing embryo aneuploidy. Curr Opin Obstet Gynecol 2009 21 442 449 10.1097/GCO.0b013e32832fad73 19606031

[B36] Prandini P Deutsch S Lyle R Gagnebin M Delucinge Vivier C Delorenzi M Gehrig C Descombes P Sherman S Dagna Bricarelli F Baldo C Novelli A Dallapiccola B Antonarakis SE Natural gene-expression variation in Down syndrome modulates the outcome of gene-dosage imbalance. Am J Hum Genet 2007 81 252 263 10.1086/519248 17668376 PMC1950802

[B37] Nishiyama A Xin L Sharov AA Thomas M Mowrer G Meyers E Piao Y Mehta S Yee S Nakatake Y Stagg C Sharova L Correa-Cerro LS Bassey U Hoang H Kim E Tapnio R Qian Y Dudekula D Zalzman M Li M Falco G Yang HT Lee SL Monti M Stanghellini I Islam MN Nagaraja R Goldberg I Wang W Uncovering early response of gene regulatory networks in ESCs by systematic induction of transcription factors. Cell Stem Cell 2009 5 420 433 10.1016/j.stem.2009.07.012 19796622 PMC2770715

[B38] Mortazavi A Williams BA McCue K Schaeffer L Wold B Mapping and quantifying mammalian transcriptomes by RNA-Seq. Nat Methods 2008 5 621 628 10.1038/nmeth.1226 18516045 PMC13303166

[B39] Ahn KJ Jeong HK Choi HS Ryoo SR Kim YJ Goo JS Choi SY Han JS Ha I Song WJ DYRK1A BAC transgenic mice show altered synaptic plasticity with learning and memory defects. Neurobiol Dis 2006 22 463 472 10.1016/j.nbd.2005.12.006 16455265

[B40] Vega RB Rothermel BA Weinheimer CJ Kovacs A Naseem RH Bassel-Duby R Williams RS Olson EN Dual roles of modulatory calcineurin-interacting protein 1 in cardiac hypertrophy. Proc Natl Acad Sci USA 2003 100 669 674 10.1073/pnas.0237225100 12515860 PMC141054

[B41] Araki K Imaizumi T Okuyama K Oike Y Yamamura K Efficiency of recombination by Cre transient expression in embryonic stem cells: comparison of various promoters. J Biochem 1997 122 977 982 9443813 10.1093/oxfordjournals.jbchem.a021860

[B42] Rennel E Gerwins P How to make tetracycline-regulated transgene expression go on and off. Anal Biochem 2002 309 79 84 10.1016/S0003-2697(02)00250-6 12381365

[B43] Hooper M Hardy K Handyside A Hunter S Monk M HPRT-deficient (Lesch-Nyhan) mouse embryos derived from germline colonization by cultured cells. Nature 1987 326 292 295 10.1038/326292a0 3821905

[B44] Wobus AM Guan K Yang HT Boheler KR Embryonic stem cells as a model to study cardiac, skeletal muscle, and vascular smooth muscle cell differentiation. Methods Mol Biol 2002 185 127 156 11768985 10.1385/1-59259-241-4:127

[B45] Wobus AM Boheler KR Embryonic stem cells: prospects for developmental biology and cell therapy. Physiol Rev 2005 85 635 678 10.1152/physrev.00054.2003 15788707

[B46] Bioconductor http://www.R-project.org

[B47] Gentleman RC Carey VJ Bates DM Bolstad B Dettling M Dudoit S Ellis B Gautier L Ge Y Gentry J Hornik K Hothorn T Huber W Iacus S Irizarry R Leisch F Li C Maechler M Rossini AJ Sawitzki G Smith C Smyth G Tierney L Yang JY Zhang J Bioconductor: open software development for computational biology and bioinformatics. Genome Biol 2004 5 R80 10.1186/gb-2004-5-10-r80 15461798 PMC545600

[B48] R: A Language and Environment for Statistical Computing http://cran.r-project.org/doc/manuals/refman.pdf

[B49] Klipper-Aurbach Y Wasserman M Braunspiegel-Weintrob N Borstein D Peleg S Assa S Karp M Benjamini Y Hochberg Y Laron Z Mathematical formulae for the prediction of the residual beta cell function during the first two years of disease in children and adolescents with insulin-dependent diabetes mellitus. Med Hypotheses 1995 45 486 490 10.1016/0306-9877(95)90228-7 8748093

[B50] Mootha VK Lindgren CM Eriksson KF Subramanian A Sihag S Lehar J Puigserver P Carlsson E Ridderstrale M Laurila E Houstis N Daly MJ Patterson N Mesirov JP Golub TR Tamayo P Spiegelman B Lander ES Hirschhorn JN Altshuler D Groop LC PGC-1alpha-responsive genes involved in oxidative phosphorylation are coordinately downregulated in human diabetes. Nat Genet 2003 34 267 273 10.1038/ng1180 12808457

[B51] GSEA http://www.broad.mit.edu/gsea/

[B52] Linding R Russell RB Neduva V Gibson TJ GlobPlot:Exploring protein sequences for globularity and disorder. Nucleic Acids Res 2003 31 3701 3708 10.1093/nar/gkg519 12824398 PMC169197

[B53] GlobPlot http://globplot.embl.de/

[B54] Klose J Large-gel 2-D electrophoresis. Methods Mol Biol 1999 112 147 172 10027239 10.1385/1-59259-584-7:147

[B55] Challapalli KK Zabel C Schuchhardt J Kaindl AM Klose J Herzel H High reproducibility of large-gel two-dimensional electrophoresis. Electrophoresis 2004 25 3040 3047 10.1002/elps.200405979 15349946

[B56] Heukeshoven J Dernick R Improved silver staining procedure for fast staining in PhastSystem Development Unit. I. Staining of sodium dodecyl sulfate gels. Electrophoresis 1988 9 28 32 10.1002/elps.1150090106 2466645

[B57] Nebrich G Herrmann M Sagi D Klose J Giavalisco P High MS-compatibility of silver nitrate-stained protein spots from 2-DE gels using ZipPlates and AnchorChips for successful protein identification. Electrophoresis 2007 28 1607 1614 10.1002/elps.200600656 17447244

[B58] Zabel C Mao L Woodman B Rohe M Wacker MA Klare Y Koppelstatter A Nebrich G Klein O Grams S Strand A Luthi-Carter R Hartl D Klose J Bates GP A large number of protein expression changes occur early in life and precede phenotype onset in a mouse model for huntington disease. Mol Cell Proteomics 2009 8 720 734 10.1074/mcp.M800277-MCP200 19043139 PMC2667354

[B59] Pappin DJ Hojrup P Bleasby AJ Rapid identification of proteins by peptide-mass fingerprinting. Curr Biol 1993 3 327 332 10.1016/0960-9822(93)90195-T 15335725

[B60] Miyoshi H Shimizu K Kozu T Maseki N Kaneko Y Ohki M t(8;21) breakpoints on chromosome 21 in acute myeloid leukemia are clustered within a limited region of a single gene, AML1. Proc Natl Acad Sci USA 1991 88 10431 10434 10.1073/pnas.88.23.10431 1720541 PMC52942

[B61] Antonarakis SE Lyle R Dermitzakis ET Reymond A Deutsch S Chromosome 21 and down syndrome: from genomics to pathophysiology. Nat Rev Genet 2004 5 725 738 10.1038/nrg1448 15510164

[B62] Wang Q Stacy T Binder M Marin-Padilla M Sharpe AH Speck NA Disruption of the Cbfa2 gene causes necrosis and hemorrhaging in the central nervous system and blocks definitive hematopoiesis. Proc Natl Acad Sci USA 1996 93 3444 3449 10.1073/pnas.93.8.3444 8622955 PMC39628

[B63] Okuda T van Deursen J Hiebert SW Grosveld G Downing JR AML1, the target of multiple chromosomal translocations in human leukemia, is essential for normal fetal liver hematopoiesis. Cell 1996 84 321 330 10.1016/S0092-8674(00)80986-1 8565077

[B64] Stein GS Lian JB van Wijnen AJ Stein JL Montecino M Javed A Zaidi SK Young DW Choi JY Pockwinse SM Runx2 control of organization, assembly and activity of the regulatory machinery for skeletal gene expression. Oncogene 2004 23 4315 4329 10.1038/sj.onc.1207676 15156188

[B65] Silva FP Swagemakers SM Erpelinck-Verschueren C Wouters BJ Delwel R Vrieling H van der Spek P Valk PJ Giphart-Gassler M Gene expression profiling of minimally differentiated acute myeloid leukemia: M0 is a distinct entity subdivided by RUNX1 mutation status. Blood 2009 114 3001 3007 10.1182/blood-2009-03-211334 19666867

[B66] Remy P Baltzinger M The Ets-transcription factor family in embryonic development: lessons from the amphibian and bird. Oncogene 2000 19 6417 6431 10.1038/sj.onc.1204044 11175358

[B67] Randi AM Sperone A Dryden NH Birdsey GM Regulation of angiogenesis by ETS transcription factors. Biochem Soc Trans 2009 37 1248 1253 10.1042/BST0371248 19909256

[B68] Vlaeminck-Guillem V Vanacker JM Verger A Tomavo N Stehelin D Laudet V Duterque-Coquillaud M Mutual repression of transcriptional activation between the ETS-related factor ERG and estrogen receptor. Oncogene 2003 22 8072 8084 10.1038/sj.onc.1207094 14603248

[B69] Kiskinis E Hallberg M Christian M Olofsson M Dilworth SM White R Parker MG RIP140 directs histone and DNA methylation to silence Ucp1 expression in white adipocytes. EMBO J 2007 26 4831 4840 10.1038/sj.emboj.7601908 17972916 PMC2099470

[B70] Zhang X Szabo E Michalak M Opas M Endoplasmic reticulum stress during the embryonic development of the central nervous system in the mouse. Int J Dev Neurosci 2007 25 455 463 17913437 10.1016/j.ijdevneu.2007.08.007

[B71] Augereau P Badia E Fuentes M Rabenoelina F Corniou M Derocq D Balaguer P Cavailles V Transcriptional regulation of the human NRIP1/RIP140 gene by estrogen is modulated by dioxin signalling. Mol Pharmacol 2006 69 1338 1346 10.1124/mol.105.017376 16391242

[B72] Lu QR Yuk D Alberta JA Zhu Z Pawlitzky I Chan J McMahon AP Stiles CD Rowitch DH Sonic hedgehog--regulated oligodendrocyte lineage genes encoding bHLH proteins in the mammalian central nervous system. Neuron 2000 25 317 329 10.1016/S0896-6273(00)80897-1 10719888

[B73] Zhou Q Wang S Anderson DJ Identification of a novel family of oligodendrocyte lineage-specific basic helix-loop-helix transcription factors. Neuron 2000 25 331 343 10.1016/S0896-6273(00)80898-3 10719889

[B74] Watson ED Mattar P Schuurmans C Cross JC Neural stem cell self-renewal requires the Mrj co-chaperone. Dev Dyn 2009 238 2564 2574 10.1002/dvdy.22088 19777589

[B75] Fu J Tay SS Ling EA Dheen ST High glucose alters the expression of genes involved in proliferation and cell-fate specification of embryonic neural stem cells. Diabetologia 2006 49 1027 1038 10.1007/s00125-006-0153-3 16508779

[B76] Cooper AJ Meister A An appreciation of Professor Alexander E. Braunstein. The discovery and scope of enzymatic transamination. Biochimie 1989 71 387 404 10.1016/0300-9084(89)90169-7 2503044

[B77] Moya-Garcia AA Medina MA Sanchez-Jimenez F Mammalian histidine decarboxylase: from structure to function. Bioessays 2005 27 57 63 10.1002/bies.20174 15612036

[B78] Muller IB Wu F Bergmann B Knockel J Walter RD Gehring H Wrenger C Poisoning pyridoxal 5-phosphate-dependent enzymes: a new strategy to target the malaria parasite *Plasmodium falciparum*. PLoS One 2009 4 e4406 10.1371/journal.pone.0004406 19197387 PMC2634962

[B79] Hansenne I Louis C Martens H Dorban G Charlet-Renard C Peterson P Geenen V Aire and Foxp3 expression in a particular microenvironment for T cell differentiation. Neuroimmunomodulation 2009 16 35 44 10.1159/000179665 19077444

[B80] Laan M Kisand K Kont V Moll K Tserel L Scott HS Peterson P Autoimmune regulator deficiency results in decreased expression of CCR4 and CCR7 ligands and in delayed migration of CD4 + thymocytes. J Immunol 2009 183 7682 7691 10.4049/jimmunol.0804133 19923453 PMC2795747

