# Supplementary figures and images for: A mouse embryonic stem cell bank for inducible overexpression of human chromosome 21 genes
Source: Genome Biol. 2010 Jun 22;11(6):R64. doi: 10.1186/gb-2010-11-6-r64 (PMC2911112; doi:10.1186/gb-2010-11-6-r64)

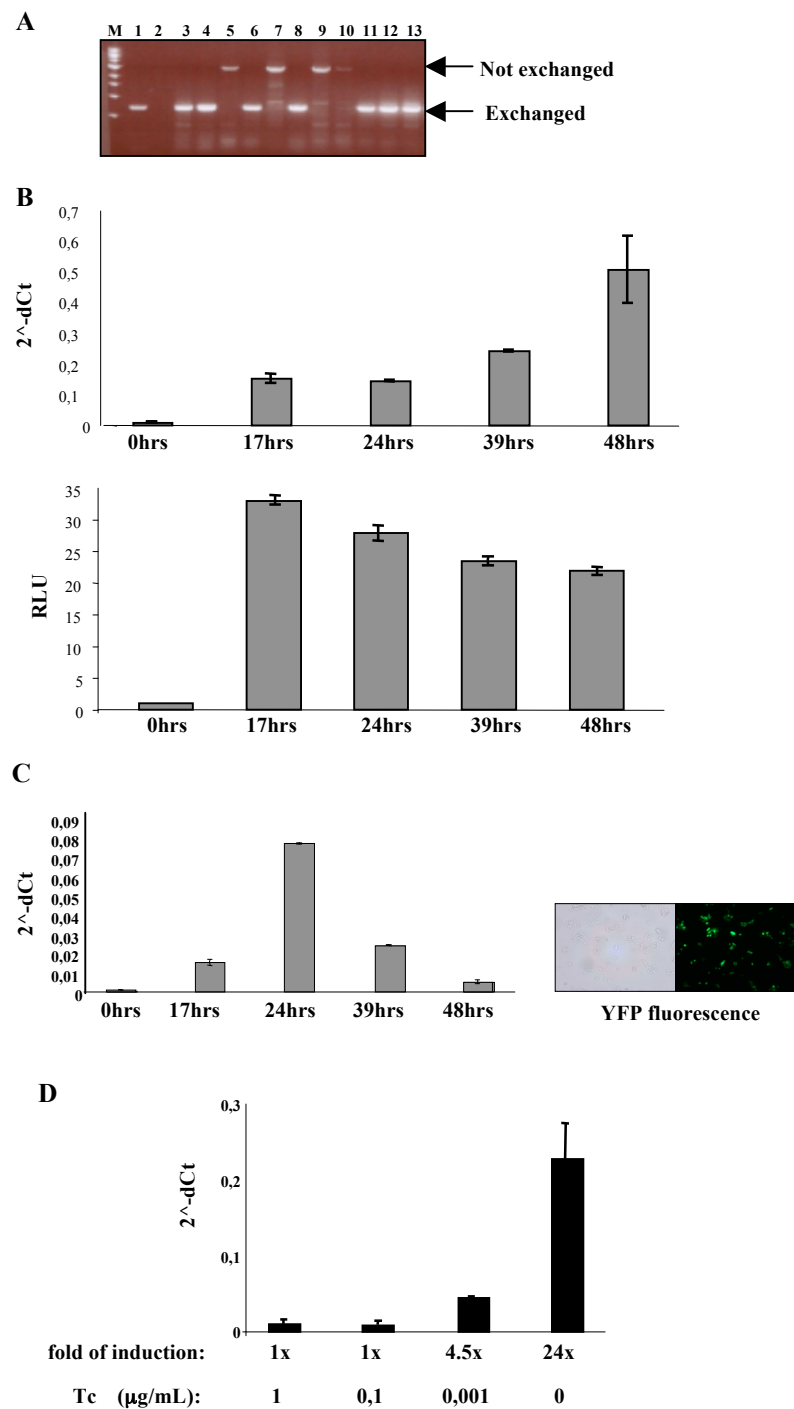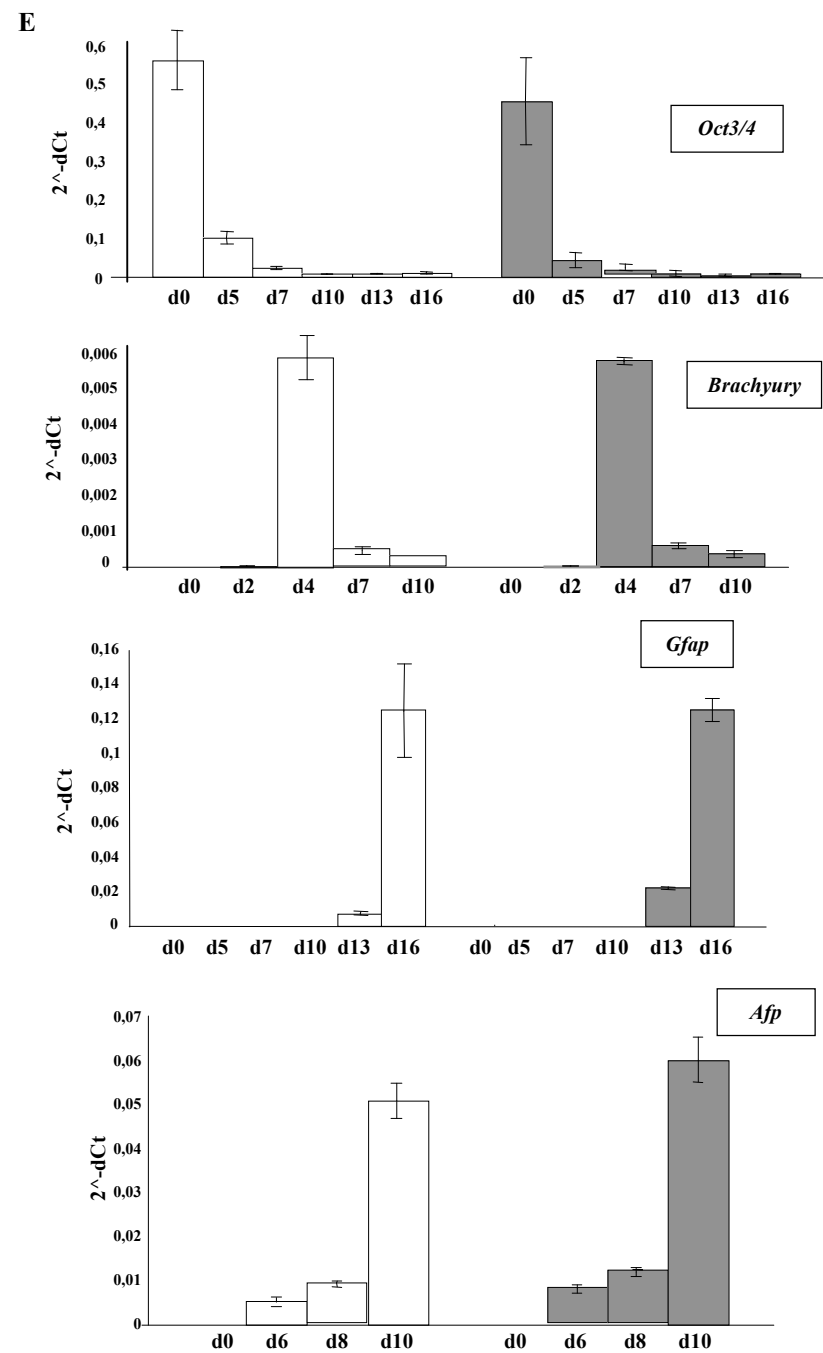

Supplement: Additional file 1 — Identification and validation of inducible/exchangeable recombinant mES clones. (a) Recombinant mES clones were identified by PCR analysis. (b) q-PCR analysis and Luciferase assays using Dual Luciferase Reporter Assay System was performed on mES clones overexpressing the firefly luciferase (Luc) gene. The system was activated upon the removal of Tc (after 17, 24, 39 and 48 hours) from the medium. Protein extracts of mES cells were prepared at the same time points and luminescence quantified. (c) q-PCR analysis and YFP fluorescence assay to detect the expression of the YFP reporter. (d) Expression of mES cells overexpressing Luc after 24 hours from the complete removal of Tc from the medium; the degree of induction was easily manipulated by titrating the Tc. (e) Expression profile (q-PCR) of the pluripotency gene Oct3/4, and of markers of the mesoderm (Brachyury), ectoderm (Gfap) and endoderm (Afp) during differentiation of EB3 and of the parent cell line (E14). [file gb-2010-11-6-r64-S1.PDF]

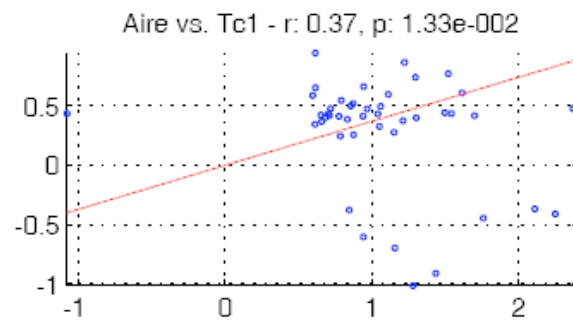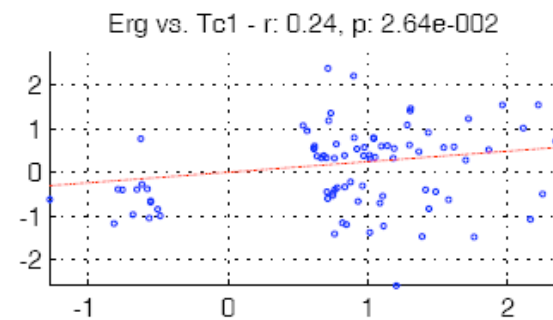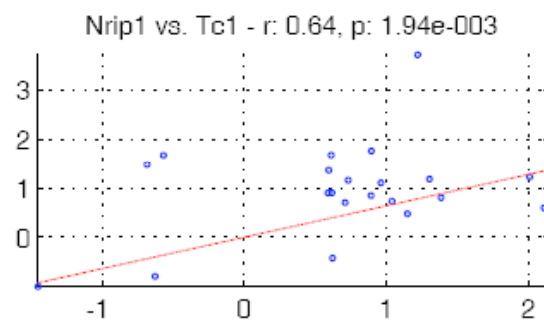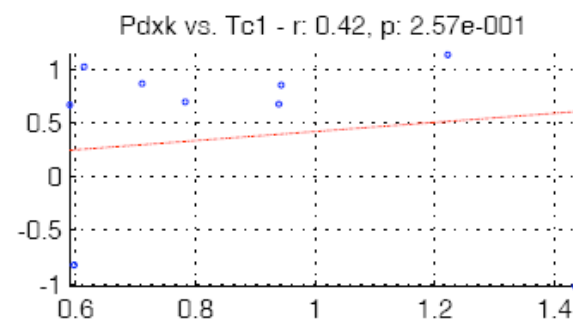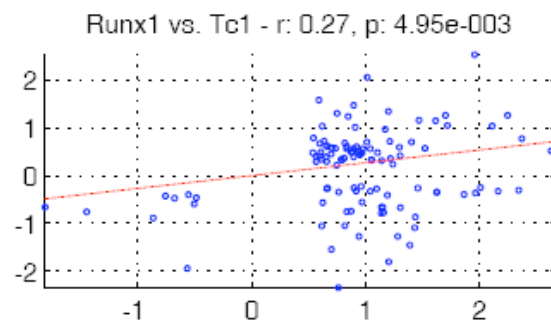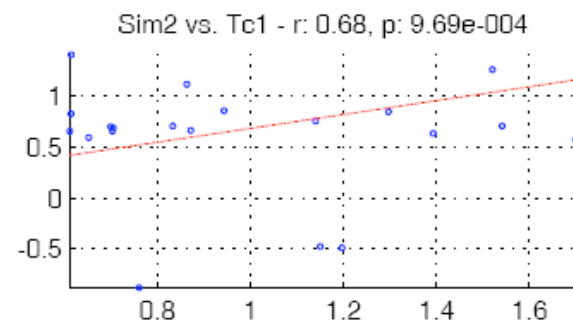

Supplement: Additional file 17 — Comparison of overexpression experiments with the transcriptional response of the transchromosomic tc1 mouse line. X-Y graphs comparing the transcriptional response of Tc1 with the response obtained in the individual overexpression experiments. Each dot represents a gene whose expression was statistically significant in both the Tc1 and the indicated overexpression experiment. The x axis corresponds to the log of the Tc1 ratio (trisomic versus wild type), and the y axis corresponds to the log of the ratio in the overexpression experiment (induced versus non-induced clone). The ratio of same-sign over total dots is reported for each graph. [file gb-2010-11-6-r64-S17.PDF]
