# Supplementary material for: A mouse embryonic stem cell bank for inducible overexpression of human chromosome 21 genes
Source: Genome Biol. 2010 Jun 22;11(6):R64. doi: 10.1186/gb-2010-11-6-r64 (PMC2911112; doi:10.1186/gb-2010-11-6-r64)
Supplement: Additional file 2 — List of 32 genes overexpressed in mouse ES cells. In this table we list the 32 genes selected to be integrated in the Rosa26 locus and overexpressed using the Tet-off system in mES cells. [file gb-2010-11-6-r64-S2.DOC]

**List of 32 genes overexpressed in mouse ES cells**

| **Official Gene** | **Official Gene** | **Molecular** | **Refseq** | **mouse Chromosomal** |
| --- | --- | --- | --- | --- |
| **Symbol** | **Name** | **Function** | **ID (#)** | **location** |
| ***1810007M14Rik*** | RIKEN cDNA 1810007M14 gene | Transcription factor activity | NM_016631 | chr16: 91014282-91044788 |
| ***Aire*** | Autoimmune regulator | Transcription factor activity | NM_009646.1 | chr10: 77492776-77506325 |
| ***Atp5j*** | ATP synthase, H+ transporting, mitochondrial F0 complex, subunit F | Hydrogen ion transmembrane transporter activity | NM_016755.2 | chr16: 84828111-84835819 |
| ***Atp5o*** | ATP synthase, H+ transporting, mitochondrial F1 complex, O subunit | Hydrogen ion transmembrane transporter activity | NM_138597.2 | chr16: 91925468-91931808 |
| ***Bach1*** | BTB and CNC homology 1 | Transcription factor activity | NM_007520.2 | chr16: 87699199-87733591 |
| ***Cct8*** | Chaperonin subunit 8 (theta) | Molecular chaperone | NM_009840.3 | chr16: 87484121-87496085 |
| ***Cstb*** | Cystatin B | Cysteine-type endopeptidase inhibitor activity | NM_007793.3 | chr10: 77888415-77890364 |
| ***Dnmt3l*** | DNA (cytosine-5-)-methyltransferase 3-like | DNA methyltransferase activity | NM_001081695.1 | chr10: 77512587-77526360 |
| ***Dscr1 (Rcan1)*** | Regulator of calcineurin 1 | Transcriptional activator | NM_019466 | chr16: 92392200-92466370 |
| ***Dscr2 (Psmg1)*** | Down syndrome critical region homolog 2 (human) | Molecular chaperone | NM_019537.2 | chr16: 96201561-96212524 |
| ***DYRK1A*** | Dual-specificity tyrosine-(Y)-phosphorylation regulated kinase 1a | Protein serine/threonine kinase activity | NM_130436.2 | chr16: 94791617-94916674 |
| ***Erg*** | Avian erythroblastosis virus E-26 (v-ets) oncogene related | Transcription factor activity | NM_133659.2 | chr16: 95580776-95808200 |
| ***Ets2*** | v-ets erythroblastosis virus E26 oncogene homolog 2 | Transcription factor activity | NM_011809.2 | chr16: 95924153-95942652 |
| ***Gabpa*** | GA binding protein transcription factor, alpha subunit | Transcription factor activity | NM_008065 | chr16: 84835170-84864021 |
| ***Gart*** | phosphoribosylglycinamide formyltransferase | Transferase activity | NM_010256.2 | chr16: 91621649-91647191 |
| ***Hunk*** | Hormonally upregulated Neu-associated kinase | Protein serine/threonine kinase activity | NM_015755.2 | chr16: 90386258-90499794 |
| ***Morc3*** | Microrchidia 3 | Zinc ion binding activity | NM_001045529.2 | chr16: 93832529-93876317 |
| ***Mrpl39*** | Mitocondrial ribo-protein L39 | Ribosomal protein | NM_017404.3 | chr16: 84717821-84735987 |
| ***Nrip1*** | Nuclear receptor interacting protein 1 | Transcription factor activity | NM_173440.2 | chr16: 76287645-76374072 |
| ***Olig1*** | Oligodendrocyte transcription factor 1 | Transcription factor activity | NM_016968 | chr16: 91270014-91272184 |
| ***Olig2*** | Oligodendrocyte lineage transcription factor 2 | Transcription factor activity | NM_016967.2 | chr16: 91270017-91272174 |
| ***Pdxk*** | Pyridoxal (pyridoxine, vitamin B6) kinase | Kinase protein activity | NM_172134.2 | chr10: 77903351-77927668 |
| ***Pfkl*** | Phosphofructokinase, liver, B-type | Kinase protein activity | NM_008826.4 | chr10: 77450654-77472495 |
| ***Pknox1*** | Pbx/knotted 1 homeobox | Transcription factor activity | NM_016670 | chr17: 31701746-31742991 |
| ***Pttg1ip*** | Pituitary tumor-transforming 1 interacting protein | Protein import into nucleus activity | NM_145925.2 | chr10: 77044498-77061476 |
| ***Ripk4*** | Receptor-interacting serine-threonine kinase 4 | Protein serine/threonine kinase activity | NM_023663.6 | chr16: 97963540-97985324 |
| ***Rrp1*** | Ribosomal RNA processing 1 homolog (S. cerevisiae) | rRNA processing activity | NM_010925.2 | chr17: 32173107-32197547 |
| ***Runx1*** | Runt-related transcription factor 1 | Transcription factor activity | NM_001111021.1 | chr16: 92602140-92826311 |
| ***Sim2*** | Single-minded homolog 2 (Drosophila) | Transcription factor activity | NM_011377.2 | chr16: 94086061-94348638 |
| ***SNF1LK*** | SNF1-like kinase | Protein serine/threonine kinase activity | NM_173354.3 | chr17: 31981193-31992737 |
| ***Sod1*** | Superoxide dismutase 1, soluble | Oxidoreductase activity | NM_011434.1 | chr16: 90221039-90226574 |
| ***ZFP295*** | Zinc finger protein 295 | Transcription factor activity | NM_001098403.1 | chr16: 98168599-98183786 |

**#** The Refseq ID is referred to the transcript isoform cloned
