# Supplementary material for: A mouse embryonic stem cell bank for inducible overexpression of human chromosome 21 genes
Source: Genome Biol. 2010 Jun 22;11(6):R64. doi: 10.1186/gb-2010-11-6-r64 (PMC2911112; doi:10.1186/gb-2010-11-6-r64)
Supplement: Additional file 3 — Time course of induction of three clones (biological replicates) selected for each gene. In this table we report the time course of the induction of mES clones that overexpress the 32 ORFs. For each gene, three drug-resistant mES biological replicates, whose names are indicated in the specific column, were selected to be tested for their sensitivity to Tc removal from the medium. [file gb-2010-11-6-r64-S3.DOC]

**Time course of induction of three clones (biological replicates) selected for each gene**

| **Official Gene** | **Clone** | **2^-dCt** |  |  |  |  |
| --- | --- | --- | --- | --- | --- | --- |
| **Symbol** | **Name** | **0hrs** | **17hrs** | **24hrs** | **39hrs** | **48hrs** |
| ***1810007M14Rik*** | **A1** | 0,011 |  | 0,046 |  |  |
|  | **A2** | 0,014 |  | 0,032 |  |  |
|  | **A3** | 6,60E-03 |  | 0,027 |  |  |
| ***Aire*** | **A5** | 9,70E-03 | 0,554 | 0,45 | 0,341 | 0,307 |
|  | **C1** | 1,30E-03 | 0,318 | 0,225 | 0,287 | 0,277 |
|  | **C2** | 5,20E-04 | 0,038 | 0,047 | 0,108 | 0,164 |
| ***Atp5j*** | **A4** | 0,587 | 1,447 | 0,87 | 0,406 | 1,175 |
|  | **C1** | 0,707 | 1,319 | 0,587 | 0,757 | 0,954 |
|  | **C5** | 0,378 | 1,231 | 0,445 | 0,615 | 0,954 |
| ***Atp5o*** | **A2** | 0,189 | 1 | 0,561 | 0,561 | 1,175 |
|  | **A5** | 0,106 | 0,425 | 0,445 | 0,601 | 0,031 |
|  | **B3** | 0,103 | 0,601 | 0,353 | 0,488 | 0,406 |
| ***Bach1*** | **B1** | 4,30E-03 |  | 0,212 |  |  |
|  | **B2** | 3,20E-03 |  | 0,227 |  |  |
|  | **B3** | 3,90E-03 |  | 0,329 |  |  |
| ***Cct8*** | **A2** | 0,274 | 0,5 | 0,723 | 0,69 | 0,601 |
|  | **B7** | 0,15 | 0,425 | 0,523 | 0,396 | 0,561 |
|  | **C1** | 0,189 | 0,267 | 0,307 | 0,37 | 0,406 |
| ***Cstb*** | **A1** | 0,029 | 0,238 | 0,157 | 0,415 | 0,361 |
|  | **B4** | 0,016 | 0,062 | 0,055 | 0,106 | 0,103 |
|  | **C7** | 0,02 | 0,18 | 0,116 | 0,267 | 0,267 |
| ***Dnmt3l*** | **A1** | 9,80E-03 | 0,023 | 0,031 | 0,406 | 0,28 |
|  | **A8** | 0,09 | 0,082 | 0,255 | 0,25 | 0,261 |
|  | **C2** | 0,065 | 0,203 | 0,255 | 9,2512E-05 | 1,50E-04 |
| ***Dscr1 (Rcan1)*** | **F4** | 1,99E-03 |  | 0,244 |  |  |
|  | **E1** | 4,59E-03 |  | 0,133 |  |  |
|  | **E4** | 3,73E-03 |  | 0,227 |  |  |
| ***Dscr2 (Psmg1)*** | **A4** | 0,018 | 0,244 | 0,193 | 0,217 | 0,378 |
|  | **A7** | 0,015 | 0,207 | 0,322 | 0,267 | 0,523 |
|  | **B3** | 0,023 | 0,18 | 0,314 | 0,3 | 0,415 |
| ***DYRK1A*** | **A2** | 0,011 | 0,08 | 0,082 | 0,048 |  |
|  | **A3** | 4,90E-03 | 0,051 | 0,032 | 0,088 |  |
|  | **A4** | 5,60E-03 | 0,122 | 0,039 | 0,122 |  |
| ***Erg*** | **A1** | 7,90E-04 |  | 0,099 |  |  |
|  | **A3** | 2,39E-04 |  | 0,11 |  |  |
|  | **A5** | 1,17E-04 |  | 0,055 |  |  |
| ***Ets2*** | **C3** | 1,90E-03 |  | 0,068 |  |  |
|  | **D2** | 5,15E-03 |  | 0,353 |  |  |
|  | **D6** | 2,24E-04 |  | 0,238 |  |  |
| ***Gabpa*** | **B4** | 0,043 |  | 0,161 |  |  |
|  | **G5** | 3,17E-03 |  | 0,084 |  |  |
|  | **G10** | 0,015 |  | 0,08 |  |  |
| ***Gart*** | **A3** | 0,015 | 0,029 | 0,032 | 0,032 | 0,023 |
|  | **B2** | 0,011 | 0,026 | 0,036 | 0,028 | 0,038 |
|  | **B6** | 0,021 | 0,035 | 0,031 | 0,035 | 0,055 |
| ***Hunk*** | **B4** | 3,32E-03 | 0,075 | 0,061 | 0,076 | 0,063 |
|  | **B7** | 2,24E-03 | 0,048 | 0,059 | 0,065 | 0,051 |
|  | **E2** | 2,63E-03 | 0,01 | 0,031 | 0,044 | 0,02 |
| ***Morc3*** | **A3** | 6,91E-04 | 2,76E-03 | 2,40E-03 | 3,10E-03 | 3,02773E-06 |
|  | **A5** | 3,15E-04 | 1,48E-03 | 5,39E-03 | 1,28E-03 | 3,73E-03 |
|  | **C8** | 5,23E-04 | 1,44E-03 | 3,24E-03 | 3,73E-03 | 2,20836E-05 |
| ***Mrpl39*** | **A1** | 0,031 | 0,261 | 0,25 | 0,161 | 0,185 |
|  | **C5** | 0,021 | 0,435 | 0,28 | 0,203 | 0,3 |
|  | **C7** | 0,035 | 0,523 | 0,084 | 0,082 | 2,40311E-06 |
| ***Nrip1*** | **B1** | 2,35E-03 |  | 0,04 |  |  |
|  | **B2** | 2,40E-03 |  | 0,143 |  |  |
|  | **B3** | 0,001096154 |  | 0,043 |  |  |
| ***Olig1*** | **C1** | 1,00E-03 |  | 0,161 |  |  |
|  | **O3** | 1,00E-03 |  | 0,078 |  |  |
|  | **O6** | 1,00E-03 |  | 0,078 |  |  |
| ***Olig2*** | **C2** | 9,2512E-05 | 4,50E-03 | 9,61E-03 | 7,60E-03 |  |
|  | **C4** | 5,96411E-05 | 4,50E-03 | 0,024 | 7,60E-03 |  |
|  | **C7** | 5,07347E-05 | 4,50E-03 | 9,60E-03 | 7,60E-03 |  |
| ***Pdxk*** | **A1** | 0,021 |  |  |  | 0,487 |
|  | **A7** | 0,019 |  |  |  | 0,433 |
|  | **B1** | 0,024 |  |  |  | 0,73 |
| ***Pfkl*** | **D1** | 0,059 |  |  | 1,009 |  |
|  | **D3** | 0,1 |  |  | 0,715 |  |
|  | **D5** | 0,064 |  |  | 0,676 |  |
| ***Pknox1*** | **B7** | 1,99E-03 |  | 0,116 |  |  |
|  | **P3** | 4,20E-03 |  | 0,644 |  |  |
|  | **P6** | 1,95E-03 |  | 0,056 |  |  |
| ***Pttg1ip*** | **A5** | 0,018 | 0,307 | 0,574 | 0,255 | 0,523 |
|  | **B5** | 7,64E-03 | 0,18 | 0,193 | 0,207 | 0,28 |
|  | **C4** | 6,64E-03 | 0,255 | 0,203 | 0,18 | 0,227 |
| ***Ripk4*** | **A4** | 3,17E-03 | 0,061 | 0,078 | 0,02 |  |
|  | **A5** | 3,24E-03 | 0,061 | 0,078 | 0,042 |  |
|  | **A6** | 2,69E-03 | 0,037 | 0,029 | 0,02 |  |
| ***Rrp1*** | **A8** | 0,027 | 6,80E-03 | 0,012 | 0,024 | 0,013 |
|  | **B3** | 0,013 | 0,031 | 0,014 | 0,033 | 0,027 |
|  | **C2** | 0,013 | 0,02 | 0,016 | 0,011 | 0,036 |
| ***Runx1*** | **E7** | 0,012 |  | 0,745 |  |  |
|  | **F3** | 7,22E-03 |  | 0,567 |  |  |
|  | **E6** | 0,011 |  | 0,6 |  |  |
| ***Sim2*** | **A6** | 1,20E-03 | 0,127 | 0,361 | 0,361 | 0,176 |
|  | **A7** | 1,12E-03 | 0,127 | 0,361 | 0,176 | 0,176 |
|  | **B8** | 4,77E-04 | 0,086 | 0,09 | 0,113 | 0,143 |
| ***SNF1LK*** | **A1** | 2,57E-03 | 0,127 | 0,096 | 0,161 |  |
|  | **B2** | 5,61E-04 | 0,127 | 0,063 | 0,113 |  |
|  | **B3** | 1,78E-03 | 0,168 | 0,09 | 0,13 |  |
| ***Sod1*** | **A2** | 1,148 | 1,122 | 0,933 | 0,933 | 1,381 |
|  | **A8** | 0,406 | 0,659 | 1,122 | 1,023 | 1,122 |
|  | **C2** | 1,259 | 2,406 | 1,319 | 2,094 | 1,781 |
| ***ZFP295*** | **A5** | 1,06E-04 | 4,64E-03 | 5,52E-03 | 4,80E-03 | 1,76E-03 |
|  | **A6** | 5,5008E-05 | 7,28E-03 | 9,29E-03 | 6,34E-03 | 6,34E-03 |
|  | **B2** | 4,46803E-05 | 7,15E-04 | 1,24E-03 | 1,48E-03 | 1,76E-03 |

For each gene, 3 drug-resistant mouse ES biological replicates (whose names are indicated in the second column), were selected to be tested for their sensitivity to the Tc remotion from the medium. For each clone we reported all the time points performed for each time course of induction. The expression level of each gene, in the biological replicates grown in the presence of Tc (0 hrs) and induced at different time points, is reported as relative expression (2^-dCt). The sequence of oligonucleotide primer pairs used in q-PCR are shown in Additional file 4.
