# Supplementary material for: A mouse embryonic stem cell bank for inducible overexpression of human chromosome 21 genes
Source: Genome Biol. 2010 Jun 22;11(6):R64. doi: 10.1186/gb-2010-11-6-r64 (PMC2911112; doi:10.1186/gb-2010-11-6-r64)
Supplement: Additional file 4 — Primer pairs used in q-PCR. [file gb-2010-11-6-r64-S4.DOC]

**Primer pairs used in q-PCR**

| 1810007M14Rik-fw: 5'-GCT CTG CTG CCC ACC ATT GT-3' |
| --- |
| 1810007M14Rik-rev: 5'-TGC CAT ACC ACT GAA GAA AA-3' |
| 2210016F16Rik-fw: 5'-GCA GCC TGT AGT CAG CAA ACA T-3' |
| 2210016F16Rik-rev: 5'-CTT ACA GGG ACG AGG CAG AGT T-3' |
| A630072M18Rik-fw: 5'-CCG AGA GGT GGA CGA GGA GGA G-3' |
| A630072M18Rik-rev: 5'-GCG TCT TTG ATT GGT TTG TTC C-3' |
| Acpl2-fw: 5'-CAA GCG TAG CGG AGC ACA GCA T-3' |
| Acpl2-rev: 5'-CTC AGA TAG ATG TCC CTC AGC A-3' |
| Actb-fw: 5'-ACG TTG ACA TCC GTA AAG ACC T-3' |
| Actb-rev: 5'-GCA GTA ATC TCC TTC TGC ATC C-3' |
| Afp-fw: 5'-AAA CAT CCC ACT TCC AGC AC-3' |
| Afp-rev: 5'-CAC ATT GAT GGA AGC GAA AC-3' |
| Aire-fw: 5'-CAG GTG GGG ATG GAA TGC TAC G-3' |
| Aire-rev: 5'-CAG GAC AGC AGG GCG TGG AAG G-3' |
| Amn-fw: 5'-AGA GGA CGT CGT CGT AGC TG-3' |
| Amn-rev: 5'-GAC TCG GAC CCA GCT TGT AA-3' |
| Arhgef15-fw: 5'-GAA GAG GGT CCT ACA GCC TCA A-3' |
| Arhgef15-rev: 5'-AAA GCA TCT GCA CAA GAA TCCA-3' |
| Ascl2-fw: 5'-GCC GCA CCA GAA CTC GTA GCA G-3' |
| Ascl2-rev: 5'-GGA CGG CGG AGT AGC GGG TGT T-3' |
| Atp5j-fw: 5'-GAG GAT CTT CAG GCT CTC CT-3' |
| Atp5j-rev: 5'-CTC TGG GCC AAT ATC AAC AG-3' |
| Atp5o-fw: 5'-GGT TCG GAG CTT CAG TAC AT-3' |
| Atp5o-rev: 5'-CGG CCT TCG ATG CCG TAG AC-3' |
| Aw548124-fw: 5'-CGG AAC CTC TAC AAT GCC TAC A-3' |
| Aw548124-rev: 5'-ACC ACA AAG GCA CGA CAC TGA G-3' |
| Bach1-fw: 5'-TTC CGT CAA CTG CCC TTT TA-3' |
| Bach1-rev: 5'-GCG GCG ATT CTG TTC TTA CT-3' |
| Bhlhb2-fw:-5'-GCG CGC AAC CAC CTC CTA CC-3' |
| Bhlhb2-rev:-5'-TGT AAA CCG CTC TGC AGG GC-3' |
| Brachyury-fw: 5'-GAA CCT CGG ATT CAC ATC GT-3' |
| Brachyury-rev: 5'-TTC TTT GGC ATC AAG GAA GG-3' |
| Cct8-fw: 5'-CGC CTG GAG AAG TTG TTT GTG A-3' |
| Cct8-rev: 5'-AGC CCC AGC GAA CAC CAG AAC G-3' |
| Cebpb-fw: 5'-GGC CCG CGC CGC GCC GCG CGC CC-3' |
| Cebpb-rev: 5'-AGC CCA GGT AGG CGC GCA GGG CG-3' |
| Chac1-fw: 5'-TTC CAT AGG GGC AGC GAC AAG A-3' |
| Chac1-rev: 5'-CTT CCA GGT GCT CAT CTT GTG C-3' |
| Cidea-fw: 5'-CCA TTT CTG TCC CTT TTC C-3' |
| Cidea-rev: 5'-GCA GCC TGC AGG AAC TTA TC-3' |
| Cobl-fw: 5'-GCC TTC TGC GTT CTC AGG TAG T-3' |
| Cobl-rev: 5'-CTT CTG AGA CCC AAC AGC CTT T-3' |
| Cstb-fw: 5'-GTG TGG CGC GCC ATC TGC CAC-3' |
| Cstb-rev: 5'-GTT GAA ACA CCC TCA AGT GCA-3' |
| Dmkn-fw: 5'-TAG GTC AGC TGC ATC AGA GAG G-3' |
| Dmkn-rev: 5'-GGG AGT CAC ACC TTC ATC TTC C-3' |
| Dner-fw: 5'-TGC CCC GCT GCT GCC CGT GCT G-3' |
| Dner-rev: 5'-GAG CGG GGC AGG ATT ATG TCA G-3' |
| Dnmt3l-fw: 5'-ATC TGC CTC TGC TGT GGA ACT C-3' |
| Dnmt3l-rev: 5'-ATC ATC ATC ATA CAG GAA GAG G-3' |
| Dscr1-fw: 5'-TAG AAG ATG CCA CCC CCG T-3' |
| Dscr1-rev: 5'-ATG CAG TTC ATA CTT CTC TC-3' |
| Dscr2-fw: 5'-GGA GCA GAG CAG GCG GGA CAC G-3' |
| Dscr2-rev: 5'-CTG TTG CGT TGC TTC CTA CTG C-3' |
| Dtna-fw: 5'-TCA ACA AGA GGA TGC CAA CCA C-3' |
| Dtna-rev: 5'-CTT CAA AAA CTG CTG TGG GTA G-3' |
| DYRK1A-fw: 5'-ACC GTC GCC AGC CAA ACA TAA G-3' |
| DYRK1A-rev: 5'-CCA TCC ACT TTT CTC CGT TTT T-3' |
| E230002P03Rik-fw: 5'-CTC GGG TTC TCC ACA CAT TCC A-3' |
| E230002P03Rik-rev: 5'-CAG TAT CCT CAG CAC ATC CAA G-3' |
| Egln3-fw: 5'-TCA ACT TCC TCC TGT CCC TCA T-3' |
| Egln3-rev: 5'-GGC ATA GGA GGG CTG GAC TTC A-3' |
| Egr1-fw: 5'-TCG GCT CCT TTC CTC ACT CAC C-3' |
| Egr1-rev: 5'-TTT GGC TGG GAT AAC TCG TCT C-3' |
| Erg-fw: 5'-AAC AAA GGT GGG AAG ATG GT-3' |
| Erg-rev: 5'-CGG AAG TCA GAT GTG GAA GG-3' |
| Ets2-fw: 5'-GAA AAC CCA AGG ACC ACG AC-3' |
| Ets2-rev: 5'-GCT TGA ACT CCC ATC CAT CC-3' |
| Exoc3l-fw: 5'-GGA TGG AGA GGT AGC AGA GTG G-3' |
| Exoc3l-rev: 5'-CAG CAG GAC CGA GAC CGA AGA T-3' |
| F730031o20Rik-fw: 5'-GCC AAC CGC CTC CAA GTC CTC A-3' |
| F730031o20Rik-rev: 5'-CCT CGT CGG TCT GGG AAG CAC A-3' |
| Fbp1-fw: 5'-AAT GAG GGT TAT GCC AAG GAC T-3' |
| Fbp1-rev: 5'-CGA CTG GTG CCT TCT GGT GGA T-3' |
| Gabpa-fw: 5'-TGA CGG CAC CAA GCA CAT TA-3' |
| Gabpa-rev: 5'-CAT TCA TCT GTT GCT CTT GG-3' |
| Gart-fw: 5'-TCG GTA GCG GGG GAA GGG AAC A-3' |
| Gart-rev: 5'-CAG AGG TCA GGT CCC CAA CAA T-3' |
| Gbp1-fw: 5'-GGA GCA GGA ACG GAA AGA GTT A-3' |
| Gbp1-rev: 5'-TCA TTC TGG AAT CCT TGC TTG A-3' |
| Gbp2-fw: 5'-AGA AGT GAC GGG TTT TCC GTT A-3' |
| Gbp2-rev: 5'-CAT AGG AAC CAT CAA CCA GCA G-3' |
| Gbx2-fw: 5'-AGC AGT CTG ACC AGG CAA ATT-3' |
| Gbx2-rev: 5'-AGA CGG CAA AGC CTT CTT GG-3' |
| Gfap-fw: 5'-GAA AAC CGC ATC ACC ATT CC-3' |
| Gfap-rev: 5'-TCG GAT CTG GAG GTT GGA GA-3' |
| Hs6st2-fw: 5'-ACC GTG ACA CTG GGT CTC TC-3' |
| Hs6st2-rev: 5'-AGG GAG ACC TGG CTC TTC TC-3' |
| Hunk-fw: 5'-CAA CTC ATC TCT GCG GTG GAA C-3' |
| Hunk-rev: 5'-GAT TCA TTG CTT TGT CCA CCA T-3' |
| Igfbp4-fw: 5'-CAG GTC TCA CTC TTG GAA GCT G-3' |
| Igfbp4-fw: 5'-CGT GGG TTG CGA GGA GTT GGT G-3' |
| Igfbp4-rev: 5'-GAC CTC TTC ATC ATC CCC ATT C-3' |
| Igfbp4-rev: 5'-TTG CGG TCA CAG TTT GGA ATG G-3' |
| Inhbb-fw: 5'-GTA CAC CTT GAC CCG TACTT C-3' |
| Inhbb-rev: 5'-GAT CAT CAG CTT TGC AGA GAC A-3' |
| Irgm-fw: 5'-CTT TCG GTG CTC CTA CTG ACC-3' |
| Irgm-rev: 5'-CAG CGT CAC TCG GAT CTT ATC A-3' |
| Jam2-fw: 5'-GGT TCT GGC CTT GCT CAG TC-3' |
| Jam2-rev: 5'-GTG GAA GAA GGT GGG ACA GG-3' |
| Kbtbd11-fw: 5'-CTC CGC CTG GTA GCA ATA AAC-3' |
| Kbtbd11-rev: 5'-CTT CAT GAG CGA CCA CTA CCT G-3' |
| Krt79-fw: 5'-TCC AGC ACC TTG TTC TGT TG-3' |
| Krt79-rev: 5'-GGC ATC CAG GAA GTC ACT GT-3' |
| Lbxcor1-fw: 5'-CCA CCA GTT GAG TTC CTG TGA C-3' |
| Lbxcor1-fw: 5'-GGT CCC ACT TCC TTT CCA ATC T-3' |
| Lbxcor1-rev: 5'-ACC ACA GCG GCG AGA GGA GAT G-3' |
| Lbxcor1-rev: 5'-GCC GGA TAA GGA AGA CAA TCA C-3' |
| Ldhb-fw: 5'-CGC CCA CTA CAG TGA TCT TGT T-3' |
| Ldhb-rev: 5'-CAG CCT GCT GAC TTT GCA GT-3' |
| Lefty1-fw: 5'-TCT GGG CAC TGT CGC TGG TTA G-3' |
| Lefty1-rev: 5'-CTC GCC CTG CCC CTT GCC ATC C-3' |
| Luc-fw: 5'-TTT TGA AGC GAA GGT TGT GG-3' |
| Luc-rev: 5'-GGG AAG ACC TGC GAC ACC TG-3' |
| Mal2-fw: 5'-CCG CTG CTT CCA GTA AAA AC-3' |
| Mal2-rev: 5'-GAT GGG TCA TGT TTG TGT CG-3' |
| Manba-fw: 5'-TCG TTT GTC TGC TGT GTT TTC A-3' |
| Manba-rev: 5'-TGC TCA TCT GGA TTA CCT CAC G-3' |
| Mettl8-fw: 5'-AAA TTC CCT CAA CAG CCA ATT A-3' |
| Mettl8-rev: 5'-GGA TCA CAT GCA GTG GTC TAA A-3' |
| Mgst1-fw: 5'-AGG GAG AGA ATG CCA AGA AGT T-3' |
| Mgst1-rev: 5'-GAG GAA GGG GAG TCA AGT AAG C-3' |
| Mical1-fw: 5'-CGG ACT TCG GGC TGC TGT GGA G-3' |
| Mical1-rev: 5'-ATC TCC ACC CCC AAC AGT AAG G-3' |
| Morc3-fw: 5'-GGG TAA GAA GGG GAC AAG AAT C-3' |
| Morc3-rev: 5'-AGC CTT GAT GAG TCT GTT TTT G-3' |
| Mrpl39-fw: 5'-CCT GAC CCA AAA GAA GTG AAT A-3' |
| Mrpl39-rev:5'-TGA CTA TTC TCT CGG GGT TCT G-3' |
| Msc-fw: 5'-CCA CAA TCC ATC TAA CTG CCC-3' |
| Msc-rev: 5'-ACC GCT ACG AGG ACA GCT ATG T-3' |
| Myl9-fw: 5'-GAG AGC CAA GGC CAA GAC CA-3' |
| Myl9-rev: 5'-CCG TGT TTG AGG ATG CGA GTG A-3' |
| Myo1f-fw: 5'-TCC TCT CAC AGA AGC CAC TGA C-3' |
| Myo1f-rev: 5'-CTA GAT GAT GTG TGT GCC ACG A-3' |
| Ndufa412-fw: 5'-GTC TAG GGA CCC GCT TCT AC-3' |
| Ndufa412-rev: 5'-AGT CGG TTG AAA CGG CAA GG-3' |
| Nrip1-fw: 5'-AGT CAT CCC ATC CCG CAG TG-3' |
| Nrip1-rev: 5'-CCT TCT GCC CAT TCT CTT GC-3' |
| Nt5e-fw: 5'-GCA AAT ACC TGG GCT ACC TGA A-3' |
| Nt5e-rev: 5'-TCT GGG TGT CTG AGG TTG TTG T-3' |
| Oct3/4-fw: 5'-GGT GGA GGA AGC CGA CAA CAA T-3' |
| Oct3/4-rev: 5'-GGG AGG GCT TCG GGC ACT TCA G-3' |
| Olig1-fw: 5'-TCT TCC ACC GCA TCC CTT CT-3' |
| Olig1-rev: 5'-CGA GCA GCA GCG TGG CAA TC-3' |
| Olig2-fw: 5'-CTG GGC GGC GGT GGC TTC AA-3' |
| Olig2-rev: 5'-CTC GCT CAC CAG TCG CTT CA-3' |
| Pcx-fw: 5'-GAC AAG CAC GGC AAG CAC TAC T-3' |
| Pcx-rev: 5'-TAC ACC TCG GAC ACG GAA CTC G-3' |
| Pdgfc-fw: 5'-TGA GCA GGT CCA ATG ACA AA-3' |
| Pdgfc-rev: 5'-GTG CCA GGA AAG CAG ACT TC-3' |
| Pdgfrb-fw: 5'-ACT ATG CGA GCC TTC CAC GAG-3' |
| Pdgfrb-rev: 5'-TGG GTG ACA GTT TTC GTG GAC A-3' |
| Pdxk-fw: 5'-GGG GAC CTC TTC GCT GCC ATG C-3' |
| Pdxk-rev: 5'-CCA CCT TGA GAT TGT CCG GGT GC-3' |
| Pfkl-fw: 5'-GAG AAG CTA CGT GTC TAC C-3' |
| Pfkl-rev: 5'-TTC TGT GAC CGG ACT GAA GGC C-3' |
| Pim2-fw: 5'-GGG AGG CTT TGG CAC CGT CTT C-3' |
| Pim2-rev: 5'-CAG CTT TCA CCC AGC GGC CCC-3' |
| Pitx2-fw: 5'-AGA GAA ACC GCT ACC CAG ACA T-3' |
| Pitx2-rev: 5'-GGG CGG GGA AAA CAT ACT CTG A-3' |
| Pknox1-fw: 5'-CAT CCA CTA TTT CCG TTG CT-3' |
| Pknox1-rev: 5'-TCT CGC TGT TCA TTT TTG TC-3' |
| Pla2g7-fw: 5'-TTG GCA TTG GCT TGG CAT CTA A-3' |
| Pla2g7-rev: 5'-GGC AGA GTT GAT AAA GAG GAG A-3' |
| Prmt8-fw: 5'-AGT TCC GGT ACG TGA GTG TCC T-3' |
| Prmt8-rev: 5'-TGT CCA CCA TGT ATC CAC ACA G-3' |
| Pttg1ip-fw: 5'-AAG CCA GAC AAG AGC GAT GAG C-3' |
| Pttg1ip-rev: 5'-TCT CAT ACG GGT TTT GTT CTT T-3' |
| Rab3il1-fw: 5'-CAT CGG AAA AGC AGT TGA AGG A-3' |
| Rab3il1-rev: 5'-ACC TCC TTG CCT TCT TTG TCA G-3' |
| Ripk4-fw: 5'-GCT CTC GGG GGT GTC CTC AGT G-3' |
| Ripk4-rev: 5'-TGT AGA GCC CTT CCT GTT GGT C-3' |
| Rrp1-fw: 5'-CGG TGG TGA CAA TGA TGA AGG T-3' |
| Rrp1-rev: 5'-CAT CCC ACG CCT GTT CCT TGT C-3' |
| Runx1-fw: 5'-GCA CCG ACA GCC CCA ACT TCC T-3' |
| Runx1-rev: 5'-AGC GAT GGG CAG GGT CTT GTT G-3' |
| Serpinb6c-fw: 5'-TGA GTT ATC TGG ATC GCA GTG G-3' |
| Serpinb6c-rev: 5'-GCT CAT CTT GCT ATC TCC AGG C-3' |
| Serpine2-fw: 5'-TTG GTA AAG TGC TGA AGA AGA T-3' |
| Serpine2-rev: 5'-AAT GTC CGT TTC TTT GTG CTC T-3' |
| Serpinf1-fw: 5'-TTA CTG CCC CTG AGA AGA ACC T-3' |
| Serpinf1-rev: 5'-ACG CCA AGG AGA AGG ATG CTG A-3' |
| Sim2-fw: 5'-GAG AAG GAA AAT GGC GAG TT-3' |
| Sim2-rev: 5'-CCT GGG ACA AGC CTA AAT GG-3' |
| Slc47a1-fw: 5'-AGC GTG TCA CAA GCA GAA GA-3' |
| Slc47a1-rev: 5'-CCC AGC TGA TGA TGT TCC TAA-3' |
| Slc6a1-fw: 5'-TGC GGG TGT TCC TCT CTT CCT T-3' |
| Slc6a1-rev: 5'-TGG AGA AGC AGC GGT CAG TGT T-3' |
| Slco4a1-fw: 5'-TTC CTC ATC GCC ATC CCC ATC C-3' |
| Slco4a1-rev: 5'-GAA CTT GGG ACC GAA TGT GGA C-3' |
| SNF1LK-fw: 5'-TGA CTT CCA ACG GGC ACC TGA G-3' |
| SNF1LK-rev: 5'-AAG GGG AGA GAA CCG CAG ACC A-3' |
| Sod1-fw: 5'-GGT GTG CGT GCT GAA GGG CG-3' |
| Sod1-rev: 5'-TCT TCA TCC GCC GGG CCA CC-3' |
| Sox18-fw: 5'-CCG GTA CTT GTA GTT GGG ATG G-3' |
| Sox18-rev: 5'-ACA AAA TCC GGA TCT GCA CAA-3' |
| Syncrip-fw: 5'-TGC CAA CAC GGT AAC AGA AGA A-3' |
| Syncrip-rev: 5'-TGC TCC TCT ACC ACC CCT TCC T-3' |
| Ttn-fw: 5'-GCC GAA GCA TAC CCT GAA GAC T-3' |
| Ttn-rev: 5'-GGA CTT GGG CTT AGG AGG TAT C-3' |
| Vegfc-fw: 5'-GGC ATC GGC ACA TGT AGT TA-3' |
| Vegfc-rev: 5'-TTT GCC AAT CAC ACT TCC TG-3' |
| YFP-fw: 5'-AGC TGA CCC TGA AGC TGA TCT-3' |
| YFP-rev: 5'-ACGTTGTGGCTGTTGTAGTTGT-3' |
| ZFP295-fw: 5'-CAC ACG CCA TTT CTC TCC TAA G-3' |
| ZFP295-rev: 5'-GGC TGC TCT TCT CAA CAA ATA G-3' |
