# Supplementary material for: A mouse embryonic stem cell bank for inducible overexpression of human chromosome 21 genes
Source: Genome Biol. 2010 Jun 22;11(6):R64. doi: 10.1186/gb-2010-11-6-r64 (PMC2911112; doi:10.1186/gb-2010-11-6-r64)
Supplement: Additional file 5 — Comparison of relative expression levels for 20 genes in the EB3 parental cell line and in the inducible clones at 0 hours of induction by multiple statistical t-tests. In this table we show the comparison of the relative expression of 20 genes (the 13 transcription factors, the single transcriptional activator and the 6 kinases) in the EB3 cell line versus the corresponding transgenic inducible clones (in the biological replicates) grown in the presence of Tc (0 hours of induction). [file gb-2010-11-6-r64-S5.DOC]

**Comparison of relative expression levels for 20 genes in the EB3 parental cell line and in the inducible clones at 0hrs of induction by multiple statistical t-tests**

| **Official Gene** | **Clone** | **2^-dCt** | **2^-dCt** | **p-value** | **FDR** |
| --- | --- | --- | --- | --- | --- |
| **Symbol** | **Name** | **0hrs of induction** | **Eb3 (A2;B2;C2)** | **(t-test)** |  |
| ***Bach1*** | **B1** | 4,30E-03 | 0,014936646 | 0,04008991 | 0,113588078 |
|  | **B2** | 3,20E-03 | 0,017824433 |  |  |
|  | **B3** | 3,90E-03 | 0,010166733 |  |  |
| ***Dscr1 (Rcan1)*** | **F4** | 1,99E-03 | 0,000620191 | 0,065521745 | 0,139233709 |
|  | **E1** | 4,59E-03 | 0,000729907 |  |  |
|  | **E4** | 3,73E-03 | 0,000534323 |  |  |
| ***Erg*** | **A1** | 7,90E-04 | 1,65823E-05 | 0,217794773 | 0,231406947 |
|  | **A3** | 2,39E-04 | 1,21811E-05 |  |  |
|  | **A5** | 1,17E-04 | 1,04946E-05 |  |  |
| ***Ets2*** | **C3** | 1,90E-03 | 0,005336095 | 0,161485873 | 0,196089989 |
|  | **D2** | 5,15E-03 | 0,004910208 |  |  |
|  | **D6** | 2,24E-04 | 0,00565993 |  |  |
| ***Gabpa*** | **B4** | 0,043 | 0,070560275 | 0,07373687 | 0,125352679 |
|  | **G5** | 3,17E-03 | 0,060371021 |  |  |
|  | **G10** | 0,015 | 0,040526236 |  |  |
| ***Nrip1*** | **B1** | 2,35E-03 | 0,004143311 | 0,081198915 | 0,115031796 |
|  | **B2** | 2,40E-03 | 0,004726539 |  |  |
|  | **B3** | 0,001096154 | 0,002586111 |  |  |
| ***Olig1*** | **C1** | 1,00E-03 | 6,89065E-05 | 0,003100726 | 0,052712342 |
|  | **O3** | 1,00E-03 | 4,40652E-05 |  |  |
|  | **O6** | 1,00E-03 | 1,69307E-05 |  |  |
| ***Olig2*** | **C2** | 9,2512E-05 | 1,66844E-07 | 0,033661385 | 0,114448709 |
|  | **C4** | 5,96411E-05 | 1,70941E-07 |  |  |
|  | **C7** | 5,07347E-05 | 1,00591E-07 |  |  |
| ***Pknox1*** | **B7** | 1,99E-03 | 0,003436129 | 0,196866831 | 0,223115742 |
|  | **P3** | 4,20E-03 | 0,00439476 |  |  |
|  | **P6** | 1,95E-03 | 0,004693891 |  |  |
| ***1810007M14Rik*** | **A1** | 0,011 | 0,004364403 | 0,080700056 | 0,124718268 |
|  | **A2** | 0,014 | 0,003734161 |  |  |
|  | **A3** | 6,60E-03 | 0,002604099 |  |  |
| ***Runx1*** | **E7** | 0,012 | 0,000592869 | 0,026199856 | 0,111349387 |
|  | **F3** | 7,22E-03 | 0,000491678 |  |  |
|  | **E6** | 0,011 | 0,000356206 |  |  |
| ***Aire*** | **A5** | 9,70E-03 | 0,000615907 | 0,337762782 | 0,337762782 |
|  | **C1** | 1,30E-03 | 0,000624505 |  |  |
|  | **C2** | 5,20E-04 | 0,000714886 |  |  |
| ***Sim2*** | **A6** | 1,20E-03 | 1,836E-06 | 0,058434727 | 0,141912908 |
|  | **A7** | 1,12E-03 | 7,44653E-06 |  |  |
|  | **B8** | 4,77E-04 | 5,86271E-06 |  |  |
| ***Ripk4*** | **A4** | 3,17E-03 | 0,007894152 | 0,02013211 | 0,114081958 |
|  | **A5** | 3,24E-03 | 0,006045176 |  |  |
|  | **A6** | 2,69E-03 | 0,006108357 |  |  |
| ***Hunk*** | **B4** | 3,32E-03 | 0,005154328 | 0,073696033 | 0,139203617 |
|  | **B7** | 2,24E-03 | 0,003670011 |  |  |
|  | **E2** | 2,63E-03 | 0,003760135 |  |  |
| ***Pdxk**** | **A1** | 0,021 | 0,00056675 | 0,004545047 | 0,038632896 |
|  | **A7** | 0,019 | 0,000324386 |  |  |
|  | **B1** | 0,024 | 0,000372622 |  |  |
| ***Pfkl*** | **D1** | 0,059 | 0,054978067 | 0,146701524 | 0,191840454 |
|  | **D3** | 0,1 | 0,038606832 |  |  |
|  | **D5** | 0,064 | 0,037162722 |  |  |

Multiple statistical t-test was used to compare the relative expression (2^-dCt) of 20 genes in the EB3 cell line and in the inducible clones at 0hrs of induction. For each gene we report the “Official Gene Symbol” and the names of the 3 drug-resistant mouse ES biological replicates already tested for their sensitivity to the Tc removal from the medium (refer to Additional file 3). The threshold for statistical significance chosen was FDR<0,05. Only in the case of the transcription factor *Pdxk* (highlighted with an asterisk) there is a statistically significant (corrected p-value (FDR)=0.04), albeit mild, leakiness.
