# Supplementary material for: A mouse embryonic stem cell bank for inducible overexpression of human chromosome 21 genes
Source: Genome Biol. 2010 Jun 22;11(6):R64. doi: 10.1186/gb-2010-11-6-r64 (PMC2911112; doi:10.1186/gb-2010-11-6-r64)
Supplement: Additional file 13 — Summary of q-PCR validation of microarray data. In this table we show the validation by q-PCR of the differential expression of a subset of the most up-regulated and down-regulated genes detected by microarray analysis of the seven effective genes, as ranked by differential expression ratio. [file gb-2010-11-6-r64-S13.DOC]

**Summary of q-PCR validation of microarray data**

| ***Runx1* down-regulated genes** | | |  |  |
| --- | --- | --- | --- | --- |
| **Probe Set** | **Public** | **Gene** | **Signed ratio** | **Signed ratio** |
| **ID** | **ID** | **Symbol** | **(array)** | **(q-PCR)** |
| 1451141_at | BC004636 | *Mettl8* | -9,07 | -14,00 |
| 1426858_at | BB253137 | *Inhbb* | -8,57 | -9,00 |
| 1420337_at | L39770 | *Gbx2* | -8,06 | -6,00 |
| 1419123_a_at | NM_019971 | *Pdgfc* | -8,01 | -9,00 |
| 1419417_at | NM_009506 | *Vegfc* | -7,99 | -12,00 |
| ***Runx1* up-regulated genes** | |  |  |  |
| **Probe Set** | **Public** | **Gene** | **Signed ratio** | **Signed ratio** |
| **ID** | **ID** | **Symbol** | **(array)** | **(q-PCR)** |
| 1427352_at | BC019155 | *Krt79* | 45,15 | 198,00 |
| 1417956_at | NM_007702 | *Cidea* | 21,77 | 78,00 |
| 1450047_at | AW536432 | *Hs6st2* | 11,82 | 25,00 |
| 1427042_at | BB127697 | *Mal2* | 9,34 | 32,00 |
| 1417920_at | NM_033603 | *Amn* | 7,90 | 6,00 |
|  | | |  |  |
| ***Nrip1* down-regulated genes** | | |  |  |
| **Probe Set** | **Public** | **Gene** | **Signed ratio** | **Signed ratio** |
| **ID** | **ID** | **Symbol** | **(array)** | **(q-PCR)** |
| 1418395_at | NM_026183 | *Slc47a1* | -3,15 | -3,20 |
| 1436568_at | AU016127 | *Jam2* | -2,95 | -2,70 |
| 1434499_a_at | AV219418 | *Ldhb* | -2,84 | -3,50 |
| 1434917_at | BQ173923 | *Cobl* | -2,83 | -3,50 |
| 1450626_at | NM_027288 | *Manba* | -2,83 | -1,10 |
| ***Nrip1* up-regulated genes** | |  |  |  |
| **Probe Set** | **Public** | **Gene** | **Signed ratio** | **Signed ratio** |
| **ID** | **ID** | **Symbol** | **(array)** | **(q-PCR)** |
| 1420549_at | NM_010259 | *Gbp1* | 27,39 | 143,00 |
| 1435906_x_at | BE197524 | *Gbp2* | 19,77 | 137,00 |
| 1424797_a_at | U80011 | *Pitx2* | 13,14 | 3,50 |
| 1418825_at | NM_008326 | *Irgm* | 7,34 | 9,20 |
| 1437880_at | AV273001 | *Lbxcor1* | 6,48 | 8,10 |
|  | |  |  |  |
| ***Erg* down-regulated genes** | |  |  |  |
| **Probe Set** | **Public** | **Gene** | **Signed ratio** | **Signed ratio** |
| **ID** | **ID** | **Symbol** | **(array)** | **(q-PCR)** |
| 1451594_s_at | AF425084 | *Serpinb6c* | -6,03 | -2,70 |
| 1428331_at | BB547211 | *2210016F16Rik* | -4,56 | -3,40 |
| 1435204_at | BG065273 | *Prmt8* | -4,54 | -8,30 |
| 1418417_at | NM_010827 | *Msc* | -4,38 | -6,00 |
| 1429524_at | AK021181 | *Myo1f* | -4,31 | 1,40 |
| ***Erg* up-regulated genes** | |  |  |  |
| **Probe Set** | **Public** | **Gene** | **Signed ratio** | **Signed ratio** |
| **ID** | **ID** | **Symbol** | **(array)** | **(q-PCR)** |
| 1437405_a_at | BB787243 | *Igfbp4* | 24,52 | 23,00 |
| 1449135_at | NM_009236 | *Sox18* | 16,73 | 181,00 |
| 1428781_at | BI452905 | *Dmkn* | 16,07 | 31,00 |
| 1455522_at | AW492648 | *Arhgef15* | 11,24 | 17,00 |
|  |  |  |  |  |
| ***Sim2* down-regulated genes** | | |  |  |
| **Probe Set** | **Public** | **Gene** | **Signed ratio** | **Signed ratio** |
| **ID** | **ID** | **Symbol** | **(array)** | **(q-PCR)** |
| 1422768_at | BG920261 | *Syncrip* | -2,86 | -0,45 |
| 1436536_at | AW108488 | *Exoc3l* | -2,70 | -1,12 |
| 1427081_at | BB246700 | *A630072M18Rik* | -2,40 | -2,46 |
| 1452142_at | M92378 | *Slc6a1* | -2,38 | -1,62 |
| 1452670_at | AK007972 | *Myl9* | -2,33 | -1,52 |
| ***Sim2* up-regulated genes** | |  |  |  |
| **Probe Set** | **Public** | **Gene** | **Signed ratio** | **Signed ratio** |
| **ID** | **ID** | **Symbol** | **(array)** | **(q-PCR)** |
| 1428391_at | AK004767 | *Rab3il1* | 4,28 | 5,28 |
| 1456379_x_at | BB038556 | *Dner* | 3,99 | 3,56 |
| 1447173_at | BB704012 | *E230002P03Rik* | 3,73 | 3,32 |
| 1456735_x_at | BB458645 | *Acpl2* | 3,66 | 7,29 |
| 1436970_a_at | AA499047 | *Pdgfrb* | 3,66 | 1,45 |
|  | | |  |  |
| ***Olig2* down-regulated genes** | | |  |  |
| **Probe Set** | **Public** | **Gene** | **Signed ratio** | **Signed ratio** |
| **ID** | **ID** | **Symbol** | **(array)** | **(q-PCR)** |
| 1438160_x_at | AV348121 | *Slco4a1* | -4,90 | -4,19 |
| 1448470_at | NM_019395 | *Fbp1* | -3,13 | -9,40 |
| 1416759_at | NM_138315 | *Mical1* | -2,88 | -2,30 |
| 1444426_at | BB327547 | *F730031O20Rik* | -2,71 | -3,91 |
| 1416383_a_at | NM_008797 | *Pcx* | -2,70 | -4,09 |
| ***Olig2* up-regulatd genes** | |  |  |  |
| **Probe Set** | **Public** | **Gene** | **Signed ratio** | **Signed ratio** |
| **ID** | **ID** | **Symbol** | **(array)** | **(q-PCR)** |
| 1428547_at | AV273591 | *Nt5e* | 5,20 | 7,64 |
| 1423233_at | BB831146 | *Cebpd* | 2,86 | 0,89 |
| 1432018_at | AK010738 | *Ascl2* | 2,64 | 2,35 |
| 1417216_at | NM_138606 | *Pim2* | 2,51 | 3,65 |
|  | |  |  |  |
| ***Aire* down-regulatd genes** | |  |  |  |
| **Probe Set** | **Public** | **Gene** | **Signed ratio** | **Signed ratio** |
| **ID** | **ID** | **Symbol** | **(array)** | **(q-PCR)** |
| 1437880_at | AV273001 | *Lbxcor1* | 0,23 | -1,66 |
| 1417065_at | NM_007913 | *Egr1* | 0,23 | -18,81 |
| 1454838_s_at | BB323985 | *AW548124* | 0,37 | -1,74 |
| 1451382_at | BC025169 | *Chac1* | 0,44 | -1,29 |
| 1430700_a_at | AK005158 | *Pla2g7* | 0,44 | -4,49 |
| ***Aire* up-regulatd genes** | |  |  |  |
| **Probe Set** | **Public** | **Gene** | **Signed ratio** | **Signed ratio** |
| **ID** | **ID** | **Symbol** | **(array)** | **(q-PCR)** |
| 1417638_at | NM_010094 | *Lefty1* | 3,66 | 3,82 |
| 1415897_a_at | BI150149 | *Mgst1* | 3,26 | 4,81 |
| 1456069_at | BM117918 | *Dtna* | 2,90 | 3,91 |
| 1416666_at | NM_009255 | *Serpine2* | 2,76 | 7,46 |
| 1447173_at | BB704012 | *E230002P03Rik* | 2,49 | 3,65 |
|  |  |  |  |  |
| ***Pdxk* down-regulatd genes** | |  |  |  |
| **Probe Set** | **Public** | **Gene** | **Signed ratio** | **Signed ratio** |
| **ID** | **ID** | **Symbol** | **(array)** | **(q-PCR)** |
| 1451382_at | BC025169 | *Chac1* | -2,21 | -3,32 |
| 1427445_a_at | BC025840 | *Ttn* | -2,17 | -3,91 |
| 1416168_at | NM_011340 | *Serpinf1* | -2,12 | -2,76 |
| 1452142_at | M92378 | *Slc6a1* | -2,09 | -1,52 |
| ***Pdxk* up-regulatd genes** | |  |  |  |
| **Probe Set** | **Public** | **Gene** | **Signed ratio** | **Signed ratio** |
| **ID** | **ID** | **Symbol** | **(array)** | **(q-PCR)** |
| 1434905_at | BB610230 | *Ndufa4l2* | 6,31 | 22,63 |
| 1418649_at | BB284358 | *Egln3* | 5,75 | 12,13 |
| 1418025_at | NM_011498 | *Bhlhb2* | 4,29 | 7,82 |
| 1423756_s_at | BC019836 | *Igfbp4* | 4,14 | 9,85 |

The values of expression of each gene, in the array and in the q-PCR analysis, is reported as differential expression ratio (signed ratio-array and signed ratio q-PCR, respectively).
