# Supplementary material for: A mouse embryonic stem cell bank for inducible overexpression of human chromosome 21 genes
Source: Genome Biol. 2010 Jun 22;11(6):R64. doi: 10.1186/gb-2010-11-6-r64 (PMC2911112; doi:10.1186/gb-2010-11-6-r64)
Supplement: Additional file 14 — A complete list of significantly enriched GO terms for the seven effective genes. [file gb-2010-11-6-r64-S14.DOC]

**A complete list of significantly enriched Gene Ontology (GO) terms for the seven effective genes**

| **Gene** | **Gene Onthology** | **FDR** | **Fold** |
| --- | --- | --- | --- |
| **Symbol** | **terms** |  | **Enrichment** |
| *Runx1* | monosaccharide metabolic process | 0.0 | 1.8 |
|  | hexose metabolic process | 0.0 | 1.8 |
|  | regulation of cell differentiation | 0.0 | 1.7 |
|  | chordate embryonic development | 0.0 | 1.6 |
|  | embryonic development ending in birth or egg hatching | 0.0 | 1.6 |
|  | anatomical structure formation | 0.0 | 1.6 |
|  | cell migration | 0.0 | 1.5 |
|  | blood vessel morphogenesis | 0.1 | 1.6 |
|  | angiogenesis | 0.1 | 1.6 |
|  | vasculature development | 0.1 | 1.5 |
|  | blood vessel development | 0.2 | 1.5 |
|  | regulation of kinase activity | 0.2 | 1.6 |
|  | skeletal development | 0.2 | 1.5 |
|  | alcohol catabolic process | 0.3 | 2.0 |
|  | monosaccharide catabolic process | 0.5 | 2.0 |
|  | regulation of transferase activity | 0.4 | 1.5 |
|  | positive regulation of cell proliferation | 0.5 | 1.5 |
|  | regulation of myeloid cell differentiation | 0.8 | 2.1 |
|  | cellular carbohydrate catabolic process | 1.4 | 1.8 |
|  | carbohydrate catabolic process | 1.4 | 1.8 |
|  | negative regulation of catalytic activity | 1.5 | 1.9 |
|  | skeletal morphogenesis | 1.7 | 2.5 |
|  | negative regulation of cell differentiation | 1.8 | 1.7 |
|  | positive regulation of cell adhesion | 1.9 | 2.9 |
|  | cell-matrix adhesion | 2.0 | 1.8 |
|  | morphogenesis of a branching structure | 2.1 | 1.8 |
|  | positive regulation of myeloid cell differentiation | 2.4 | 2.7 |
|  | negative regulation of multicellular organism growth | 2.7 | 3.6 |
|  | monosaccharide transport | 2.7 | 3.6 |
|  | negantive regulation of developmental process | 2.8 | 1.6 |
|  | regulation of cell motility | 2.8 | 1.7 |
|  | negative regulation of progression through cell cycle | 2.8 | 1.5 |
|  | hexose transport | 2.9 | 2.3 |
|  | positive regulation of developmental process | 3.1 | 1.7 |
|  | mitochondrion organization and biogenesis | 3.5 | 1.7 |
|  | tetrahydrobiopterin metabolic process | 3.8 | 3.9 |
|  | positive regulation of cell differentiation | 4.0 | 1.8 |
|  | negative regulation of transferase activity | 4.4 | 1.9 |
| *Erg* | angiogenesis | 0.0 | 1.6 |
|  | vasculature development | 0.1 | 1.5 |
|  | actin filament-based process | 0.1 | 1.5 |
|  | blood vessel development | 0.2 | 1.5 |
|  | blood vessel morphogenesis | 0.2 | 1.5 |
|  | negative regulation of transferase activity | 0.2 | 2.1 |
|  | regulation of myeloid cell differentiation | 0.3 | 2.1 |
|  | negative regulation of trascription,DNA-dependent | 0.5 | 1.5 |
|  | positive regulation of developmental process | 0.7 | 1.7 |
|  | regulation of cell differentiation | 0.8 | 1.5 |
|  | regulation of cell size | 0.9 | 1.6 |
|  | negative regulation of catalytic activity | 1.0 | 1.9 |
|  | morphogenesis of an epithelium | 1.2 | 1.6 |
|  | regulation of cell migration | 1.2 | 1.8 |
|  | apoptotic program | 1.3 | 1.9 |
|  | cell growth | 2.3 | 1.5 |
|  | amino acid biosynthetic process | 2.6 | 1.9 |
|  | regulation of cell motility | 2.5 | 1.7 |
|  | regulation of myeloid leukocyte differentiation | 4.0 | 2.3 |
|  | response to UV | 4.4 | 2.2 |
|  | negantive regulation of developmental process | 4.4 | 1.5 |
| *Nrip1* | organ development | 0.0 | 1.9 |
|  | vasculature development | 0.0 | 3.4 |
|  | cell migration | 0.0 | 2.9 |
|  | blood vessel development | 0.0 | 3.2 |
|  | organ morphogenesis | 0.0 | 2.3 |
|  | angiogenesis | 0.1 | 3.6 |
|  | blood vessel morphogenesis | 0.1 | 3.2 |
|  | nervous system development | 0.1 | 2.0 |
|  | anatomical structure formation | 0.5 | 2.9 |
|  | regulation of cell migration | 0.9 | 4.8 |
|  | cell development | 1.0 | 1.6 |
|  | muscle cell differentiation | 2.0 | 4.3 |
|  | regulation of cell motility | 2.2 | 4.2 |
|  | negative regulation of transcription, DNA-dependent | 3.6 | 2.6 |
| *Olig2* | actin filament-based process | 0.0 | 2.3 |
|  | cytoskeleton organization and biogenesis | 0.2 | 1.7 |
|  | organ morphogenesis | 0.5 | 1.6 |
|  | placenta development | 1.3 | 4.3 |
|  | cell morphogenesis | 2.3 | 1.5 |
|  | cellular structure morphogenesis | 2.3 | 1.5 |
|  | respiratory tube development | 2.5 | 2.7 |
|  | morphogenesis of an epithelium | 3.0 | 2.2 |
|  | cell growth | 3.2 | 2.2 |
|  | regulation of cell size | 3.6 | 2.1 |
|  | negative regulation of progression through cell cycle | 3.6 | 2.1 |
| *Pdxk* | hexose metabolic process | 0.0 | 7.2 |
|  | monosaccharide metabolic process | 0.0 | 7.1 |
|  | monosaccharide catabolic process | 0.0 | 10.8 |
|  | alcohol catabolic process | 0.0 | 10.3 |
|  | cellular carbohydrate catabolic process | 0.0 | 8.8 |
|  | cellular carbohydrate metabolic process | 0.0 | 4.1 |
|  | carboxylic acid metabolic process | 0.0 | 2.6 |
|  | nitrogen compund biosynthetic process | 0.3 | 5.0 |
|  | monocarboxylic acid metabolic process | 0.4 | 3.2 |
|  | amino acid biosynthetic process | 0.6 | 7.3 |
|  | cellular macromolecule catabolic process | 2.0 | 2.7 |
|  | induction of programmed cell death | 3.2 | 3.3 |
|  | monosaccharide biosynthetic process | 3.9 | 8.8 |
|  | alcohol biosynthetic process | 4.0 | 8.6 |
| *Aire* | cell morphogenesis | 0.0 | 1.6 |
|  | cellular structure morphogenesis | 0.0 | 1.6 |
|  | regulation of progression through cell cycle | 0.0 | 1.7 |
|  | regulation of cell differentiation | 0.4 | 1.9 |
|  | hexose metabolic process | 0.6 | 2.0 |
|  | monosaccharide metabolic process | 0.6 | 1.9 |
|  | negative regulation of progression through cell cycle | 0.7 | 2.0 |
|  | negative regulation of cell differentiation | 2.4 | 2.1 |
|  | alcohol catabolic process | 2.5 | 2.4 |
|  | cell migration | 4.0 | 1.5 |
| *Sim2* | RNA processing | 3.3 | 1.8 |

The data in this table was collected by a GO analysis performed on the list of differentially expressed genes (reported in Additional files 6 to 12) using the DAVID online tool restricting the output to Biological Process terms of level 4 and 5, with a significance threshold of FDR (false discovery rate) <5% and Fold Enrichment ≥1.5%.

In the table we report the gene symbols, all significant GO terms for the seven effective genes, the FDR and the Fold Enrichment values for each term.
