# Supplementary material for: A mouse embryonic stem cell bank for inducible overexpression of human chromosome 21 genes
Source: Genome Biol. 2010 Jun 22;11(6):R64. doi: 10.1186/gb-2010-11-6-r64 (PMC2911112; doi:10.1186/gb-2010-11-6-r64)
Supplement: Additional file 15 — List of all the mouse orthologs of HSA21 genes sorted according to their basal expression level in mES cells (from the most to the least expressed). [file gb-2010-11-6-r64-S15.DOC]

**List of all the mouse orthologue of HSA21 genes sorted according to their basal expression level in mouse ES cells (from the most to the least expressed)**

| **Gene symbol** | **AVERAGE** | **STDEV** |
| --- | --- | --- |
| *LOC100039571 /// LOC100041163 /// LOC100043755 /// Rpl23a* | 13.9143 | 0.014295578 |
| *Atp5j* | 12.2475 | 0.0873483 |
| *Dnmt3l* | 11.9458 | 0.331459 |
| *LOC100040029 /// LOC100044330 /// LOC629480 /// U2af1* | 11.8845 | 0.24028 |
| *Atp5o /// EG432676 /// LOC100047429* | 11.4232 | 0.948230194 |
| *Dscr2* | 11.1752 | 0.105499 |
| *1500032D16Rik* | 10.9673 | 0.24901 |
| *Btg3 /// EG654432 /// LOC100048453* | 10.9368 | 0.226228 |
| *Mrpl39* | 10.91065 | 0.619920515 |
| *Hmgn1* | 10.841165 | 4.690236028 |
| *Cstb* | 10.63475 | 0.67465058 |
| *Rrp1* | 10.247625 | 0.716688078 |
| *Col18a1* | 10.1186 | 0.089236876 |
| *Mcm3ap* | 9.96575 | 0.357182 |
| *Rrp1b* | 9.806043333 | 0.410051712 |
| *Gart* | 9.792415 | 2.078086699 |
| ***Gabpa*** | **9.701345** | **1.175289252** |
| *Pwp2* | 9.48545 | 0.200232 |
| *Cbr3* | 9.39494 | 0.575998 |
| *Cbr1* | 9.30638 | 0.182542 |
| *2610039C10Rik* | 9.289745 | 0.26230126 |
| *LOC100047915 /// Memo1* | 9.19412 | 0.223715 |
| *Mrps6* | 8.925465 | 0.570034132 |
| *Jam2* | 8.899728 | 0.935738825 |
| ***Pfkl*** | **8.827736667** | **3.258973778** |
| *Donson* | 8.81134 | 0.154938 |
| *Pcnt* | 8.739395 | 0.392366482 |
| *D10Jhu81e /// LOC100046684* | 8.70329 | 0.210799 |
| *Pigp* | 8.53275 | 0.234103 |
| *Morc3* | 8.459263333 | 2.668681211 |
| *Son* | 8.44961 | 1.374365919 |
| ***Hunk /// LOC630567*** | **8.43412** | **0.248903** |
| *Cryzl1* | 8.419786667 | 0.753923345 |
| *Dscr3* | 8.4 | 0.274149 |
| *LOC100041004 /// Tff1* | 8.39229 | 0.202419 |
| ***Aire*** | **8.23065** | **0.580475** |
| *Tmem1* | 8.18632 | 0.287381 |
| *1810008A18Rik* | 8.0636 | 0.256591 |
| ***Dyrk1a*** | **8.02192** | **0.400889375** |
| *1190017O12Rik* | 8.00611 | 0.159652 |
| *Sumo3* | 7.85458 | 6.374878759 |
| *Tmem50b* | 7.83942 | 0.224298 |
| *Pofut2* | 7.8308175 | 1.003876961 |
| *Prmt2* | 7.820335 | 0.078495924 |
| ***Pdxk*** | **7.71487** | **0.242133622** |
| *Hsf2bp* | 7.685475 | 3.223919019 |
| *Cct8* | 7.605265 | 6.554646516 |
| *Rwdd2b* | 7.59637 | 0.158917 |
| ***Pknox1*** | **7.499576667** | **0.55049538** |
| ***Bach1*** | **7.40807** | **1.744446571** |
| *4921511H13Rik* | 7.407845 | 2.687507814 |
| *Ube2g2* | 7.380605 | 1.613709599 |
| ***Rcan1*** | **7.372435** | **0.978459008** |
| *Slc37a1* | 7.32977 | 0.281564 |
| *Pde9a* | 7.32263 | 0.448755 |
| *Cbs* | 7.303645 | 0.955435612 |
| *Ttc3* | 7.289784286 | 2.209430698 |
| *Sfrs15* | 7.28637 | 1.410918445 |
| *1110004E09Rik* | 7.2509925 | 1.075190173 |
| *Pcbp3* | 7.149815 | 1.774251122 |
| *Stch* | 7.0539325 | 1.103931455 |
| *Zfp294* | 7.02505 | 2.069385861 |
| *Pttg1ip* | 7.00486 | 2.866199539 |
| *Chaf1b* | 6.9689 | 2.726250195 |
| *Slc19a1* | 6.846436667 | 1.720399545 |
| *D16Ertd472e* | 6.843091667 | 2.707458728 |
| *Brwd1* | 6.795986 | 1.288616164 |
| *Lss* | 6.70475 | 2.199736512 |
| *Setd4* | 6.576053333 | 1.223920773 |
| *Abcg1* | 6.54453 | 2.338020288 |
| ***Ripk4*** | **6.522025** | **0.91145357** |
| *N6amt1* | 6.51356 | 0.634735 |
| *Prdm15* | 6.43991 | 0.179168 |
| *Usp16* | 6.424695 | 2.765968383 |
| *Col6a2* | 6.387975 | 0.079245457 |
| *Agpat3* | 6.376455 | 1.389066042 |
| ***Zfp295*** | **6.361916667** | **1.521750006** |
| *Wrb* | 6.35745 | 3.397252104 |
| *Dip2a /// LOC100044059* | 6.29647 | 0.27163 |
| ***1810007M14Rik*** | **6.289116667** | **3.119152938** |
| *Ifnar1* | 6.243385 | 2.168010604 |
| *Synj1* | 6.177445 | 1.541887973 |
| *Itsn1* | 6.156338889 | 1.469137954 |
| *Hlcs* | 6.13199 | 0.285105 |
| *Dopey2* | 6.03311 | 1.304102894 |
| *Adarb1* | 5.9712 | 0.359612585 |
| *Wdr4* | 5.8853525 | 2.140709343 |
| *5830404H04Rik* | 5.8414 | 0.676194271 |
| ***Ets2*** | **5.77592** | **2.787594012** |
| *Icosl* | 5.730295 | 2.128299487 |
| *Rsph1* | 5.72139 | 0.204253 |
| *Cryaa* | 5.67826 | 0.152484 |
| *Ubash3a* | 5.52796 | 2.072331986 |
| *Cxadr* | 5.443724 | 1.215082348 |
| *1810043G02Rik* | 5.39137 | 0.353044274 |
| *Trpm2* | 5.384975 | 0.727676517 |
| *Tff2* | 5.28147 | 0.150076 |
| ***Nrip1*** | **5.275076667** | **1.2062462** |
| *Tff3* | 5.23771 | 0.192523 |
| *Ripply3* | 5.14778 | 0.502544 |
| *Ifngr2* | 5.08848 | 0.289828927 |
| *Il10rb* | 5.08403 | 0.262546 |
| *Tiam1* | 5.083373333 | 2.654488501 |
| *4931408A02Rik /// LOC630876* | 5.00361 | 0.141103 |
| *Mx2* | 4.96404 | 0.144945 |
| *Clic6* | 4.93913 | 2.075641245 |
| *Ftcd* | 4.91459 | 0.172576 |
| *Cldn14* | 4.90153 | 0.143671 |
| *B3galt5* | 4.88765 | 1.257595574 |
| *A130042E20Rik* | 4.88299 | 0.293726 |
| *Lrrc3* | 4.77821 | 0.202172 |
| *2310002B14Rik* | 4.77704 | 0.206538 |
| *Usp25* | 4.75899 | 0.578865895 |
| *Ifnar2* | 4.75642 | 2.172294595 |
| *ORF28* | 4.74067 | 0.199701097 |
| *2610028H24Rik* | 4.73107 | 0.168975 |
| *4921526F01Rik* | 4.712425 | 0.497216275 |
| ***Olig1*** | **4.69867** | **0.347647** |
| *Dscam* | 4.6903325 | 1.492275068 |
| ***Snf1lk*** | **4.616425** | **2.846493703** |
| ***Erg*** | **4.541105** | **1.038152963** |
| *App* | 4.4616225 | 1.800856696 |
| *Cyyr1* | 4.45598 | 0.148679 |
| *Krtap8-1* | 4.4115 | 0.167084 |
| *Adamts1* | 4.36928 | 0.384669 |
| *Col6a1* | 4.35004 | 0.151779 |
| ***Runx1*** | **4.3020975** | **1.112645046** |
| *4932438H23Rik* | 4.27836 | 0.177504 |
| *1700027D21Rik* | 4.25232 | 1.122574442 |
| *Kcnj6* | 4.2237 | 0.976934986 |
| *Krtap16-4* | 4.20468 | 0.174145 |
| *Pcp4, Igsf5* | 4.01017 | 0.370325963 |
| *Sh3bgr* | 4.003065 | 0.327086384 |
| *Mx1* | 3.87468 | 0.145676 |
| *Itgb2* | 3.86447 | 0.17977 |
| *Tmprss2* | 3.863164 | 0.477907827 |
| *Bace2* | 3.8580775 | 0.826703722 |
| *Mrap* | 3.790885 | 1.294267039 |
| *S100b* | 3.76893 | 0.920469181 |
| *Chodl* | 3.6244 | 0.151624 |
| *Kcne1* | 3.622683333 | 0.735397441 |
| *Adamts5* | 3.4066125 | 0.697714491 |
| *Slc5a3* | 3.37037 | 0.130329 |
| ***Sim2*** | **3.34456** | **0.763760176** |
| *Ncam2* | 3.342086667 | 0.264564763 |
| *Krtap15* | 3.33255 | 0.148971 |
| *Kcnj15* | 3.30653 | 0.663936408 |
| *ORF63* | 3.30416 | 0.175201 |
| *Rbm11* | 3.19605 | 0.147298 |
| *Prss7* | 3.1533 | 0.133094 |
| *Samsn1* | 3.14729 | 0.143149 |
| *ORF9* | 3.09463 | 0.165194 |
| *Krtap8-2* | 3.05605 | 0.142714 |
| *Umodl1* | 2.94903 | 0.159613 |
| *Grik1* | 2.91322 | 0.24335787 |
| *Kcne2* | 2.89054 | 0.13536 |
| *Cldn8* | 2.71558 | 0.130519 |
| *EG667310 /// LOC545845 /// Sod1* | 2.7084 | 0.116141 |
| ***Olig2*** | **2.65767** | **0.130793** |

The effective genes are shown in blue whereas the silent genes are shown in red. A Gene Set Enrichment Analysis, (GSEA) was performed to compute the significance of this distribution and the results are reported in Figure 3B.
