# Supplementary material for: A mouse embryonic stem cell bank for inducible overexpression of human chromosome 21 genes
Source: Genome Biol. 2010 Jun 22;11(6):R64. doi: 10.1186/gb-2010-11-6-r64 (PMC2911112; doi:10.1186/gb-2010-11-6-r64)
Supplement: Additional file 16 — Summary of results derived from the comparison between the analysis of mES overexpressing effective clones and the transchromosomic Tc1 mouse line. [file gb-2010-11-6-r64-S16.DOC]

**Summary of results derived from the comparison between the analysis of mouse ES overexpressing effective clones and the transchromosomic Tc1 mouse line**

| **Overexpressed Gene Symbol** | **Affy**  **ID** | **UniGene**  **ID** | **Gene**  **Symbol** | **Overxpression exp. log ratio** | **Tc1**  **log ratio** |
| --- | --- | --- | --- | --- | --- |
| ***Aire*** | 1423416_at | Mm.85410 | *Smarcc1* | 0.434 | -1.079 |
|  | 1448259_at | Mm.182434 | *Fstl1* | 0.588 | 0.596 |
|  | 1452784_at | Mm.227 | *Itgav* | 0.344 | 0.611 |
|  | 1452021_a_at | Mm.280029 | *Hes6* | 0.946 | 0.614 |
|  | 1426413_at | Mm.4636 | *Neurod1* | 0.653 | 0.615 |
|  | 1416830_at | Mm.439922 | *0610031J06Rik* | 0.422 | 0.652 |
|  | 1433546_at | Mm.207683 | *Gns* | 0.368 | 0.658 |
|  | 1454670_at | Mm.291274 | *Rere* | 0.401 | 0.682 |
|  | 1416303_at | Mm.294753 | *Litaf* | 0.434 | 0.703 |
|  | 1418402_at | Mm.89940 | *Adam19* | 0.418 | 0.711 |
|  | 1417399_at | Mm.3982 | *Gas6* | 0.477 | 0.719 |
|  | 1416600_a_at | Mm.265744 | *Rcan1* | 0.413 | 0.775 |
|  | 1415671_at | Mm.17708 | *Atp6v0d1* | 0.246 | 0.785 |
|  | 1450376_at | Mm.2154 | *Mxi1* | 0.545 | 0.791 |
|  | 1420502_at | Mm.2734 | *Sat1* | 0.386 | 0.833 |
|  | 1423691_x_at | Mm.358618 | *Krt8* | -0.378 | 0.846 |
|  | 1425248_a_at | Mm.2901 | *Tyro3* | 0.499 | 0.856 |
|  | 1426246_at | Mm.127156 | *Pros1* | 0.519 | 0.872 |
|  | 1416554_at | Mm.5567 | *Pdlim1* | 0.256 | 0.875 |
|  | 1417177_at | Mm.2820 | *Galk1* | 0.413 | 0.939 |
|  | 1450995_at | Mm.2135 | *Folr1* | -0.605 | 0.941 |
|  | 1435275_at | Mm.29625 | *Cox6b2* | 0.662 | 0.943 |
|  | 1452870_at | Mm.220289 | *Apaf1* | 0.474 | 0.967 |
|  | 1448752_at | Mm.1186 | *Car2* | 0.432 | 1.040 |
|  | 1423677_at | Mm.20943 | *Fkbp9* | 0.327 | 1.051 |
|  | 1460242_at | Mm.101591 | *Cd55* | 0.496 | 1.059 |
|  | 1422603_at | Mm.202665 | *Rnase4* | 0.596 | 1.111 |
|  | 1448715_x_at | Mm.249548 | *Ccrn4l /// Cog6 /// Sgip1* | 0.278 | 1.151 |
|  | 1416564_at | Mm.42162 | *Sox7* | -0.698 | 1.157 |
|  | 1433842_at | Mm.45039 | *Lrrfip1* | 0.375 | 1.212 |
|  | 1424797_a_at | Mm.246804 | *Pitx2* | 0.865 | 1.222 |
|  | 1418835_at | Mm.3117 | *Phlda1* | -1.014 | 1.281 |
|  | 1449289_a_at | Mm.163 | *B2m* | 0.741 | 1.297 |
|  | 1433845_x_at | Mm.16479 | *Dusp9* | 0.401 | 1.302 |
|  | 1416749_at | Mm.30156 | *Htra1* | -0.911 | 1.436 |
|  | 1424265_at | Mm.24887 | *Npl* | 0.443 | 1.497 |
|  | 1424683_at | Mm.25311 | *1810015C04Rik* | 0.772 | 1.520 |
|  | 1417845_at | Mm.86421 | *Cldn6* | 0.436 | 1.541 |
|  | 1449106_at | Mm.200916 | *Gpx3* | 0.610 | 1.615 |
|  | 1451310_a_at | Mm.930 | *Ctsl* | 0.418 | 1.700 |
|  | 1418153_at | Mm.303386 | *Lama1* | -0.446 | 1.759 |
|  | 1448392_at | --- | *LOC100046740* | -0.368 | 2.111 |
|  | 1452320_at | Mm.23847 | *Lrp2* | -0.410 | 2.250 |
|  | 1454890_at | Mm.100068 | *Amot* | 0.477 | 2.371 |
| ***Erg*** | 1424719_a_at | Mm.1287 | *Mapt* | -0.624 | -1.278 |
|  | 1422580_at | Mm.390355 | *Myl4* | -1.173 | -0.817 |
|  | 1449347_a_at | Mm.391293 | *Xlr4a /// Xlr4b /// Xlr4c /// Xlr4e* | -0.389 | -0.789 |
|  | 1428092_at | Mm.28270 | *Cdc5l* | -0.400 | -0.758 |
|  | 1419135_at | Mm.1715 | *Ltb* | -0.970 | -0.682 |
|  | 1435420_at | Mm.426473 | *---* | -0.395 | -0.654 |
|  | 1434528_at | Mm.24204 | *Aard* | 0.764 | -0.625 |
|  | 1448866_at | Mm.284592 | *Senp3* | -0.274 | -0.618 |
|  | 1449094_at | Mm.390369 | *Gja7* | -0.382 | -0.579 |
|  | 1423227_at | Mm.14046 | *Krt17* | -1.050 | -0.567 |
|  | 1448326_a_at | Mm.34797 | *Crabp1* | -0.684 | -0.556 |
|  | 1423371_at | Mm.195753 | *Pole4* | -0.656 | -0.556 |
|  | 1432075_a_at | Mm.42257 | *Tekt1* | -0.846 | -0.505 |
|  | 1417246_at | Mm.260144 | *Pzp* | -0.995 | -0.487 |
|  | 1420664_s_at | Mm.3243 | *Procr* | 1.065 | 0.535 |
|  | 1449117_at | Mm.439672 | *Jund1* | 0.944 | 0.564 |
|  | 1452784_at | Mm.227 | *Itgav* | 0.557 | 0.611 |
|  | 1452021_a_at | Mm.280029 | *Hes6* | 0.532 | 0.614 |
|  | 1416257_at | Mm.19306 | *Capn2* | 0.601 | 0.617 |
|  | 1418460_at | Mm.2454 | *Sh3d19* | 0.365 | 0.636 |
|  | 1416891_at | Mm.4390 | *Numb* | 0.328 | 0.666 |
|  | 1448842_at | Mm.241056 | *Cdo1* | 0.376 | 0.683 |
|  | 1418187_at | Mm.260698 | *Ramp2* | 2.725 | 0.700 |
|  | 1416303_at | Mm.294753 | *Litaf* | 0.330 | 0.703 |
|  | 1416735_at | Mm.22547 | *Asah1* | -0.446 | 0.705 |
|  | 1418402_at | Mm.89940 | *Adam19* | -0.602 | 0.711 |
|  | 1438133_a_at | Mm.1231 | *Cyr61* | 2.373 | 0.713 |
|  | 1417399_at | Mm.3982 | *Gas6* | 1.178 | 0.719 |
|  | 1460330_at | Mm.7214 | *Anxa3* | 1.349 | 0.734 |
|  | 1452148_at | Mm.277661 | *Lrpap1* | -0.536 | 0.747 |
|  | 1418655_at | Mm.386762 | *B4galnt1* | -0.472 | 0.749 |
|  | 1448592_at | Mm.20904 | *Crtap* | 0.320 | 0.759 |
|  | 1415938_at | Mm.272 | *Spink3* | -1.409 | 0.761 |
|  | 1429244_at | Mm.268546 | *2610524H06Rik* | -0.397 | 0.762 |
|  | 1422629_s_at | Mm.46014 | *Shroom3* | 0.642 | 0.773 |
|  | 1449438_at | Mm.422657 | *Dpm1* | -0.353 | 0.780 |
|  | 1449279_at | Mm.57225 | *Gpx2* | -1.159 | 0.817 |
|  | 1433883_at | Mm.295124 | *Tpm4* | 0.386 | 0.832 |
|  | 1420502_at | Mm.2734 | *Sat1* | -0.336 | 0.833 |
|  | 1423691_x_at | Mm.358618 | *Krt8* | -1.199 | 0.846 |
|  | 1416554_at | Mm.5567 | *Pdlim1* | -0.220 | 0.875 |
|  | 1416503_at | Mm.2632 | *Lxn* | 2.198 | 0.896 |
|  | 1426648_at | Mm.221235 | *Mapkapk2* | 0.788 | 0.901 |
|  | 1417472_at | Mm.29677 | *Myh9* | 0.530 | 0.920 |
|  | 1416768_at | Mm.10709 | *1110003E01Rik* | -0.668 | 0.928 |
|  | 1423662_at | Mm.25148 | *Atp6ap2* | -0.312 | 0.960 |
|  | 1423429_at | Mm.423066 | *Rhox5* | 0.376 | 0.968 |
|  | 1416657_at | Mm.6645 | *Akt1* | 0.567 | 0.974 |
|  | 1438650_x_at | Mm.378921 | *Gja1* | 0.390 | 1.010 |
|  | 1435493_at | Mm.355327 | *Dsp* | -1.376 | 1.013 |
|  | 1417696_at | Mm.28099 | *Soat1* | 0.313 | 1.014 |
|  | 1448752_at | Mm.1186 | *Car2* | 0.790 | 1.040 |
|  | 1452304_a_at | Mm.320978 | *Arhgef5* | 0.767 | 1.041 |
|  | 1449942_a_at | Mm.274846 | *Ilk* | 0.343 | 1.050 |
|  | 1417090_at | Mm.4876 | *Rcn1* | -0.706 | 1.085 |
|  | 1426195_a_at | Mm.4263 | *Cst3* | 0.596 | 1.096 |
|  | 1434513_at | --- | *Atp13a3* | -0.544 | 1.105 |
|  | 1422603_at | Mm.202665 | *Rnase4* | -1.230 | 1.111 |
|  | 1427442_a_at | Mm.277585 | *App* | 0.606 | 1.139 |
|  | 1416498_at | Mm.4587 | *Ppic* | 0.318 | 1.180 |
|  | 1449256_a_at | Mm.1387 | *Rab11a* | 0.543 | 1.187 |
|  | 1451594_s_at | Mm.272188 | *Serpinb6c* | -2.591 | 1.202 |
|  | 1418835_at | Mm.3117 | *Phlda1* | 1.080 | 1.281 |
|  | 1449289_a_at | Mm.163 | *B2m* | 0.623 | 1.297 |
|  | 1433845_x_at | Mm.16479 | *Dusp9* | 1.402 | 1.302 |
|  | 1418094_s_at | Mm.1641 | *Car4* | 1.461 | 1.305 |
|  | 1415800_at | Mm.378921 | *Gja1* | 0.472 | 1.364 |
|  | 1423523_at | Mm.18651 | *Aass* | -1.470 | 1.387 |
|  | 1417869_s_at | Mm.156919 | *Ctsz* | -0.401 | 1.412 |
|  | 1451791_at | Mm.124316 | *Tfpi* | 0.908 | 1.431 |
|  | 1416749_at | Mm.30156 | *Htra1* | -0.834 | 1.436 |
|  | 1450990_at | Mm.22515 | *Gpc3* | -0.443 | 1.484 |
|  | 1417845_at | Mm.86421 | *Cldn6* | 0.564 | 1.541 |
|  | 1416632_at | Mm.148155 | *Mod1* | -0.630 | 1.577 |
|  | 1449106_at | Mm.200916 | *Gpx3* | 0.577 | 1.615 |
|  | 1451310_a_at | Mm.930 | *Ctsl* | 0.278 | 1.700 |
|  | 1427385_s_at | Mm.253564 | *Actn1* | 1.219 | 1.719 |
|  | 1418153_at | Mm.303386 | *Lama1* | -1.480 | 1.759 |
|  | 1452207_at | Mm.272321 | *Cited2* | 0.515 | 1.864 |
|  | 1452035_at | Mm.738 | *Col4a1* | 1.535 | 1.963 |
|  | 1448392_at | --- | *LOC100046740* | 1.010 | 2.111 |
|  | 1416382_at | Mm.322945 | *Ctsc* | -1.080 | 2.162 |
|  | 1424051_at | Mm.181021 | *Col4a2* | 1.539 | 2.220 |
|  | 1452320_at | Mm.23847 | *Lrp2* | -0.496 | 2.250 |
|  | 1452270_s_at | Mm.313915 | *Cubn* | 0.708 | 2.344 |
| ***Nrip1*** | 1427479_at | Mm.327146 | *Eif1a* | -0.995 | -1.449 |
|  | 1419135_at | Mm.1715 | *Ltb* | 1.477 | -0.682 |
|  | 1434528_at | Mm.24204 | *Aard* | -0.791 | -0.625 |
|  | 1423227_at | Mm.14046 | *Krt17* | 1.676 | -0.567 |
|  | 1431359_a_at | Mm.3336 | *1110007C09Rik* | 0.898 | 0.590 |
|  | 1452127_a_at | Mm.3414 | *Ptpn13* | 1.360 | 0.596 |
|  | 1452021_a_at | Mm.280029 | *Hes6* | 1.679 | 0.614 |
|  | 1416257_at | Mm.19306 | *Capn2* | 0.901 | 0.617 |
|  | 1426400_a_at | Mm.439782 | *Capns1* | -0.421 | 0.623 |
|  | 1418402_at | Mm.89940 | *Adam19* | 0.701 | 0.711 |
|  | 1460330_at | Mm.7214 | *Anxa3* | 1.154 | 0.734 |
|  | 1451335_at | Mm.34609 | *Plac8* | 0.844 | 0.894 |
|  | 1416503_at | Mm.2632 | *Lxn* | 1.765 | 0.896 |
|  | 1431805_a_at | Mm.286600 | *Rhpn2* | 1.103 | 0.963 |
|  | 1452304_a_at | Mm.320978 | *Arhgef5* | 0.731 | 1.041 |
|  | 1426236_a_at | Mm.210745 | *Glul* | 0.476 | 1.150 |
|  | 1424797_a_at | Mm.246804 | *Pitx2* | 3.716 | 1.222 |
|  | 1418094_s_at | Mm.1641 | *Car4* | 1.177 | 1.305 |
|  | 1423523_at | Mm.18651 | *Aass* | 0.802 | 1.387 |
|  | 1416953_at | Mm.393058 | *Ctgf* | 1.223 | 2.009 |
|  | 1448392_at | --- | *LOC100046740* | 0.592 | 2.111 |
| ***Pdxk*** | 1431359_a_at | Mm.3336 | *1110007C09Rik* | 0.670 | 0.590 |
|  | 1449363_at | Mm.2706 | *Atf3* | -0.823 | 0.597 |
|  | 1452021_a_at | Mm.280029 | *Hes6* | 1.024 | 0.614 |
|  | 1418402_at | Mm.89940 | *Adam19* | 0.867 | 0.711 |
|  | 1448904_at | Mm.258484 | *D6Wsu176e* | 0.698 | 0.783 |
|  | 1417177_at | Mm.2820 | *Galk1* | 0.678 | 0.939 |
|  | 1435275_at | Mm.29625 | *Cox6b2* | 0.854 | 0.943 |
|  | 1424797_a_at | Mm.246804 | *Pitx2* | 1.135 | 1.222 |
|  | 1416749_at | Mm.30156 | *Htra1* | -1.022 | 1.436 |
| ***Runx1*** | 1425565_at | Mm.28840 | *Rest* | -0.660 | -1.815 |
|  | 1427479_at | Mm.327146 | *Eif1a* | -0.759 | -1.449 |
|  | 1447377_at | Mm.331671 | *Gm428* | -0.886 | -0.866 |
|  | 1428092_at | Mm.28270 | *Cdc5l* | -0.426 | -0.758 |
|  | 1425675_s_at | Mm.322502 | *Ceacam1* | -0.472 | -0.677 |
|  | 1423227_at | Mm.14046 | *Krt17* | -1.942 | -0.567 |
|  | 1448326_a_at | Mm.34797 | *Crabp1* | -0.400 | -0.556 |
|  | 1432075_a_at | Mm.42257 | *Tekt1* | -0.593 | -0.505 |
|  | 1417246_at | Mm.260144 | *Pzp* | -0.462 | -0.487 |
|  | 1420664_s_at | Mm.3243 | *Procr* | 0.476 | 0.535 |
|  | 1428071_at | Mm.29382 | *1110038D17Rik* | 0.787 | 0.541 |
|  | 1449117_at | Mm.324321 | *Jund1* | 0.396 | 0.564 |
|  | 1448948_at | Mm.17958 | *Rag1ap1* | 0.294 | 0.564 |
|  | 1431359_a_at | Mm.3336 | *1110007C09Rik* | 1.578 | 0.590 |
|  | 1448259_at | Mm.182434 | *Fstl1* | 0.407 | 0.596 |
|  | 1442006_at | Mm.27091 | *---* | 0.667 | 0.597 |
|  | 1420731_a_at | Mm.2020 | *Csrp2* | 0.486 | 0.606 |
|  | 1452784_at | Mm.227 | *Itgav* | 0.337 | 0.611 |
|  | 1426413_at | Mm.4636 | *Neurod1* | -1.053 | 0.615 |
|  | 1436167_at | Mm.18929 | *Shf* | 1.034 | 0.619 |
|  | 1426400_a_at | Mm.423030 | *Capns1* | -0.566 | 0.623 |
|  | 1418460_at | Mm.2454 | *Sh3d19* | 0.722 | 0.636 |
|  | 1421223_a_at | Mm.259702 | *Anxa4* | 0.387 | 0.650 |
|  | 1448434_at | Mm.422969 | *Rnf103* | 0.625 | 0.651 |
|  | 1420873_at | Mm.309867 | *Twf1* | -0.273 | 0.658 |
|  | 1433546_at | Mm.207683 | *Gns* | 0.295 | 0.658 |
|  | 1423225_at | Mm.104491 | *Selk* | -0.263 | 0.666 |
|  | 1429400_at | Mm.254370 | *Clcn5* | 0.607 | 0.666 |
|  | 1460351_at | Mm.280038 | *S100a11* | 0.450 | 0.696 |
|  | 1416808_at | Mm.4691 | *Nid1* | -1.543 | 0.699 |
|  | 1416303_at | Mm.294753 | *Litaf* | 0.579 | 0.703 |
|  | 1460330_at | Mm.7214 | *Anxa3* | 0.570 | 0.734 |
|  | 1422451_at | Mm.379108 | *Mrps21* | 0.212 | 0.745 |
|  | 1418655_at | Mm.386762 | *B4galnt1* | 1.303 | 0.749 |
|  | 1415938_at | Mm.272 | *Spink3* | -2.341 | 0.761 |
|  | 1429244_at | --- | *2610524H06Rik* | -0.344 | 0.762 |
|  | 1415671_at | Mm.17708 | *Atp6v0d1* | 0.337 | 0.785 |
|  | 1454862_at | Mm.211477 | *Phldb2* | 0.326 | 0.791 |
|  | 1438058_s_at | Mm.42855 | *Ptov1* | 0.361 | 0.812 |
|  | 1452281_at | Mm.3770 | *Sos2* | 0.680 | 0.817 |
|  | 1449279_at | Mm.371561 | *Gpx2* | -1.049 | 0.817 |
|  | 1433883_at | Mm.295124 | *Tpm4* | 0.561 | 0.832 |
|  | 1420502_at | Mm.2734 | *Sat1* | -0.754 | 0.833 |
|  | 1423948_at | Mm.247037 | *Bag2* | 0.609 | 0.834 |
|  | 1423691_x_at | Mm.358618 | *Krt8* | 1.236 | 0.846 |
|  | 1426246_at | Mm.127156 | *Pros1* | -0.746 | 0.872 |
|  | 1416554_at | Mm.5567 | *Pdlim1* | 0.482 | 0.875 |
|  | 1426151_a_at | Mm.272264 | *Stx3* | 0.500 | 0.885 |
|  | 1428585_at | Mm.253564 | *Actn1* | 0.395 | 0.894 |
|  | 1451335_at | Mm.34609 | *Plac8* | 1.468 | 0.894 |
|  | 1416503_at | Mm.2632 | *Lxn* | 0.567 | 0.896 |
|  | 1422833_at | Mm.938 | *Foxa2* | -0.257 | 0.899 |
|  | 1433495_at | Mm.273836 | *Glt25d1* | 1.008 | 0.908 |
|  | 1418483_a_at | Mm.281124 | *Ggta1* | -0.326 | 0.917 |
|  | 1417472_at | Mm.29677 | *Myh9* | 0.601 | 0.920 |
|  | 1417177_at | Mm.2820 | *Galk1* | 0.499 | 0.939 |
|  | 1450995_at | Mm.2135 | *Folr1* | -1.273 | 0.941 |
|  | 1451115_at | Mm.1635 | *Pias3* | 0.434 | 0.948 |
|  | 1423662_at | Mm.25148 | *Atp6ap2* | 0.465 | 0.960 |
|  | 1423759_a_at | Mm.330045 | *Tmco1* | -0.227 | 0.960 |
|  | 1452870_at | Mm.220289 | *Apaf1* | 0.479 | 0.967 |
|  | 1423429_at | Mm.423066 | *Rhox5* | -0.555 | 0.968 |
|  | 1416657_at | Mm.6645 | *Akt1* | 0.558 | 0.974 |
|  | 1424726_at | Mm.170023 | *Tmem150* | 0.700 | 1.010 |
|  | 1435493_at | Mm.355327 | *Dsp* | 2.054 | 1.013 |
|  | 1448752_at | Mm.1186 | *Car2* | -0.328 | 1.040 |
|  | 1452304_a_at | Mm.320978 | *Arhgef5* | 0.558 | 1.041 |
|  | 1449942_a_at | Mm.274846 | *Ilk* | 0.322 | 1.050 |
|  | 1423677_at | Mm.20943 | *Fkbp9* | 0.333 | 1.051 |
|  | 1426195_a_at | Mm.4263 | *Cst3* | -0.277 | 1.096 |
|  | 1434513_at | --- | *Atp13a3* | 0.481 | 1.105 |
|  | 1452181_at | Mm.334999 | *Ckap4* | -0.785 | 1.137 |
|  | 1427442_a_at | Mm.277585 | *App* | -0.659 | 1.139 |
|  | 1460346_at | Mm.620 | *Arsa* | 0.314 | 1.140 |
|  | 1426236_a_at | Mm.210745 | *Glul* | -0.662 | 1.150 |
|  | 1416564_at | Mm.42162 | *Sox7* | -0.776 | 1.157 |
|  | 1421654_a_at | Mm.243014 | *Lmna* | 0.965 | 1.171 |
|  | 1422823_at | Mm.235346 | *Eps8 /// LOC632638* | -0.410 | 1.191 |
|  | 1448786_at | Mm.3311 | *1100001H23Rik* | 1.341 | 1.200 |
|  | 1451594_s_at | Mm.272188 | *Serpinb6c* | -1.804 | 1.202 |
|  | 1433842_at | Mm.45039 | *Lrrfip1* | 0.714 | 1.212 |
|  | 1460319_at | Mm.35628 | *Fut8* | 0.356 | 1.218 |
|  | 1415802_at | Mm.9086 | *Slc16a1* | 0.235 | 1.238 |
|  | 1418835_at | Mm.3117 | *Phlda1* | -1.270 | 1.281 |
|  | 1449289_a_at | Mm.163 | *B2m* | 0.591 | 1.297 |
|  | 1433845_x_at | Mm.16479 | *Dusp9* | 0.414 | 1.302 |
|  | 1418094_s_at | Mm.1641 | *Car4* | -0.670 | 1.305 |
|  | 1423523_at | Mm.18651 | *Aass* | -1.454 | 1.387 |
|  | 1425536_at | Mm.272264 | *Stx3* | 0.701 | 1.403 |
|  | 1451791_at | Mm.124316 | *Tfpi* | -1.089 | 1.431 |
|  | 1416749_at | Mm.30156 | *Htra1* | -0.869 | 1.436 |
|  | 1450021_at | Mm.434417 | *Ubqln2* | -0.263 | 1.459 |
|  | 1417109_at | Mm.15801 | *Tinagl* | 1.156 | 1.468 |
|  | 1424683_at | Mm.25311 | *1810015C04Rik* | 0.569 | 1.520 |
|  | 1449106_at | Mm.200916 | *Gpx3* | 1.148 | 1.615 |
|  | 1417408_at | Mm.273188 | *F3* | -0.349 | 1.624 |
|  | 1424114_s_at | Mm.172674 | *Lamb1-1* | -0.347 | 1.624 |
|  | 1451310_a_at | Mm.930 | *Ctsl* | 1.260 | 1.700 |
|  | 1427385_s_at | Mm.253564 | *Actn1* | 1.048 | 1.719 |
|  | 1452207_at | Mm.272321 | *Cited2* | -0.395 | 1.864 |
|  | 1417092_at | Mm.3542 | *Pthr1* | 2.529 | 1.956 |
|  | 1452035_at | Mm.738 | *Col4a1* | -0.363 | 1.963 |
|  | 1416953_at | Mm.393058 | *Ctgf* | -0.248 | 2.009 |
|  | 1448392_at | Mm.291442 | *LOC100046740* | 1.037 | 2.111 |
|  | 1416382_at | Mm.322945 | *Ctsc* | -0.329 | 2.162 |
|  | 1419430_at | Mm.42230 | *Cyp26a1* | 1.258 | 2.246 |
|  | 1452270_s_at | Mm.313915 | *Cubn* | -0.307 | 2.344 |
|  | 1454890_at | Mm.100068 | *Amot* | 0.771 | 2.371 |
|  | 1418365_at | Mm.2277 | *Ctsh* | 0.515 | 2.627 |
| ***Sim2*** | 1452784_at | Mm.227 | *Itgav* | 0.654 | 0.611 |
|  | 1452021_a_at | Mm.280029 | *Hes6* | 0.823 | 0.614 |
|  | 1426413_at | Mm.4636 | *Neurod1* | 1.402 | 0.615 |
|  | 1416830_at | Mm.439922 | *0610031J06Rik* | 0.591 | 0.652 |
|  | 1416808_at | Mm.4691 | *Nid1* | 0.697 | 0.699 |
|  | 1416303_at | Mm.294753 | *Litaf* | 0.651 | 0.703 |
|  | 1416735_at | Mm.22547 | *Asah1* | 0.684 | 0.705 |
|  | 1415938_at | Mm.272 | *Spink3* | -0.879 | 0.761 |
|  | 1420502_at | Mm.2734 | *Sat1* | 0.702 | 0.833 |
|  | 1434314_s_at | Mm.220334 | *Rab11fip5* | 1.112 | 0.863 |
|  | 1426246_at | Mm.127156 | *Pros1* | 0.659 | 0.872 |
|  | 1435275_at | Mm.29625 | *Cox6b2* | 0.852 | 0.943 |
|  | 1460346_at | Mm.620 | *Arsa* | 0.753 | 1.140 |
|  | 1426236_a_at | Mm.210745 | *Glul* | -0.477 | 1.150 |
|  | 1423192_at | Mm.20129 | *Pspc1* | -0.490 | 1.197 |
|  | 1449289_a_at | Mm.163 | *B2m* | 0.843 | 1.297 |
|  | 1419814_s_at | Mm.24662 | *S100a1* | 0.631 | 1.394 |
|  | 1424683_at | Mm.25311 | *1810015C04Rik* | 1.256 | 1.520 |
|  | 1417845_at | Mm.86421 | *Cldn6* | 0.702 | 1.541 |
|  | 1451310_a_at | Mm.930 | *Ctsl* | 0.569 | 1.700 |

This table lists the gene expression values used in the comparison between Cell Bank overexpression experiments and Tc1 expression data. These last two values correspond to the *y* and *x* values, respectively, of the points forming the scatter plots in Additional file 17. Each section in the table corresponds to a single overexpression experiment, indicated in the leftmost column. The probesets included in each section are those whose change in expression was found to be significant in both that experiment and in the Tc1 microarray data (significance assessed separately for the two sets of data). The values are the base 2 logarithm of the treated vs. untreated ratio.
