# Supplementary material for: A mouse embryonic stem cell bank for inducible overexpression of human chromosome 21 genes
Source: Genome Biol. 2010 Jun 22;11(6):R64. doi: 10.1186/gb-2010-11-6-r64 (PMC2911112; doi:10.1186/gb-2010-11-6-r64)
Supplement: Additional file 18 — A complete list of GO terms significantly enriched in the subsets of genes differentially expressed after overexpression of 11 out of 13 silent genes. [file gb-2010-11-6-r64-S18.DOC]

**A complete list of Gene Ontology (GO) terms significantly enriched in the subsets of genes differentially expressed after overexpression of eleven silent genes**

| **Gene** | **Gene Onthology** | **FDR** | **Fold** |
| --- | --- | --- | --- |
| **Symbol** | **terms** |  | **Enrichment** |
| *Bach1* | stress-activated protein kinase signaling pathway | 0.8 | 3.0 |
|  | negative regulation of programmed cell death | 1.3 | 1.9 |
|  | regulation of JNK cascade | 3.7 | 4.1 |
| *Dscr1-Rcan1* | axon guidance | 0.1 | 3.1 |
|  | positive regulation of metabolic process | 2.4 | 1.6 |
|  | positive regulation of transcription | 3.5 | 1.7 |
|  | regulation of cell activation | 4.5 | 2.4 |
| *DYRK1A* | translation | 0.0 | 1.8 |
|  | ribosome biogenesis and assembly | 0.0 | 2.0 |
|  | translational elongation | 0.8 | 3.2 |
|  | biopolymer biosynthetic process | 4.6 | 2.3 |
| *Gabpa* | DNA metabolic process | 0.0 | 1.5 |
|  | ribosome biogenesis and assembly | 0.0 | 2.0 |
|  | translation | 0.0 | 1.5 |
|  | DNA replication | 0.0 | 1.9 |
|  | cell cycle phase | 0.1 | 1.6 |
|  | chromosome organization and biogenesis | 0.5 | 1.5 |
|  | cytoskeleton-dependent intracellular transport | 1.3 | 1.7 |
|  | mitosis | 1.7 | 1.6 |
| *Hunk* | translation | 0.0 | 2.1 |
|  | ribosome biogenesis and assembly | 0.0 | 3.1 |
|  | RNA processing | 0.7 | 1.5 |
|  | response to unfolded protein | 2.0 | 2.2 |
| *Olig1* | spermatid nuclear elongation | 1.6 | 56.7 |
| *Pfkl* | intracellular protein transport | 1.8 | 1.5 |
|  | ribosome biogenesis and assembly | 2.9 | 2.0 |
| *Pknox1* | homophilic cell adhesion | 0.1 | 3.2 |
| *Ripk4* | response to virus | 0.5 | 3.0 |
|  | endocytosis | 3.1 | 1.8 |
| *SNF1LK* | endocytosis | 0.0 | 4.8 |
|  | neurite morphogenesis | 0.2 | 2.5 |
|  | neurite development | 0.4 | 2.2 |
|  | establishment of protein localization | 3.6 | 1.5 |
| *ZFP295* | translation | 0.0 | 3.0 |
|  | ribosome biogenesis and assembly | 0.0 | 4.2 |
|  | response to unfolded protein | 2.8 | 2.8 |
|  | regulation of carbohydrate biosynthetic process | 4.3 | 12.1 |
|  | macrophage activation | 4.8 | 7.6 |

The data in this table was collected by a GO analysis performed on the list of differentially expressed genes (reported in Additional files 6 to 12) using the DAVID online tool in those experiments involving the overexpression of silent genes, by using a more sensitive statistical method than the standard t-test approach. The method was the Bayesian Analysis of Variance for Microarrays, a Bayesian spike and slab hierarchical model, as implemented in the BAMarray tool (BAMarray 3.0). Transcriptional changes were detected for eleven out of thirteen silent genes, despite the low fold change of differentially expressed genes, which therefore could include more False Positives than the standard t-test. For the two remaining silent genes (*Ets2* and *1810007M14Rik*) no significant GO terms were found.

In the table we report the gene symbols, a subsets of significant GO terms for the eleven silent genes, the FDR and the Fold Enrichment values for each GO term.
