# Supplementary material for: A mouse embryonic stem cell bank for inducible overexpression of human chromosome 21 genes
Source: Genome Biol. 2010 Jun 22;11(6):R64. doi: 10.1186/gb-2010-11-6-r64 (PMC2911112; doi:10.1186/gb-2010-11-6-r64)
Supplement: Additional file 19 — GO enrichment analysis for five (Bach1, Dscr1-Rcan1, DYRK1A, Gabpa and SNF1LK) out of thirteen silent genes, as assessed by microarray analysis. In this table we report the GO enrichment analysis for five out of thirteen silent genes (Bach1, Dscr1-Rcan1, DYRK1A, Gabpa, SNF1LK); supporting references for a subset of significant biological processes identified by the GO analysis are given. [file gb-2010-11-6-r64-S19.DOC]

**Gene Onthology enrichment analysis for five (*Bach1, Dscr1-Rcan1, DYRK1A, Gabpa* and *SNF1LK*) out of thirteen silent genes, as assessed by microarray analysis**

| **Gene** | **Gene Onthology** | **FDR** | **Fold** | **References** |
| --- | --- | --- | --- | --- |
| **Symbol** | **terms** |  | **Enrichment** |  |
| *Bach1* | stress-activated protein kinase signaling pathway | 0.8 | 3.0 | (Alam et al. 2004) (Mense et al. 2006) |
|  | regulation of JNK cascade | 3.7 | 4.1 | (Goven et al. 2009) |
| *Dscr1-Rcan1* | positive regulation of metabolic process | 2.4 | 1.6 | (Silveira et al. 2004) (Hilioti et al. 2004) |
|  | regulation of cell activation | 4.5 | 2.4 | (Wu et al. 2007) (Kyttälä et al. 2009) (Minami et al. 2009) |
| *DYRK1A* | translation | 0.0 | 1.8 | (Kimura et al. 2007) |
|  | biopolymer biosynthetic process | 4.6 | 2.3 | (Skurat et al. 2004) |
| *Gabpa* | cell cycle phase | 0.1 | 1.6 | (Yang et al. 2007) |
| *SNF1LK* | establishment of protein localization | 3.6 | 1.5 | (Katoh et al.2004) |

The data in this table was collected by a GO analysis performed on the list of differentially expressed genes (using the DAVID online tool restricting the output to Biological Process terms of level 4 and 5; with a significance threshold of FDR<5% and Fold Enrichment ≥1.5%) in those experiments involving the overexpression of five silent genes, by using a more sensitive statistical method than the standard t-test approach (see text and Additional file 18 for further details).

The significant GO terms listed are concordant with the known function of these genes as assessed by literature-based evidences (see “References” column).
