# Supplementary material for: A mouse embryonic stem cell bank for inducible overexpression of human chromosome 21 genes
Source: Genome Biol. 2010 Jun 22;11(6):R64. doi: 10.1186/gb-2010-11-6-r64 (PMC2911112; doi:10.1186/gb-2010-11-6-r64)
Supplement: Additional file 20 — Differential protein expression variation in mES cells overexpressing Runx1. In this table we report the complete list of the proteins whose expression changed following the induction of Runx1 [file gb-2010-11-6-r64-S20.DOC]

**Differential protein expression variation in mouse ES cells overexpressing Runx1**

| **Spot ID*** | **Gene Symbol** | **Protein Name** | **Protein expression ratio** | **St_Error** | **MW [KDa]** |
| --- | --- | --- | --- | --- | --- |
| 470 | *Eno1* | enolase 1, alpha non-neuron | 0.36 | 0,02908794 | 47.09 |
| 2887 | *Bckdha* | 3-methyl-2-oxobutanoate dehydrogenase (lipoamide) | 0.36 | 0,002660518 | 42.85 |
| 2927 | *Uchl5* | Ubiquitin carboxyl-terminal hydrolase isozyme L5 (EC 3.4.19.12) (UCH-L5) (Ubiquitin thioesterase L5) (Ubiquitin C-terminal hydrolase UCH37) | 0.53 | 0,003106821 | 37.59 |
| 3039 | *Txndc7* | Txndc7 protein | 0.56 | 0,007605688 | 48 |
| 3214 | *Uchl1* | ubiquitin carboxy-terminal hydrolase L1 | 0.58 | 0,001820098 | 24.82 |
| 2358 | *Hspa9a* | Stress-70 protein, mitochondrial precursor (75 kDa glucose-regulated protein) (GRP 75) (Peptide-binding protein 74) (PBP74) (P66 MOT) (Mortalin) | 0.59 | 0,001751826 | 73.48 |
| 2971 | *Ubqln1* | Ubiquilin-1 (Protein linking IAP with cytoskeleton 1) (PLIC-1) | 0.59 | 0,003873001 | 61.93 |
| 662 | *Dppa4* | developmental pluripotency associated 4 isoform 1 | 0.65 | 0,001635495 | 32.67 |
| 3549 | *Igf2bp2* | Insulin-like growth factor 2 mRNA-binding protein 2 (IGF-II mRNA-binding protein 2) (IMP-2) | 0.65 | 0,001698618 | 65.54 |
| 1114 | *Nme1* | tumor metastatic process-associated protein NM23; Nucleoside-diphosphate kinase 1 | 0.7 | 0,007883204 | 18.67 |
| 1416 | *Rbm3* | Rbm3 protein | 0.7 | 0,003635335 | 16.59 |
| 2503 | *Prx* | Periaxin | 0.73 | 0,000912732 | 14.75 |
| 2836 | *Sipa1l1* | Signal-induced proliferation-associated 1-like protein 1 | 0.74 | 0,006966616 | 19.69 |
| 3235 | *Stam* | Signal transducing adapter molecule 1 (STAM-1) | 0.74 | 0,002288916 | 59.73 |
| 2813 | *Ubfd1* | D7Wsu128e protein [Mus musculus]; ubiquitin-binding protein homolog | 0.75 | 0,001457718 | 33.42 |
| 3198 | *Uchl3* | ubiquitin carboxyl-terminal esterase L3 (ubiquitin thiolesterase) | 0.75 | 0,005116916 | 26.16 |
| 3465 | *Ranbp1* | Ran/TC4-binding protein 1 | 0.75 | 0,009808444 | 23.56 |
| 425 | *Lap3* | Cytosol aminopeptidase (Leucine aminopeptidase) (LAP) (Leucyl aminopeptidase) (Leucine aminopeptidase 3) (Proline aminopeptidase)(Prolyl aminopeptidase) | 0.76 | 0,001612132 | 56.1 |
| 2458 | *Rad23b* | UV excision repair protein RAD23 homolog B (mHR23B) (XP-C repair-complementing complex 58 kDa protein) (p58) | 0.77 | 0,001238707 | 43.49 |
| 2512 | *Hspd1* | 60 kDa heat shock protein, mitochondrial precursor (Hsp60) (60 kDa chaperonin) (CPN60) (Heat shock protein 60) (HSP-60) (Mitochondrial matrix protein P1) (HSP-65) | 0.78 | 0,001323269 | 60.91 |
| 2612 | *Hnrpf* | Heterogeneous nuclear ribonucleoprotein F (hnRNP F) | 0.78 | 0,038638554 | 45.7 |
| 3288 | *Pebp1* | phosphatidylethanolamine binding protein [Mus musculus]; similar to hippocampal cholinergic neurostimulating peptide precursor protein | 0.79 | 0,001217721 | 20.84 |
| 2430 | *Hspa9a* | Stress-70 protein, mitochondrial precursor (75 kDa glucose-regulated protein) (GRP 75) (Peptide-binding protein 74) (PBP74) (P66 MOT) (Mortalin) | 0.8 | 0,003666427 | 73.48 |
| 3410 | *Eif1a* | eukaryotic translation initiation factor 1A, Y-linked | 0.8 | 0,001595063 | 16.45 |
| 414 | *Dld* | Dihydrolipoyl dehydrogenase, mitochondrial precursor (Dihydrolipoamide dehydrogenase) | 1.25 | 0,002485445 | 54.23 |
| 403 | *Pkm2* | Pyruvate kinase isozyme M2 | 1.26 | 0,007045858 | 57.85 |
| 841 | *Mdh* | Malate dehydrogenase, mitochondrial precursor | 1.26 | 0,003919967 | 35.57 |
| 941 | *Atp5c1* | ATP synthase gamma chain, mitochondrial precursor | 1.28 | 0,006366364 | 32.86 |
| 2923 | *Npm1* | nucleophosmin 1 | 1.28 | 0,011272927 | 32.54 |
| 169 | *Sc22b* | Vesicle-trafficking protein SEC22b (SEC22 vesicle-trafficking protein homolog B) | 1.29 | 0,028204785 | 24.72 |
| 631 | *Kbl* | 2-amino-3-ketobutyrate coenzyme A ligase, mitochondrial precursor (AKB ligase) | 1.29 | 0,003948236 | 44.9 |
| 2355 | *Ass1* | Argininosuccinate synthase (Citrulline--aspartate ligase) | 1.29 | 0,020210946 | 46.55 |
| 935 | *Bdh1* | D-beta-hydroxybutyrate dehydrogenase, mitochondrial precursor | 1.31 | 0,012234858 | 38.26 |
| 3459 | *Alb* | Albumin 1 | 1.34 | 0,011000517 | 68.64 |
| 376 | *Hnrpm* | Heterogeneous nuclear ribonucleoprotein M | 1.39 | 0,002477125 | 77.59 |
| 3448 | *Cct5* | T-complex protein 1 subunit epsilon (TCP-1-epsilon) (CCT-epsilon) | 1.41 | 0,030089341 | 59.58 |
| 3146 | *Psme1* | proteasome (prosome, macropain) 28 subunit, alpha | 1.42 | 0,025292801 | 28.65 |
| 645 | *Serpinh1* | serine (or cysteine) proteinase inhibitor, clade H, member 1 | 1.44 | 0,008721166 | 46.56 |
| 2920 | *Ldhb* | lactate dehydrogenase 2, B chain | 1.46 | 0,012882218 | 36.54 |
| 3267 | *Mical2* | microtubule associated monoxygenase, calponin and LIM domain containing 2 | 1.48 | 0,028932388 | 110.1 |
| 2861 | *Hspa8* | heat shock 70 protein | 1.5 | 0,033677875 | 70.7 |
| 644 | *Idh2* | isocitrate dehydrogenase 2 (NADP+), mitochondrial | 1.52 | 0,008471043 | 51 |
| 3562 | *Cotl1* | coactosin-like 1 | 1.62 | 0,031652629 | 15.9 |
| 3567 | *Txn1* | Thioredoxin 1 | 1.64 | 0,009527197 | 11.6 |
| 875 | *Mrpl1* | 39S ribosomal protein L1, mitochondrial precursor (L1mt) | 1.69 | 0,033242736 | 34.9 |
| 3653 | *S100a11* | S100 calcium binding protein A11 (calizzarin) | 1.89 | 0,071663129 | 11 |
| 1509 | *Set* | SET translocation | 2.28 | 0,131326453 | 33.3 |
| 2314 | *Otc* | ornithine transcarbamylase | 2.28 | 0,599716175 | 39.8 |
| 3144 | *1-Sep* | septin 1 | 2.28 | 0,6865517 | 41.8 |
| 1134 | *Gsto1* | glutathione S-transferase omega 1 | 2.77 | 0,423025927 | 27.4 |
| 139 | *Akap8* | A kinase anchor protein 8 | 2.81 | 0,805543924 | 76.2 |
| 3588 | *Fabp3* | fatty acid binding protein 3, muscle and heart | 3.05 | 1,036229474 | 14.8 |
| 3078 | *Apoe* | apolipoprotein E | 3.28 | 0,199744068 | 33.2 |
| 2303 | *Usp5* | Ubiquitin specific protease 5 (isopeptidase T) | 3.99 | 0,882772139 | 95.7 |

Note: * Spot ID corresponds to Table S13

The data in this table was collected by the full proteomic analysis carried out on the total protein extracts of two mES clones (E6 and E7) overexpressing Runx1, grown in medium deprived of Tc for 48hrs, using high resolution large-gel 2-dimensional electrophoresis method (2DGE) followed by the protein identification performed with database-assisted Mass Spectrometry. The induction of the protein Runx1 changes the expression of at least 54 proteins: 24 proteins were consistently down-regulated; 30 were up-regulated. The differential expression of each protein is reported as differential protein expression ratio (t48hrs-mean/t0hrs-mean). The statistic significance was accessed by student’s T-test, with p<0.05, and in addition, only if there is an expression alteration over 20%. The standard error (St_Error) following the differential protein expression analysis is also reported.
