# Supplementary material for: A mouse embryonic stem cell bank for inducible overexpression of human chromosome 21 genes
Source: Genome Biol. 2010 Jun 22;11(6):R64. doi: 10.1186/gb-2010-11-6-r64 (PMC2911112; doi:10.1186/gb-2010-11-6-r64)
Supplement: Additional file 21 — Primer pairs used in PCR. [file gb-2010-11-6-r64-S21.DOC]

**Primer pairs used in PCR**

| 1810007M14Rik-fw: 5'-GGC GCG CCA TGG CAC CAA ATC TTG ATT CCT-3' |
| --- |
| 1810007M14Rik-rev: 5'-TTA ATT AAT TTT CCT TCA ATC AGA GAC TTG-3' |
| Aire-fw: 5'-GGC GCG CCA TGG CAG GTG GGG ATG GAA TGC-3' |
| Aire-rev: 5'-TTA ATT AAG GAA GAG AAG GGT GGT GTC TCG-3' |
| Atp5j-fw: 5'-GGC GCG CCA TGG TTC TGC AGA GGA TCT TC-3' |
| Atp5j-rev: 5'-TTA ATT AAT CAG GAC TGG GGT TTG TCG AT-3' |
| Atp5o-fw: 5'-GGC GCG CCA TGG CCG CGC CTG CAG CGT CCG-3' |
| Atp5o-rev: 5'-TTA ATT AAT CAG AGC ATC TCC CGC ATG GCC-3' |
| Bach1-fw: 5'-GGC GCG CCA TGT CTG TGA GTG AGA GTG CGG-3' |
| Bach1-rev: 5'-TTA ATT AAC TCG TCA GTA GTG CAC TTG TCA-3' |
| Cct8-fw: 5'-GGC GCG CCA TGG CGC TTC ACG TCC CCA AGG-3' |
| Cct8-rev: 5'-TTA ATT AAT CAG TCA TTC TGG TCG TCA TCC-3' |
| Cstb-fw: 5'-TTA ATT AAA TGA AGT GTG GCG CGC CAT CT-3' |
| Cstb-rev: 5'-TTA ATT AAT CAG AAG TAG GAG AGC TCA TC-3' |
| Dnmt3l-fw: 5'-GGC GCG CCA TGG GTT CCC GGG AGA CAC CTT-3' |
| Dnmt3l-rev: 5'-TTA ATT AAC TAA AGA GGA AGT GAG TTT TGG-3' |
| Dscr1-fw: 5'-GGC GCG CCA TGC ATT TTA GGG ACT TTA GCT-3' |
| Dscr1-rev: 5'-TTA ATT AAG CTG AGG TGG ATG GGT GTG TAC-3' |
| Dscr2-fw: 5'-GGC GCG CCA TGG CTG CCA CGT TCT TTG GCG-3' |
| Dscr2-rev: 5'-TTA ATT AAT CAT GTG TAA ATG TTG CTC TGA-3' |
| DYRK1A-fw: 5'-GGC GCG CCA TGC ATA CAG GAG GAG AGA CT-3' |
| DYRK1A-rev: 5'-TTA ATT AAC GAG CTA GCT ACA GGA CTC TG-3' |
| Erg-fw: 5'-GGC GCG CCA TGA TCC AGA CTG TAC CTG ACC-3' |
| Erg-rev: 5'-TTA ATT AAG TAG TAG GTG CCC AGG TGA GAG-3' |
| Ets2-fw: 5'-GGC GCG CCA TGA ATG ACT TTG GAA TCA AGA-3' |
| Ets2-rev: 5'-TTA ATT AAG TCT TCT GTA TCA GGC TGG ACG-3' |
| Gabpa-fw: 5'-GGC GCG CCA TGA CTA AGA GAG AAG CAG AAG-3' |
| Gabpa-rev: 5'-TTA ATT AAA ATC TCT TTG TCT GCC TGT AGA-3' |
| Gart-fw: 5'-GGC GCG CCA TGG CAG CCC GAG TTC TTG TCA-3' |
| Gart-rev: 5'-TTA ATT AAT CAC TGC TCC TTG GCC CAG TGG-3' |
| Hunk-fw: 5'-GGC GCG CCA TGC CGG CAG CGG CGG GGG AC-3' |
| Hunk-rev: 5'-TTA ATT AAA CAC TGG CCC TTG ACA CCG TC-3' |
| Morc3-fw: 5'-GGC GCG CCA TGG CGG CGC AGC CAC CCA CCG-3' |
| Morc3-rev: 5'-TTA ATT AAT TAA GTA CTG CTG ATC TCG CTC-3' |
| Mrpl39-fw: 5'-GGC GCG CCA TGC GGA ATG ATC TGT TTA ACA-3' |
| Mrpl39-rev:5'-TTA ATT AAC TAC TGG GTG GAT TCG GTG TTC-3' |
| Nrip1-fw: 5'-GGC GCG CCA TGA CTC ATG GAG AAG AGC TTG-3' |
| Nrip1-rev: 5'-TTA ATT AAT TCT GAC TCT TTT TTT ATG GTG-3' |
| Olig1-fw: 5'-GGC GCG CCA TGT ACT ATG CGA TTT CCC AGG-3' |
| Olig1-rev: 5'-TTA ATT AAC TTG GAG AAC TGG GCC TGC ACC-3' |
| Olig2-fw: 5'-GGC GCG CCA TGG ACT CGG ACG CCA GCC TGG-3' |
| Olig2-rev: 5'-TTA ATT AAC TTG GCG TCG GAG GTG AGG CGC-3' |
| Pdxk-fw: 5'-GGC GCG CCA TGG AGG GCG AAT GCC GGG TGC-3' |
| Pdxk-rev: 5'-TTA ATT AAC AGC ACT GTG GCC TGC ACG ACG-3' |
| Pfkl-fw: 5'-GGC GCG CCA TGG CTA CCG TGG ACC TGG AGA-3 |
| Pfkl-rev: 5'-TTA ATT AAT TCT GTG ACC GGA CTG AAG GCC-3' |
| Pknox1-fw: 5'-GGC GCG CCA TGA TGG CGA CAC AGA CGC TAAG-3' |
| Pknox1-rev: 5'-TTA ATT AAC TAC TGA AGG GAG TCG CTG TTC-3' |
| Pttg1ip-fw: 5'-GGC GCG CCA TGG CGC CTG CTA ACC TTG GG-3' |
| Pttg1ip-rev: 5'-TTA ATT AAT TAG AAC TTC TCA TAC GGG TT-3' |
| Ripk4-fw: 5'-GGC GCG CCA TGG AGG GCG AGG GCC GGG GC3' |
| Ripk4-rev: 5'-TTA ATT AAG GTC TTG CTG CGT CGG AGC AA-3' |
| Rrp1-fw: 5'-GGC GCG CCA TGG TTC CCG GAG TGC CGC TCC-3' |
| Rrp1-rev: 5'-TTA ATT AAT CAC TCT GCA CTC TGT GAT GAC-3' |
| Runx1-fw: 5'-CTC GAG ATG GCT TCA GAC AGC ATT TTT GAG-3' |
| Runx1-rev: 5'-GCG GCC GCT CAG TAG GGC CGC CAC ACG GCC-3' |
| Sim2-fw: 5'-GGC GCG CCA TGA AGG AGA AGT CCA AAA ATG-3' |
| Sim2-rev: 5'-TTA ATT AAC CTG CCG TTG GTG ATG ATG ACC-3' |
| SNF1LK-fw: 5'-GGC GCG CCA TGG TTA TCA TGT CGG AGT TC-3' |
| SNF1LK-rev: 5'-TTA ATT AAC TGC ACC AGG ACA AAC GTG CC-3' |
| Sod1-fw: 5'-GGC GCG CCA TGG CGA TGA AAG CGG TGT GCG-3' |
| Sod1-rev: 5'-TTA ATT AAT TAC TGC GCA ATC CCA ATC ACT-3' |
| ZFP295-fw: 5'-GGC GCG CCA TGG AGG GAT TAC TGC ATT AC-3' |
| ZFP295-rev: 5'-TTA ATT AAA TTG TGT GTT TGT TCG TGA CT-3' |
